# The Active Compounds and Therapeutic Mechanisms of Pentaherbs Formula for Oral and Topical Treatment of Atopic Dermatitis Based on Network Pharmacology

**DOI:** 10.3390/plants9091166

**Published:** 2020-09-09

**Authors:** Man Chu, Miranda Sin-Man Tsang, Ru He, Christopher Wai-Kei Lam, Zhi Bo Quan, Chun Kwok Wong

**Affiliations:** 1 Faulty of Medical Technology, Shaanxi University of Chinese Medicine, Xianyang 712046, China; 2071013@sntcm.edu.cn (M.C.); 517070302443@email.sntcm.edu.cn (R.H.); 2 Department of Chemical Pathology, The Chinese University of Hong Kong, Prince of Wales Hospital, Shatin, NT, Hong Kong 999077, China; tsangsinman0128@gmail.com; 3 State Key Laboratory of Research on Bioactivities and Clinical Applications of Medicinal Plants and Institute of Chinese Medicine, The Chinese University of Hong Kong, Shatin, NT, Hong Kong 999077, China; 4 State Key Laboratory of Quality Research in Chinese Medicines and Faculty of Medicine, Macau University of Science and Technology, Macau 999078, China; wklam@must.edu.mo; 5 Li Dak Sum Yip Yio Chin R & D Centre for Chinese Medicine, The Chinese University of Hong Kong, Shatin, NT, Hong Kong 999077, China

**Keywords:** pentaherbs, atopic dermatitis, active compounds, therapeutic mechanisms

## Abstract

To examine the molecular targets and therapeutic mechanism of a clinically proven Chinese medicinal pentaherbs formula (PHF) in atopic dermatitis (AD), we analyzed the active compounds and core targets, performed network and molecular docking analysis, and investigated interacting pathways. Information on compounds in PHF was obtained from the Traditional Chinese Medicine Systems Pharmacology (TCMSP) database, and target prediction was performed using the Drugbank database. AD-related genes were gathered using the GeneCards and Online Mendelian Inheritance in Man (OMIM) databases. Network analysis was performed by Cytoscape software and protein-protein interaction was analyzed by the Search Tool for the Retrieval of Interacting Genes/Proteins (STRING). The Database for Annotation, Visualization and Integrated Discovery (DAVID) Bioinformatics Resources were applied for the enrichment analysis of the potential biological process and pathways associated with the intersection targets between PHF and AD. Autodock software was used to perform protein compound docking analysis. We identified 43 active compounds in PHF associated with 117 targets, and 57 active compounds associated with 107 targets that form the main pathways linked to oral and topical treatment of AD, respectively. Among them, quercetin, luteolin, and kaempferol are key chemicals targeting the core genes involved in the oral use of PHF against AD, while apigenin, ursolic acid, and rosmarinic acid could be used in topical treatment of PHF against AD. The compound–target–disease network constructed in the current study reveals close interactions between multiple components and multiple targets. Enrichment analysis further supports the biological processes and signaling pathways identified, indicating the involvement of IL-17 and tumor necrosis factor signaling pathways in the action of PHF on AD. Our data demonstrated the main compounds and potential pharmacological mechanisms of oral and topical application of PHF in AD.

## 1. Introduction

Atopic dermatitis (AD) is a common allergic skin disease affecting all ages of people in the world, especially children [1,2]. The prevalence of AD has been increasing globally. The onset of AD occurs before the age of five in about 70% of AD patients [3,4]. In Hong Kong, more than 19% of adolescent schoolchildren are suffering from AD [5]. While AD is not directly life-threatening, the chronic disease causes a significant burden on daily lives, affecting quality of life for the individual, their families, and the communities they live in, both socially and financially. It is characterized by redness, cracking, dryness, itchiness, thickening, and inflammation of the skin, accompanying the apoptosis of keratinocytes that compromises the quality of life of patients [6]. The pathogenesis of AD involves the abnormal T helper (Th)2 lymphocytes-related cytokine interleukin (IL)-4, IL-5, and IL-13 and serum immunoglobin-(Ig)E levels in response to environmental allergens, which is putatively associated with the disturbance of the epidermal barrier [7]. The dysfunction of the epidermal barrier leads to the penetration of allergens that drives dendritic cells to initiate Th2 polarization and eosinophilic inflammation [8,9].

There is no definitive cure for AD, and the present clinical treatment of AD mainly includes topical anti-inflammatory agent corticosteroids and calcineurin inhibitors. As a potent drug for inflammatory diseases, corticosteroids have been shown to be effective for both acute and chronic AD, though treatment with systemic corticosteroids is not recommended [10]. However, prolonged use of topical corticosteroids can lead to undesirable side effects, including irreversible skin thinning as well as systemic suppression of adrenal function and growth induced by absorption [3]. Topical calcineurin inhibitors, including tacrolimus and pimecrolimus, have been shown to alleviate AD symptoms by suppressing the gene transcription of critical AD-related cytokines such as IL-2, IL-4, and IL-5. However, an alert issued by the Food and Drug Administration demonstrated a potential risk between calcineurin inhibitors and cancer [11,12,13]. Traditional Chinese Medicine (TCM) has become a more widely accepted treatment for immunomodulation of chronic diseases with fewer side effects. According to the concept of TCM, AD can be classified as “cubito-popliteal eczema”, “itching eruptions due to wind-heat in blood” and “prurigo”, namely “Si Wan Feng” in Chinese. Unlike Western medicine, Chinese medicine emphasizes the importance of internal treatment in the control of disease, and is often prescribed as a decoction. However, the mechanisms of multicomponent and multitarget TCM treatment from an integrative and holistic point of view should be explored and revealed.

Pentaherbs formula (PHF), an improved herbal formula with five Chinese medicines, was developed with reference to their specific properties by the Institute of Chinese Medicine, The Chinese University of Hong Kong (commercial name: Shi Min Qing capsule). PHF is composed of five herbs, namely Cortex Moutan (CM), Cortex Phellodendri (CP), Flos Lonicerae (FL), Herba Menthae (HM), and Rhizoma Atractylodis (RA), which are the root bark of *Paeonia suffruticosa* Andr. (*Ranunculaceae*), the bark of *Phellodendron chinensis* Schneid. (*Rutaceae*), the flower of *Lonicera japonica* Thunb. (*Capri-foliaceae*), the aerial part of *Mentha haplocalyx* Briq. (*Labiatae*) and the rhizome of *Atractylodes lancea* (Thunb.) DC. (*Compositae*), respectively. In the concept of TCM, these five herbs can clear the heat, resolve toxicity, dispel wind-heat from the exterior, dry dampness, drain fire, and cool the blood, and were thus applied for the treatment of AD. Previous clinical trials performed by our group showed that moderate-to-severe AD children had significant improvement in the quality of life after a 12-week treatment with PHF [14]. Their concurrent use of topical corticosteroids was also reduced [14]. PHF capsules were also shown to be well tolerated by children and adverse effects were not noticeable clinically [15]. The findings suggested that PHF is a potential alternative adjunct therapy for treating AD. The anti-inflammatory actions of PHF were further elucidated in vitro. With an increased ratio of Danpi in the formula, inflammatory cytokines IL-6 and IL-1 released from human mast cells HMC-1 was greatly inhibited [16]. Our previous studies showed that PHF, Danpi, and one of its compounds—gallic acid—can suppress AD-related pro-inflammatory cytokines/chemokines from human basophils, and interfere with the maturation of monocyte-derived dendritic cells, which are crucial cells for initiating allergic sensitization [17,18]. Oral and topical treatment of PHF can also reduce inflammation on the challenged ears of our oxazolone-mediated dermatitis mice [19]. We also demonstrated the anti-inflammatory activities of three ingredients in PHF in eosinophils/dermal fibroblasts coculture upon IL-31 and IL-33 stimulation [19]. Together, PHF treatment may be a novel modality for managing AD. However, its pharmacological mechanism has not been elucidated completely as PHF is a multicomponent and multitargeted agent that demonstrates therapeutic efficacy within the body system.

Network pharmacology interprets the disease pathogenesis from the perspective of systems biology and the balance of biological networks, and hence provides guidance for the discovery of new drugs based on high-throughput data technologies with computational data analysis [20,21]. Therefore, it has been utilized to identify molecular mechanisms involved in the therapeutic actions of herbal medicine and its multitargeted compounds in biological networks [22]. Based on our previous studies, we now aim at applying a comprehensive network pharmacology approach to predict active compounds and molecular targets of PHF for both oral and topical treatment against AD. Moreover, the putative targets of PHF against AD and the associated potential pathways of how PHF exerts its therapeutic effects on AD will be explored in the present study.

### 1.1. Active Compounds and Compound Targets in PHF

**Figure 1:**
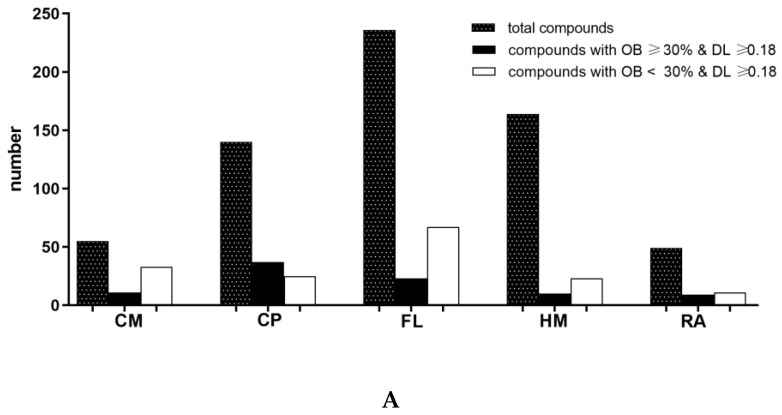

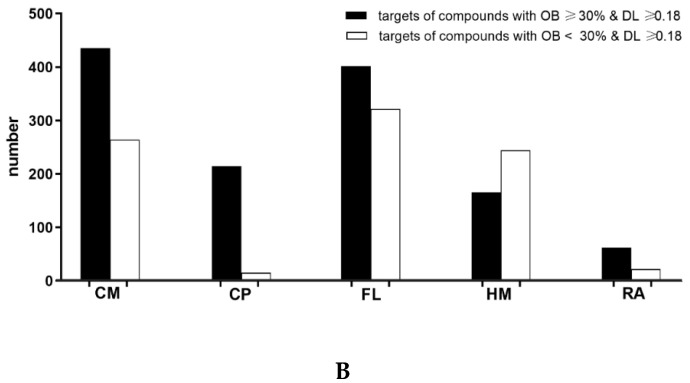
The counting of (**A**) compounds and (**B**) putative targets in the Pentaherb Formula. OB: oral bioavailability; DL: drug-likeness; CM: Cortex Moutan; CP: Cortex Phellodendri; FL: Flos Lonicerae; HM: Herba Menthae; RA: Rhizoma Atractylodis.

From the Traditional Chinese Medicine Systems Pharmacology (TCMSP) database, a total of 644 compounds were retrieved, namely, 164 in HM, 49 in RA, 55 in CM, 140 in CP, and 236 in FL. For active compound screening, drug-likeness (DL) ≥ 0.18 was adopted as DL is a qualitative notion used for estimating the drugability of a substance in drug design [23]. The compounds with higher oral bioavailability (OB) (OB ≥ 30%) and lower OB (OB < 30%) were screened for oral and topical use of PHF. The compounds and their putative targets of each herb in PHF are shown in Figure 1.

As a matter of fact, a total of 90 compounds including 10 (6.1%) compounds in HM, 9 (18.4%) compounds in RA, 11 (20%) compounds in CM, 37 (26.4%) compounds in CP, and 23 (9.7%) compounds in FL satisfied the criteria of OB ≥ 30% and DL ≥ 0.18. Therefore, 83 candidate compounds were then obtained after the removal of duplications (Supplementary Table S1). These compounds in PHF were associated with 1278 target proteins (165 in HM, 62 in RA, 435 in CM, 214 in CP, and 402 in FL) and 240 predicted targets were selected after eliminating the redundancy.

A total of 159 compounds including 23 (14.0%) compounds in HM, 11 (22.4%) compounds in RA, 33 (60.0%) compounds in CM, 25 (17.9%) compounds in CP, and 67 (28.4%) compounds in FL satisfied the criteria of OB < 30% and DL ≥ 0.18. Hence, 150 candidate compounds were obtained after the removal of duplications (Supplementary Table S2). These compounds in PHF were associated with 867 target proteins (244 in HM, 22 in RA, 264 in CM, 15 in CP, and 322 in FL) and 204 predicted targets were selected after eliminating the redundancy.

### 1.2. AD-Related Targets

After searching with the keyword “atopic dermatitis”, 1255 targets were acquired based on GeneCards database, and 12 targets were obtained based on Online Mendelian Inheritance in Man (OMIM) database. Finally, a total of 1256 known therapeutic targets for AD treatment were identified in this study after eliminating the redundancy (Supplementary Table S3).

### 1.3. Network Analysis

**Figure 2:**
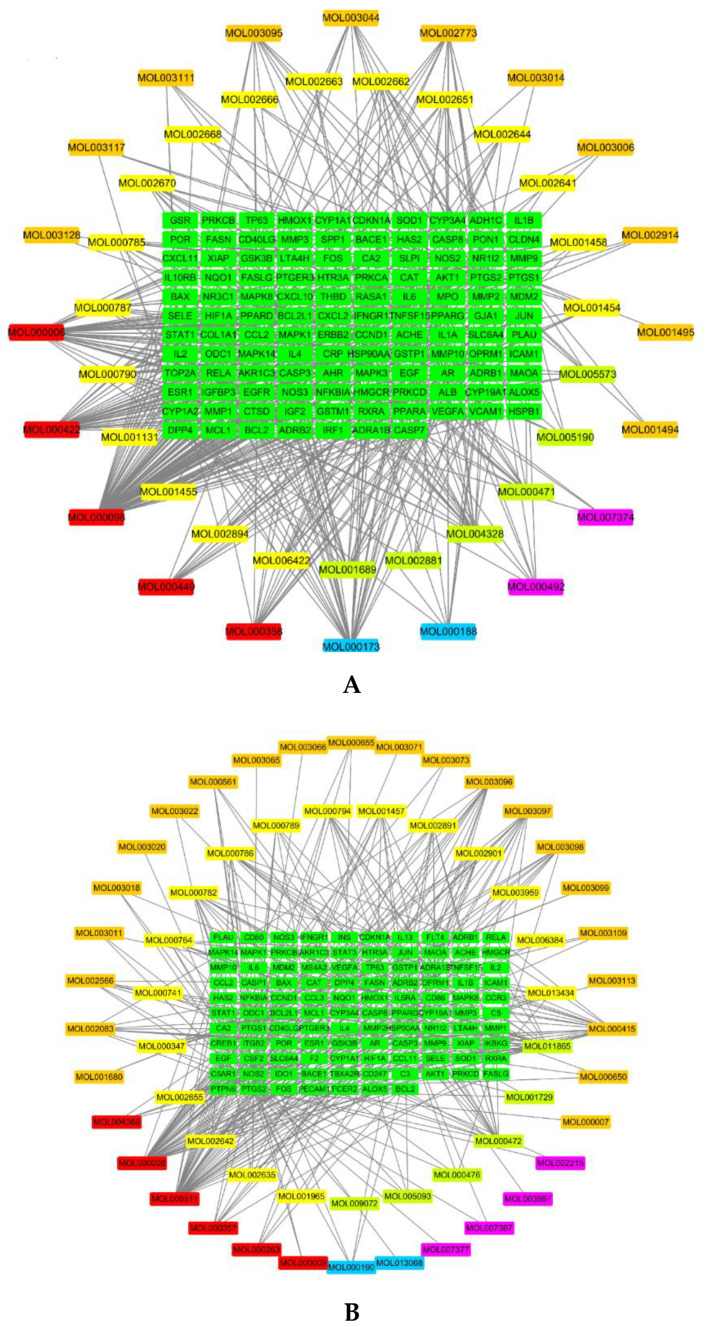
Compound–intersection targets network analysis. (**A**) Network of 43 compounds (OB ≥ 30% and DL ≥ 0.18) and intersection targets between compound and AD-related targets. (**B**) Network of 57 compounds (OB < 30% and DL ≥ 0.18) and intersection targets between compound and AD-related targets. The green rectangles represent PHF targets against AD; the Kelly green, yellow, orange, blue, and violet rectangles represent compounds from HM, CP, FL, RA, and CM, respectively. The red rectangles represent compounds from more than two herbs in PHF. PHF: Pentaherbs Formula; AD: atopic dermatitis; OB: oral bioavailability; DL: drug-likeness; CM: Cortex Moutan; CP: Cortex Phellodendri; FL: Flos Lonicerae; HM: Herba Menthae; RA: Rhizoma Atractylodis.

The compound–target network of the screened compounds and putative targets against AD was constructed. As shown in Figure 2A, there are 117 intersection targets between compound (OB ≥ 30% and DL ≥ 0.18) and AD-related targets. The intersection targets are derived from 43 of 83 active compounds in PHF. Most compounds affected multiple targets, and the key compounds acting on more than 34 targets are quercetin (MOL000098), which is from CM, FL, and CP, luteolin (MOL000006) which is from HM and FL, and kaempferol (MOL000422) which is from CM and FL (Table 1). As shown in Figure 2B, there are 107 intersection targets between compounds (OB < 30% and DL ≥ 0.18) and AD-related targets. The intersection targets are derived from 57 of 150 active compounds in PHF. Similarly, most active compounds are predicted to affect multiple targets. The key compounds acting on more than 24 targets are apigenin (MOL000008), which is from HM and FL, ursolic acid (MOL000511), which is from HM and FL, and rosmarinic acid (MOL011865), which is from HM (Table 2).

### 1.4. Protein–Protein Interaction and Network Analysis

**Figure 3:**
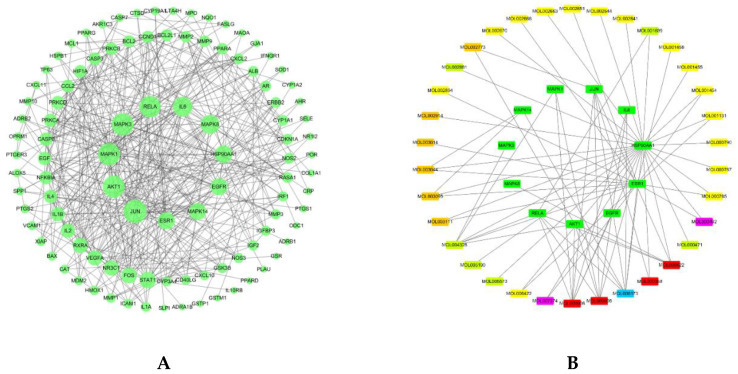

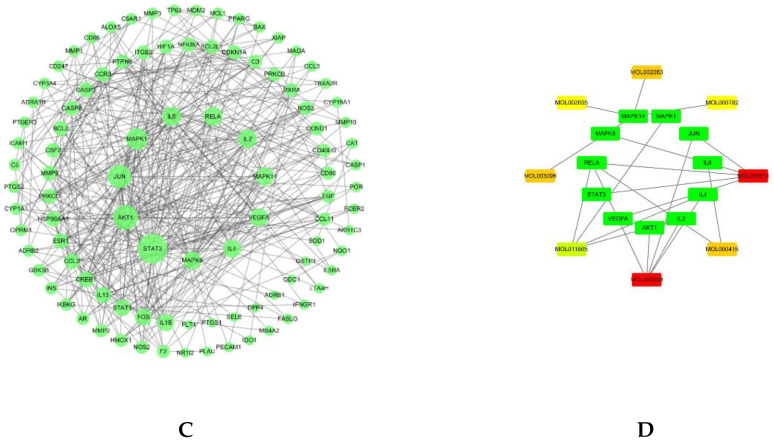
Protein–protein interaction (PPI) and network analysis. (**A**) PPI analysis of intersection targets between compound (OB ≥ 30% and DL ≥ 0.18) and AD-related targets, and (**B**) network analysis of screened core genes and related compounds. (**C**) PPI analysis of intersection targets between compound (OB < 30% and DL ≥ 0.18) and AD-related targets, and (**D**) network analysis of screened core genes and related compounds. The light gray circles represent intersection targets. The circles get larger with increasing degree. The Kelly green, yellow, orange, blue, and violet rectangles represent compounds from HM, CP, FL, RA, and CM, respectively. The red rectangles represent compounds from more than two herbs in PHF. The green rectangles represent core targets against AD. PHF: Pentaherbs Formula; AD: atopic dermatitis; OB: oral bioavailability; DL: drug-likeness; CM: Cortex Moutan; CP: Cortex Phellodendri; FL: Flos Lonicerae; HM: Herba Menthae; RA: Rhizoma Atractylodis.

The protein–protein interaction (PPI) analysis of the intersection targets was visualized by Cytoscape software and is shown in Figure 3. As shown in Figure 3A, the hub genes of compounds with OB ≥ 30% and DL ≥ 0.18 against AD are activator protein 1 (AP-1, also known as JUN, degree = 28), RAC-alpha serine/threonine-protein kinase (AKT1, degree = 26), mitogen-activated protein kinase 1 (MAPK1, degree = 26), MAPK3 (degree = 26), transcription factor p65 (RELA, degree = 25), IL-6 (degree = 22), MAPK8 (degree = 21), heat shock protein 90 alpha family class A member 1 (HSP90AA1, degree = 19), epidermal growth factor receptor (EGFR, degree = 18), MAPK14 (degree = 18), and estrogen receptor (ESR1, degree = 18). These hub genes were acted on by 34 compounds and the main compounds are quercetin, luteolin, and kaempferol (Figure 3B). As shown in Figure 3C, the hub genes of compounds with OB < 30% and DL ≥ 0.18 against AD are signal transducer and activator of transcription 3 (STAT3, degree = 39), AKT1 (degree = 30), JUN (degree = 27), MAPK1 (degree = 24), IL-6 (degree = 19), RELA (degree = 19), IL-2 (degree = 18), MAPK14 (degree = 17), vascular endothelial growth factor A (VEGFA, degree = 17), IL-4 (degree = 17), and MAPK8 (degree = 17). These hub genes were acted on by eight compounds, and the main compounds are apigenin, ursolic acid, and rosmarinic acid (Figure 3D).

### 1.5. Gene Ontology (GO) and Kyoto Encyclopedia of Genes and Genomes (KEGG) Enrichment Analysis

**Figure 4:**
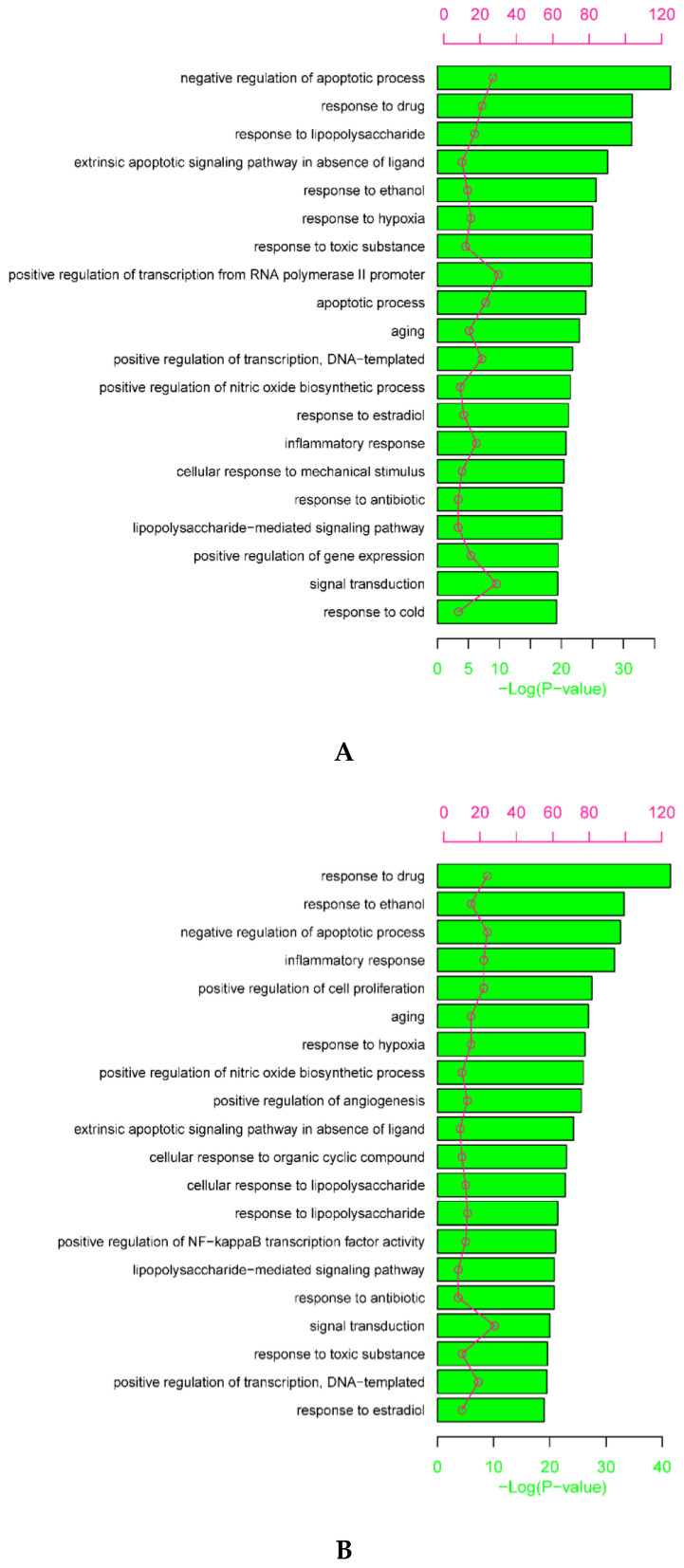
The top 20 significant gene ontology enrichment analysis for intersection targets between (**A**) compound (OB ≥ 30% and DL ≥ 0.18) and AD-related targets, and (**B**) compound (OB < 30% and DL ≥ 0.18) and AD-related targets. The circles represent the gene count of each biological process. AD: atopic dermatitis. OB: oral bioavailability; DL: drug-likeness.

**Figure 5:**
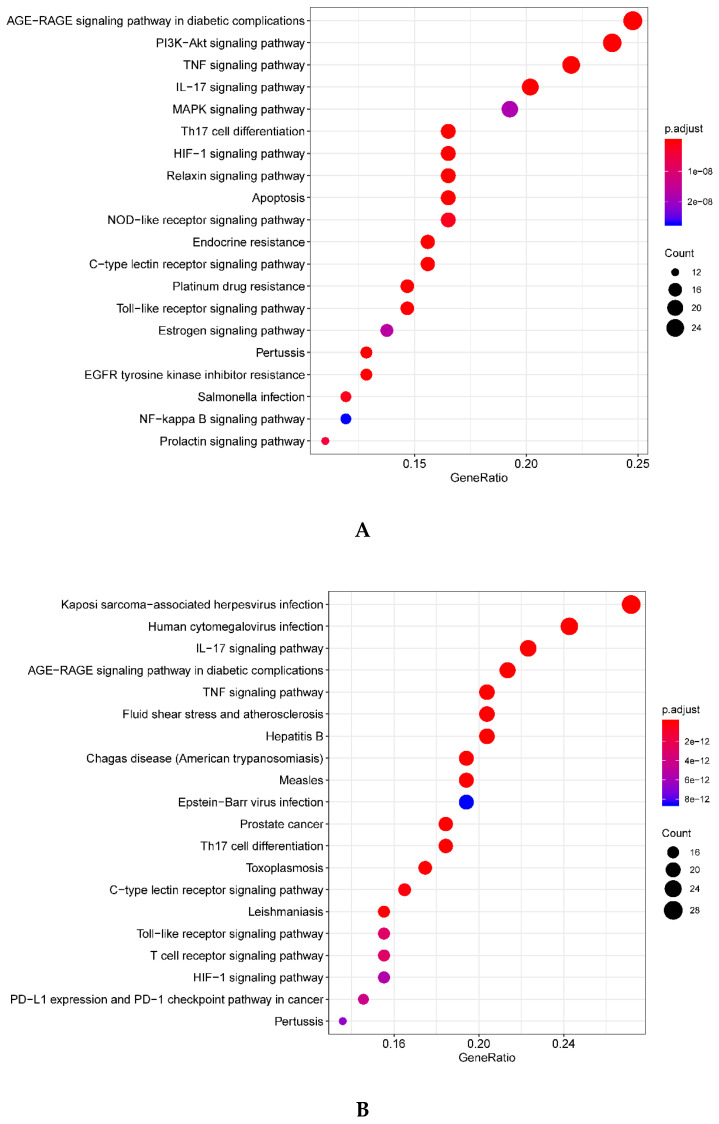
The top 20 significant enriched KEGG pathways for intersection targets between (**A**) compound (OB ≥ 30% and DL ≥ 0.18) and AD-related targets, and (**B**) compound (OB < 30% and DL ≥ 0.18) and AD-related targets. AD: atopic dermatitis. OB: oral bioavailability; DL: drug-likeness.

According to the GO enrichment analysis, the top 20 significant biological processes of intersection targets between compound (OB ≥ 30% and DL ≥ 0.18) and AD-related targets, and compound (OB < 30% and DL ≥ 0.18) targets and AD-related targets are shown in Figure 4 (adjusted *p* < 0.05). Importantly, the two different groups of intersection targets formed several similar biological processes involved in AD, including negative regulation of apoptotic process (GO: 0043066), inflammatory response (GO: 0006954), response to hypoxia (GO: 0071456), and positive regulation of nitric oxide biosynthetic process (GO: 0045428). The pathway analysis was performed to discover the underlying molecular mechanisms of PHF in AD treatment. The top 20 significant pathways of intersection targets between compound (OB ≥ 30% and DL ≥ 0.18), compound (OB < 30% and DL ≥ 0.18), and AD-related targets are shown in Figure 5 (adjusted *p* < 0.05). We also found the same signaling pathways in the different groups including TNF, IL-17, and hypoxia-inducible factor (HIF)-1 signaling pathway. The complete biological processes and signaling pathways formed by the intersection targets are listed in Supplementary Tables S4–S7.

### 1.6. Molecular Docking Analysis

**Figure 6:**
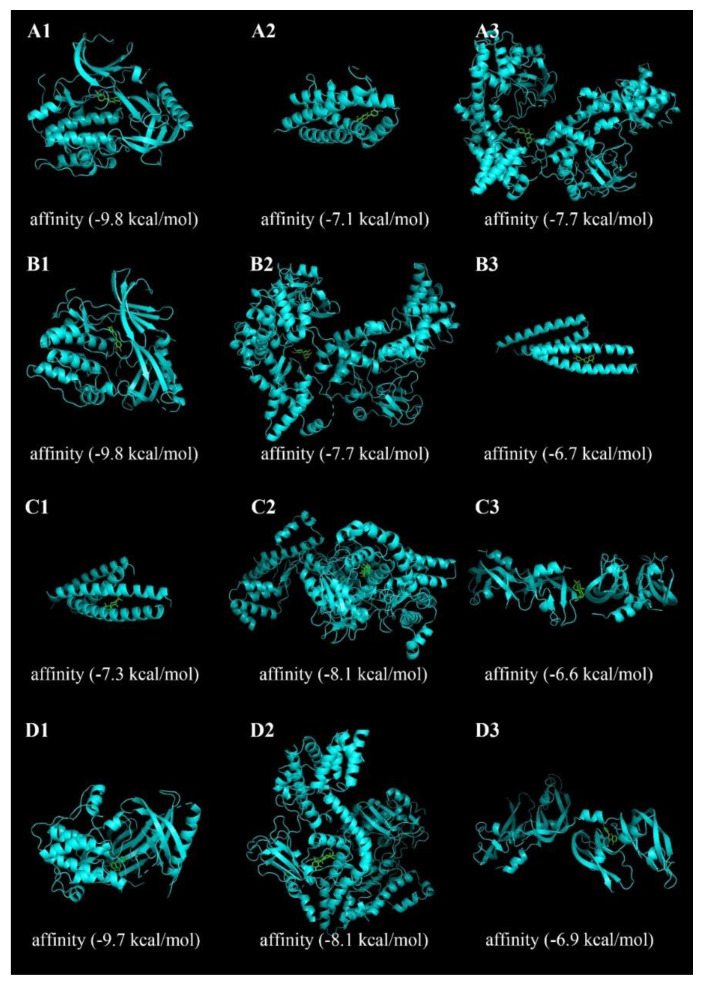
Molecular docking of compounds with core targets. (**A1**–**A3**) Docking process of quercetin with AKT1, IL-6, and RELA, respectively. (**B1**–**B3**) Docking process of luteolin with AKT1, REAL, and JUN, respectively. (**C1**–**C3**) Docking process of apigenin with JUN, RELA, and VEGFA, respectively. (**D1**–**D3**) Docking process of ursolic acid with AKT1, RELA, and VEGFA, respectively.

The docking structural models were collected from the lowest-energy docking solution of 20 docked conformations and are shown in Figure 6. The energy score represents the degree of docking coincidence of molecules. The lower the energy is, the better the binding of ligands to receptor proteins is [24]. Molecular docking results showed that PHF had good affinity for the binding of active ingredients to key AD target protein molecules. At the same time, these findings also indirectly verified that active ingredients had a regulatory effect on AD targets, such as RELA, IL-6, JUN, AKT1, and VEGFA. Moreover, the molecular docking results were consistent with the network pharmacology screening results, and the reliability of network pharmacology was verified by molecular docking in the present study.

## 2. Discussion

Based on the postulation of synergism, it has been a common clinical practice to combine multiple compatible herbs into a complex herbal formulation to improve therapeutic effects in China for thousands of years. In the theory of TCM syndrome (ZHENG in Chinese), “wind,” “dampness,” and “heat” are regarded as the major pathogenic factors for inflammatory skin diseases. The five herbal medicines in PHF are proposed to work as follows: clearing of the exterior wind-heat by FL and HM, clearing the heat from the blood by CM, and clearing of the interior heat by RA and CP. Each of the above herbal medicines have been extensively used in patients suffering from allergy, including asthma and AD, in China. Pharmacological studies documented that these herbs have antiallergic, anti-inflammatory, and sedative action for pruritus conditions, and they have been previously used in the study of childhood AD [17,19,25]. Different from the conventional “one drug–one target–one illness” research approach, the concept of network pharmacology aligns with the TCM theory and is therefore applicable for the investigation of multi-herbs and multitargets in complex TCM formulas in a holistic view [21].

In the present study, the active compounds and molecular mechanism of oral and topical application of PHF against AD were investigated based on OB and DL. The compounds with lower OB were considered to possibly participate in topical treatment, as we indeed found both oral and topical treatments of PHF are effective in treating AD [17,19]. In total, 43 compounds with OB ≥ 30% and 57 compounds with OB < 30% in PHF were selected after DL screening (Table 1 and Table 2). It was found that most of the compounds are multitargeted, hence the core targets against AD were screened (Figure 3). The key compounds acting on the core targets are quercetin, luteolin, and kaempferol among the compounds with higher OB; apigenin, ursolic acid, and rosmarinic acid among the compounds with lower OB. Quercetin, derived from three herbs CM, FL, and CP in PHF, is a potent pleiotropic polyphenol with antioxidant, anti-inflammatory, and antiallergic activities. It was shown that quercetin could inhibit mast cells from releasing histamine, and studies suggested it as a promising natural treatment for AD [26,27,28]. Importantly, preliminary human studies showed that no adverse effects were observed upon an oral intake of quercetin in doses up to one gram per day over three months, although higher doses up to 51.3 mg/kg body weight were associated with renal toxicity [29]. Luteolin, derived from HM and FL, exhibits anti-inflammatory activities by the activation of antioxidative enzymes, suppression of the NF-κB pathway and inhibition of proinflammatory substances, and antiallergic activities by inhibiting AP-1 activation in basophils [30,31]. It has also been reported that luteolin significantly inhibited the scratching behavior in skin-allergic mice [32]. The suppression role of kaempferol from CM and FL in immunoglobulin E (IgE)-mediated allergic responses has been proven, and kaempferol also exhibits anti-inflammatory activity in immune response [28,33]. The above three chemicals from four herbs in PHF mainly exerted the potential therapeutic action through both oral and topical treatments of AD because of their high OB. For apigenin, ursolic acid, and rosmarinic acid from FL and HM with lower OB, the potential therapeutic action through topical use was considered. Studies have reported potential therapeutic mechanisms of apigenin in cell cycle arrest, apoptosis, as well as anti-inflammatory and antioxidant actions. Dietary and topical use of apigenin attenuates the development of cutaneous inflammation in murine models [34,35]. Apigenin is safe because it does not cause any toxic effects, even at high doses [36]. Ursolic acid also exerts prominent antimicrobial, anticancer, anti-inflammatory, antioxidant, antidepressant, and anti-aging effects although their roles in AD are currently not clear [37,38,39]. The above results indicated that these six key compounds in PHF regulated most of the AD-related targets and all of them possess immunomodulatory properties. They may play crucial roles synergistically in the treatment of AD. Moreover, the screening of key compounds and herbal source can provide a novel strategy for optimizing the herbal ratio for different route of PHF administration.

For oral and topical treatment of AD by different compounds in PHF, similar biological processes and signaling pathways were found in the present study (Figure 4 and Figure 5). According to the result of enrichment analysis, several biological processes, including the negative regulation of apoptotic process, inflammatory response, response to hypoxia, and the positive regulation of nitric oxide biosynthetic process, were revealed for the treatment of AD with PHF (Figure 4). Cell renewal requires apoptosis; however, excessive cytokines and chemokines induced by skin infection often leads to extensive apoptosis of keratinocytes and thus causes eczema and spongiosis in AD patients [40]. PHF is involved in the negative regulation of apoptotic process in AD, which may benefit for keratinocyte renewal. In the present study, cellular response to hypoxia was also found to be involved in the PHF treatment for AD because the terminal differentiation of keratinocytes and the formation of the epidermal barrier can be impaired by low O_2_ tension [41]. Nitric oxide (NO), a promising topical antimicrobial agent with broad spectrum and less resistance potential in skin and soft tissue infection, can diffuse freely through the plasma membrane of a cell [42]. Positive regulation of NO biosynthetic process by PHF herbs was found in the present study. Moreover, positive regulation of blood vessel endothelial cell migration, angiogenesis, and wound healing were found to play crucial roles in the treatment of AD. AD is the most ubiquitous inflammatory skin disorder, and the involvement of multiple signaling pathways could contribute to the pathogenesis of AD simultaneously. The KEGG pathway analysis revealed that PHF acts on several signaling pathways, including TNF signaling, IL-17 signaling, and HIF-1 signaling in the treatment of AD (Figure 5). As TNF-TNFR2 interaction contributes to the exacerbation of AD, targeting TNF-TNFR2 in AD is a promising therapy [43]. HIF hydroxylases and HIF pathway have been reported in AD, and pharmacologic inhibition of HIF hydroxylases has been suggested to be a novel therapeutic approach in treating allergic contact dermatitis [44]. Although AD is a Th2-dominated disease with overexpression of IL-4, IL-5, and IL-13, the dysregulation of IL-17/Th17 in AD was reported in several studies [45,46]. Besides, advanced glycation end products-receptor for advanced glycation end products (AGE-RAGE) signaling pathways involved in diabetic complications were also found by enrichment analysis as skin injury has been reported in diabetes mellitus; however, the role of AGE-RAGE signaling pathways in AD needs further experimental validations [47]. The PPI analysis revealed several critical genes against AD including AKT1, RELA, JUN, IL-6 and so on. It has been reported that the decreased expression and activity of AKT1 in the skin will lead to the change of protease expression, the decrease in filaggrin expression, and finally the destruction of the skin barrier [48]. Nucleic acid drug that targeting RELA (p65 NF-κB) gene has been studied in clinical trials in inflammatory diseases including AD [49] (www.anges.co.jp, AnGes, Japan). It also has been reported that activation of JUN (AP-1) may cause increased expression of IL-31 in AD skin [50]. Studies reported that IL-6 and IL-6 receptor signaling was also one of the risk factors in AD and blocking of IL-6R could alleviate AD [51,52]. Further investigation should be performed to verify the mechanism of these core targets and the signaling pathway exerted by PHF herbs.

## 3. Methods

### 3.1. Information of PHF Active Compounds, Compound Targets and AD-Related Targets

The chemical ingredients of the five herbs contained in PHF were obtained by the TCMSP database (TCMSP, Version: 2.3, http://lsp.nwu.edu.cn/tcmsp.php) with the absorption, distribution, metabolism, and excretion (ADME) system predicting the OB and DL [53]. The PHF compound and compound target interactions were determined by the DrugBank database (https://www.drugbank.ca/) [54]. AD-related targets were gathered from the GeneCards database (https://www.genecards.org/, version 4.12) [55] and the OMIM database (http://www.omim.org/, updated on 21 March 2020) [56].

### 3.2. Network Construction

The networks of compounds, compound targets and AD-related targets were merged using Cytoscape software (version 3.7.2, Boston, MA, USA) [57] to visualize the association between the active chemicals and AD-related targets.

### 3.3. Protein–Protein Interaction Analysis

The data of PPI came from the Search Tool for the Retrieval of Interacting Genes/Proteins (STRING) (http://stringdb.org/, ver. 10) with the species limited to “Homo sapiens” and a confidence score > 0.9 [58]. STRING is a functional protein association database for forecasting protein–protein interactions. It defines PPI as confidence ranges (low confidence: scores > 0.15; medium: >0.4; high: >0.7; highest: >0.9). The PPI results were visualized using Cytoscape software to obtain the core targets. The core targets and compounds, and herbal sources, were also visualized using Cytoscape software.

### 3.4. Gene Ontology and KEGG Enrichment Analysis

The GO of the biological process (BP) was analyzed to further validate the relation between the potential targets and AD. Kyoto Encyclopedia of Genes and Genomes (KEGG) enrichment analysis was adopted to predict the potential signaling pathway of PHF in AD. The GO and KEGG enrichment analysis was performed by the Database for Annotation, Visualization and Integrated Discovery (DAIVD) Bioinformatics Resources 6.7 (https://david-d.ncifcrf.gov/), which provide functional interpretations of large lists of genes derived from genomic studies [59]. The statistical significance was calculated using the classical hypergeometric test. After using the Benjamini–Hochberg method to control the false discovery rate (FDR) for multiple hypothesis tests, the adjusted *p* value < 0.05 was used as the significant cutoff in our study.

### 3.5. Docking Steps and Results Evaluation

Autodock software (AutoDock 4.2, San Carlos, CA, USA) was used to perform protein compound docking analysis [24]. Briefly, two-dimensional (2D) structures of the compounds were obtained from NCBI PubChem structure files, and 3D structures were built using ChemBio 3D Ultra software (CambridgeSoft, version 14.0) after energy minimization with MM2. The crystal structures of the proteins were obtained from the Protein Data Bank (https://www.rcsb.org/) [60]. The ligands within the crystal structure complex were extracted by PyMOL software (San Carlos, CA, USA). AutoDock software was used to add polar hydrogen to the entire receptor, and the grid box was set to contain the entire receptor region. For all the docking studies, 20 docked conformations were generated for each pair of ligand and receptor, and the energy calculations were performed employing the genetic algorithms. A binding energy of less than zero indicates that a ligand can spontaneously bind to the receptor. It is generally accepted that when the conformation of ligand and receptor binding is stable, the lower the energy score is, the more likely the binding is to occur [24].

## 4. Conclusions

The existing treatment of AD still cannot satisfy the needs in clinics, and the side effects caused by the prolonged use of Western medicine cannot be neglected. As an important health resource in China, TCM with multitarget and multicomponent properties can exert therapeutic effects on incurable diseases in a systematic and holistic manner. Our study has successfully revealed the potential biological processes and signaling pathways of PHF against AD and uncovered the rationality of herbal combinations. These results can shed light on the therapeutic mechanism of herbal medicine for the oral and topical treatment of AD and facilitate TCM drug discovery. We believe the rational use of Chinese herbal medicine can provide convenient, high-quality, and inexpensive basic health services for community residents.

## Figures and Tables

**Table 1:** The screening of active compounds in PHF for oral treatment of AD.

	MOL ID	MOL Name	OB (%)	DL	Herb Source	Targets (No.)
**1**	MOL000098	quercetin	46.43	0.28	CM, FL, CP	84
**2**	MOL000006	luteolin	36.16	0.25	HM, FL	37
**3**	MOL000422	kaempferol	41.88	0.24	CM, FL	34
**4**	MOL000173	wogonin	30.68	0.23	RA	28
**5**	MOL004328	naringenin	59.29	0.21	HM	19
**6**	MOL001689	acacetin	34.97	0.24	HM	17
**7**	MOL002773	beta-carotene	37.18	0.58	FL	16
**8**	MOL000358	beta-sitosterol	36.91	0.75	CP, FL	14
**9**	MOL000471	aloe-emodin	83.38	0.24	HM	12
**10**	MOL003095	5-hydroxy-7-methoxy-2-(3,4,5-trimethoxyphenyl)chromone	51.96	0.41	FL	12
**11**	MOL002662	rutaecarpine	40.3	0.6	CP	11
**12**	MOL000449	Stigmasterol	43.83	0.76	CP, FL	10
**13**	MOL002670	Cavidine	35.64	0.81	CP	10
**14**	MOL000790	Isocorypalmine	35.77	0.59	CP	10
**15**	MOL003044	Chryseriol	35.85	0.27	FL	10
**16**	MOL001455	(S)-Canadine	53.83	0.77	CP	9
**17**	MOL000188	3β-acetoxyatractylone	40.57	0.22	RA	8
**18**	MOL001454	berberine	36.86	0.78	CP	8
**19**	MOL002651	Dehydrotanshinone II A	43.76	0.4	CP	8
**20**	MOL000785	palmatine	64.6	0.65	CP	8
**21**	MOL000787	Fumarine	59.26	0.83	CP	8
**22**	MOL005573	Genkwanin	37.13	0.24	HM	7
**23**	MOL000492	(+)-catechin	54.83	0.24	CM	7
**24**	MOL006422	thalifendine	44.41	0.73	CP	7
**25**	MOL002644	Phellopterin	40.19	0.28	CP	6
**26**	MOL002894	berberrubine	35.74	0.73	CP	6
**27**	MOL002881	Diosmetin	31.14	0.27	HM	5
**28**	MOL005190	eriodictyol	71.79	0.24	HM	5
**29**	MOL001458	coptisine	30.67	0.86	CP	5
**30**	MOL003111	Centauroside_qt	55.79	0.5	FL	5
**31**	MOL002668	Worenine	45.83	0.87	CP	4
**32**	MOL001131	phellamurin_qt	56.6	0.39	CP	4
**33**	MOL007374	5-[[5-(4-methoxyphenyl)-2-furyl]methylene]barbituric acid	43.44	0.3	CM	3
**34**	MOL002666	Chelerythrine	34.18	0.78	CP	3
**35**	MOL002914	Eriodyctiol (flavanone)	41.35	0.24	FL	3
**36**	MOL003006	(-)-(3R,8S,9R,9aS,10aS)-9-ethenyl-8-(beta-D-glucopyranosyloxy)-2,3,9,9a,10,10a-hexahydro-5-oxo-5H,8H-pyrano[4,3-d]oxazolo[3,2-a]pyridine-3-carboxylic acid_qt	87.47	0.23	FL	3
**37**	MOL003117	Ioniceracetalides B_qt	61.19	0.19	FL	3
**38**	MOL002641	Phellavin_qt	35.86	0.44	CP	2
**39**	MOL002663	Skimmianin	40.14	0.2	CP	2
**40**	MOL001494	Mandenol	42	0.19	FL	2
**41**	MOL003014	secologanic dibutylacetal_qt	53.65	0.29	FL	2
**42**	MOL003128	dinethylsecologanoside	48.46	0.48	FL	2
**43**	MOL001495	Ethyl linolenate	46.1	0.2	FL	1

PHF: Pentaherbs Formula; AD: atopic dermatitis. OB: oral bioavailability; DL: drug-likeness; CM: Cortex Moutan; CP: Cortex Phellodendri; FL: Flos Lonicerae; HM: Herba Menthae; RA: Rhizoma Atractylodis.

**Table 2:** The screening of active compounds in PHF for topical treatment of AD.

	MOL ID	MOL Name	OB (%)	DL	Herb Source	Targets (No.)
**1**	MOL000008	apigenin	23.06	0.21	HM, FL	40
**2**	MOL000511	ursolic acid	16.77	0.75	HM, FL	38
**3**	MOL011865	rosmarinic acid	1.38	0.35	HM	24
**4**	MOL000415	rutin	3.20	0.68	FL	18
**5**	MOL000472	emodin	24.4	0.24	HM	16
**6**	MOL000782	Menisporphine	24.33	0.52	CP	12
**7**	MOL000786	STOCK1N-14407	22.28	0.64	CP	11
**8**	MOL002891	magnoflorine	0.48	0.55	CP	11
**9**	MOL003096	2-(3,4-dimethoxyphenyl)-5-hydroxy-7-methoxy-chromone	29.24	0.34	FL	11
**10**	MOL003097	Flavone der.	27.12	0.27	FL	11
**11**	MOL000794	menisperine	26.17	0.59	CP	10
**12**	MOL002083	tricin	27.86	0.34	FL	10
**13**	MOL000764	magnoflorine	26.69	0.55	CP	9
**14**	MOL001457	columbamine	26.94	0.59	CP	9
**15**	MOL002642	phellodendrine	2.61	0.58	CP	8
**16**	MOL002655	Amurensin_qt	26.37	0.44	CP	8
**17**	MOL003098	2-(2,4-dimethoxyphenyl)-3-hydroxy-7-methoxy-chromone	12.94	0.33	FL	8
**18**	MOL000357	Sitogluside	20.63	0.62	CP, HM, FL	7
**19**	MOL000789	jatrorrizine	19.65	0.59	CP	7
**20**	MOL002566	3-O-Methylquercetin	10.1	0.3	FL	7
**21**	MOL002635	(±)-lyoniresinol	4.87	0.54	CP	7
**22**	MOL002901	phellodendrine	2.5	0.58	CP	7
**23**	MOL000347	Syrigin	14.64	0.32	CP	6
**24**	MOL013434	Auraptene	25.62	0.24	CP	6
**25**	MOL000561	Astragalin	14.03	0.74	FL	5
**26**	MOL000190	3,5-dimethoxy-4-glucosyloxyphenylallylalcohol	29	0.32	RA	4
**27**	MOL000263	oleanolic acid	29.02	0.76	CM, HM, FL	4
**28**	MOL000476	Physcion	22.29	0.27	HM	4
**29**	MOL004368	Hyperin	6.94	0.77	CP, FL	4
**30**	MOL005093	Diosmin	12.70	0.66	HM	4
**31**	MOL000650	1H,3H-Pyrano(3,4-c)pyran-1-one, 5-ethenyl-6-(beta-D-glucopyranosyloxy)-4,4a,5,6-tetrahydro-, (4aS-(4aalpha,5beta,6alpha))-	4.96	0.38	FL	3
**32**	MOL000655	Loganic acid	4.92	0.4	FL	3
**33**	MOL000741	(2S,3S)-3,5,7-trihydroxy-2-(4-hydroxyphenyl)chroman-4-one	24.15	0.24	CP	3
**34**	MOL001729	Crysophanol	18.64	0.21	HM	3
**35**	MOL001965	Dauricine (8CI)	23.65	0.37	CP	3
**36**	MOL003011	Secologanate	17.56	0.33	FL	3
**37**	MOL003018	SCG	23.59	0.36	FL	3
**38**	MOL003109	Caeruloside C_qt	5.4	0.37	FL	3
**39**	MOL000009	luteolin-7-o-glucoside	7.29	0.78	HM, FL	2
**40**	MOL001680	Loganin	5.9	0.44	FL	2
**41**	MOL002215	Oleanic acid	8.41	0.77	CM	2
**42**	MOL003022	Secoxyloganin	3.79	0.39	FL	2
**43**	MOL003073	8-epiloganin	11.68	0.44	FL	2
**44**	MOL003099	7-epi-Loganin	4.78	0.44	FL	2
**45**	MOL007367	paeonoside	18.52	0.24	CM	2
**46**	MOL000007	Cosmetin	9.68	0.74	FL	1
**47**	MOL003020	secologanoside 7-methylester	3.88	0.45	FL	1
**48**	MOL003065	4-caffeoylquinic acid	10.48	0.33	FL	1
**49**	MOL003066	Neochlorogenic acid	10.65	0.33	FL	1
**50**	MOL003071	secologanoside	26.92	0.37	FL	1
**51**	MOL003113	Dehydroxymorroniside	20.69	0.46	FL	1
**52**	MOL003867	Paeonolide	6.3	0.64	CM	1
**53**	MOL003959	limonin	21.3	0.57	CP	1
**54**	MOL006384	4-[(1R,3aS,4R,6aS)-4-(4-hydroxy-3,5-dimethoxyphenyl)-1,3,3a,4,6,6a-hexahydrofuro[4,3-c]furan-1-yl]-2,6-dimethoxyphenol	3.29	0.72	CP	1
**55**	MOL007377	mudanoside A	13.39	0.29	CM	1
**56**	MOL009072	Prunin	9.33	0.74	HM	1
**57**	MOL013068	Oroxindin	7.07	0.77	RA	1

PHF: Pentaherbs Formula; AD: atopic dermatitis. OB: Oral bioavailability; DL: drug-likeness; CM: Cortex Moutan; CP: Cortex Phellodendri; FL: Flos Lonicerae; HM: Herba Menthae; RA: Rhizoma Atractylodis.

## Supplementary Materials

The following are available online at https://www.mdpi.com/2223-7747/9/9/1166/s1, Table S1: Detailed information of active compounds (OB ≥ 30% and DL ≥ 0.18) in PHF; Table S2: Detailed information of active compounds (OB < 30% and DL ≥ 0.18) in PHF; Table S3: Detailed information of human disease targets of atopic dermatitis; Table S4: The gene ontology enrichment analysis for intersection targets between compound (OB ≥ 30% and DL ≥ 0.18) and AD related targets; Table S5: The gene ontology enrichment analysis for intersection targets between compound (OB < 30% and DL ≥ 0.18) and AD related targets; Table S6: The enriched KEGG pathways for intersection targets between compound (OB ≥ 30% and DL ≥ 0.18) and AD related targets; Table S7: The enriched KEGG pathways for intersection targets between compound (OB < 30% and DL ≥ 0.18) and AD related targets.

## Abbreviations

AD	Atopic dermatitis
Th	T helper
TCM	Traditional Chinese Medicine
PHF	pentaherbs formula
CM	Cortex Moutan
CP	Cortex Phellodendri
FL	Flos Lonicerae
HM	Herba Menthae
RA	Rhizoma Atractylodis
TCMSP	Traditional Chinese Medicine Systems Pharmacology Database
ADME	The absorption, distribution, metabolism and excretion
OB	oral bioavailability
DL	drug-likeness
OMIM	Online Mendelian Inheritance in Man
PPI	protein–protein interaction
GO	gene ontology
BP	biological process
KEGG	Kyoto Encyclopedia of Genes and Genomes
FDR	false discovery rate
AP-1	activator protein 1
AKT1	RAC-alpha serine/threonine-protein kinase
MAPK1	mitogen-activated protein kinase 1
MAPK3	mitogen-activated protein kinase 3
RELA	transcription factor p65
IL-6	interleukin-6
MAPK8	mitogen-activated protein kinase 8
HSP90AA1	heat shock protein 90 alpha family class A member 1
EGFR	epidermal growth factor receptor
MAPK14	mitogen-activated protein kinase 14
ESR1	estrogen receptor
STAT3	signal transducer and activator of transcription 3
VEGFA	vascular endothelial growth factor A
HIF	hypoxia-inducible factor
IgE	immunoglobulin E
CUHK	The Chinese University of Hong Kong
DAVID	Database for Annotation, Visualization and Integrated Discovery
STRING	Search Tool for the Retrieval of Interacting Genes/Proteins
AGE-RAGE	advanced glycation end products-receptor for advanced glycation end products
