# Supplementary material for: The Active Compounds and Therapeutic Mechanisms of Pentaherbs Formula for Oral and Topical Treatment of Atopic Dermatitis Based on Network Pharmacology
Source: Plants (Basel). 2020 Sep 9;9(9):1166. doi: 10.3390/plants9091166 (PMC7569866; doi:10.3390/plants9091166)
Supplement: Supplementary file 1 [file plants-09-01166-s001.pdf]

Table S1. Detailed information of active compounds (OB ⩾ 30% and DL ⩾ 0.18) in PHF

	MOL ID	MOL Name	OB	DL	Herb source
**1**	MOL011616	Fortunellin	35.65	0.74	BH
**2**	MOL001689	acacetin	34.97	0.24	BH
**3**	MOL001790	Linarin	39.84	0.71	BH
**4**	MOL002881	Diosmetin	31.14	0.27	BH
**5**	MOL000359	sitosterol	36.91	0.75	BH、DP
**6**	MOL004328	naringenin	59.29	0.21	BH
**7**	MOL000471	aloe-emodin	83.38	0.24	BH
**8**	MOL005190	eriodictyol	71.79	0.24	BH
**9**	MOL005573	Genkwanin	37.13	0.24	BH
**10**	MOL000006	luteolin	36.16	0.25	BH、JYH
**11**	MOL000173	wogonin	30.68	0.23	CZ
**12**	MOL000179	2-Hydroxyisoxypropyl-3-hydroxy-7-isopentene-2,3-dihydrobenzofuran-5-carboxylic	45.2	0.2	CZ
**13**	MOL000184	NSC63551	39.25	0.76	CZ
**14**	MOL000186	Stigmasterol 3-O-beta-D-glucopyranoside_qt	43.83	0.76	CZ
**15**	MOL000188	3β-acetoxyatractylone	40.57	0.22	CZ
**16**	MOL000085	beta-daucosterol_qt	36.91	0.75	CZ
**17**	MOL000088	beta-sitosterol 3-O-glucoside_qt	36.91	0.75	CZ
**18**	MOL000092	daucosterin_qt	36.91	0.76	CZ
**19**	MOL000094	daucosterol_qt	36.91	0.76	CZ
**20**	MOL001925	paeoniflorin_qt	68.18	0.4	DP
**21**	MOL000211	Mairin	55.38	0.78	DP
**22**	MOL000422	kaempferol	41.88	0.24	DP、JYH
**23**	MOL000492	(+)-catechin	54.83	0.24	DP
**24**	MOL007003	benzoyl paeoniflorin	31.14	0.54	DP
**25**	MOL007369	4-O-methylpaeoniflorin_qt	67.24	0.43	DP
**26**	MOL007374	5-[[5-(4-methoxyphenyl)-2-furyl]methylene]barbituric acid	43.44	0.3	DP
**27**	MOL007382	mudanpioside-h_qt 2	42.36	0.37	DP
**28**	MOL007384	paeonidanin_qt	65.31	0.35	DP
**29**	MOL000098	quercetin	46.43	0.28	DP、JYH、HB
**30**	MOL001454	berberine	36.86	0.78	HB
**31**	MOL001458	coptisine	30.67	0.86	HB
**32**	MOL002636	Kihadalactone A	34.21	0.82	HB
**33**	MOL013352	Obacunone	43.29	0.77	HB
**34**	MOL002641	Phellavin_qt	35.86	0.44	HB
**35**	MOL002643	delta 7-stigmastenol	37.42	0.75	HB
**36**	MOL002644	Phellopterin	40.19	0.28	HB
**37**	MOL002651	Dehydrotanshinone II A	43.76	0.4	HB
**38**	MOL002652	delta7-Dehydrosophoramine	54.45	0.25	HB
**39**	MOL002656	dihydroniloticin	36.43	0.81	HB
**40**	MOL002659	kihadanin A	31.6	0.7	HB
**41**	MOL002660	niloticin	41.41	0.82	HB
**42**	MOL002662	rutaecarpine	40.3	0.6	HB
**43**	MOL002663	Skimmianin	40.14	0.2	HB
**44**	MOL002666	Chelerythrine	34.18	0.78	HB
**45**	MOL000449	Stigmasterol	43.83	0.76	HB、JYH
**46**	MOL002668	Worenine	45.83	0.87	HB

**47**	MOL002670	Cavidine	35.64	0.81	HB
**48**	MOL002671	Candletoxin A	31.81	0.69	HB
**49**	MOL002672	Hericenone H	39	0.63	HB
**50**	MOL002673	Hispidone	36.18	0.83	HB
**51**	MOL000358	beta-sitosterol	36.91	0.75	HB、JYH
**52**	MOL000622	Magnograndiolide	63.71	0.19	HB
**53**	MOL000762	Palmidin A	35.36	0.65	HB
**54**	MOL000785	palmatine	64.6	0.65	HB
**55**	MOL000787	Fumarine	59.26	0.83	HB
**56**	MOL000790	Isocorypalmine	35.77	0.59	HB
**57**	MOL001131	phellamurin_qt	56.6	0.39	HB
**58**	MOL001455	(S)-Canadine	53.83	0.77	HB
**59**	MOL001771	poriferast-5-en-3beta-ol	36.91	0.75	HB
**60**	MOL002894	berberrubine	35.74	0.73	HB
**61**	MOL005438	campesterol	37.58	0.71	HB
**62**	MOL006392	dihydroniloticin	36.43	0.82	HB
**63**	MOL006401	melianone	40.53	0.78	HB
**64**	MOL006413	phellochin	35.41	0.82	HB
**65**	MOL006422	thalifendine	44.41	0.73	HB
**66**	MOL001494	Mandenol	42	0.19	JYH
**67**	MOL001495	Ethyl linolenate	46.1	0.2	JYH
**68**	MOL002707	phytofluene	43.18	0.5	JYH
**69**	MOL002914	Eriodyctiol (flavanone)	41.35	0.24	JYH
**70**	MOL003006	(-)-(3R,8S,9R,9aS,10aS)-9-ethenyl-8-(beta-D-glucopyranosyloxy)-2,3,9,9a,10,10a-hexahydro-5-oxo-5H,8H-pyrano[4,3-d]oxazolo[3,2-a]pyridine-3-carboxylic acid qt	87.47	0.23	JYH
**71**	MOL003014	secologanic dibutylacetal_qt	53.65	0.29	JYH
**72**	MOL002773	beta-carotene	37.18	0.58	JYH
**73**	MOL003036	ZINC03978781	43.83	0.76	JYH
**74**	MOL003044	Chryseriol	35.85	0.27	JYH
**75**	MOL003059	kryptoxanthin	47.25	0.57	JYH
**76**	MOL003062	4,5'-Retro-.beta.,.beta.-Carotene-3,3'-dione, 4',5'-didehydro-	31.22	0.55	JYH
**77**	MOL003095	5-hydroxy-7-methoxy-2-(3,4,5-trimethoxyphenyl)chromone	51.96	0.41	JYH
**78**	MOL003101	7-epi-Vogeloside	46.13	0.58	JYH
**79**	MOL003108	Caeruloside C	55.64	0.73	JYH
**80**	MOL003111	Centauroside_qt	55.79	0.5	JYH
**81**	MOL003117	Ioniceracetalides B_qt	61.19	0.19	JYH
**82**	MOL003124	XYLOSTOSIDINE	43.17	0.64	JYH
**83**	MOL003128	dinethylsecologanoside	48.46	0.48	JYH

PHF: Pentaherbs Formula; AD: atopic dermatitis. OB: oral bioavailability; DL: drug-likeness; CM:

Table S2. Detailed information of active compounds (OB < 30% and DL ⩾ 0.18) in PHF

	MOL ID	MOL Name	OB	DL	Herb source
**1**	MOL000007	Cosmetin	9.68	0.74	FL
**2**	MOL000008	apigenin	23.06	0.21	HM、FL
**3**	MOL000009	luteolin-7-o-glucoside	7.29	0.78	HM、FL
**4**	MOL000010	Rhoifolin	6.68	0.77	FL
**5**	MOL000084	beta-daucosterol	20.63	0.63	RA
**6**	MOL000086	(24S)-5beta-Stigmastan-3beta-ol	25.32	0.75	RA
**7**	MOL000087	beta-sitosterol 3-O-glucoside	20.63	0.62	RA
**8**	MOL000091	daucosterin	20.63	0.63	RA
**9**	MOL000093	daucosterol	20.63	0.63	RA
**10**	MOL000095	delta 7-stigmastenol	25.32	0.75	RA、CM
**11**	MOL000182	Atractyloyne	5.2	0.23	RA
**12**	MOL000185	Stigmasterol 3-O-beta-D-glucopyrano	21.32	0.63	RA
**13**	MOL000190	3,5-dimethoxy-4-glucosyloxyphenylal	29	0.32	RA
**14**	MOL000192	2-(1,4a-dimethyl-2,3-dihydroxydecah	5.96	0.47	RA
**15**	MOL000263	oleanolic acid	29.02	0.76	CM、HM、FL
**16**	MOL000347	Syrigin	14.64	0.32	CP
**17**	MOL000357	Sitogluside	20.63	0.62	CP、HM、FL
**18**	MOL000415	rutin	3.2	0.68	FL
**19**	MOL000472	emodin	24.4	0.24	HM
**20**	MOL000476	Physcion	22.29	0.27	HM
**21**	MOL000508	Friedelin	29.16	0.76	CP
**22**	MOL000511	ursolic acid	16.77	0.75	HM、FL
**23**	MOL000551	Hederagenol	22.42	0.74	FL
**24**	MOL000561	Astragalin	14.03	0.74	FL
**25**	MOL000650	1H,3H-Pyrano(3,4-c)pyran-1-one, 5-e	4.96	0.38	FL
**26**	MOL000655	Loganic acid	4.92	0.4	FL
**27**	MOL000663	lignoceric acid	14.9	0.33	FL
**28**	MOL000741	(2S,3S)-3,5,7-trihydroxy-2-(4-hydrox	24.15	0.24	CP
**29**	MOL000764	magnoflorine	26.69	0.55	CP
**30**	MOL000782	Menisporphine	24.33	0.52	CP
**31**	MOL000786	STOCK1N-14407	22.28	0.64	CP
**32**	MOL000789	jatrorrizine	19.65	0.59	CP
**33**	MOL000794	menisperine	26.17	0.59	CP
**34**	MOL001410	Zeaxanthin	21.17	0.54	FL
**35**	MOL001457	columbamine	26.94	0.59	CP
**36**	MOL001680	Loganin	5.9	0.44	FL
**37**	MOL001729	Crysophanol	18.64	0.21	HM
**38**	MOL001875	isochlorogenic,acid	1.79	0.69	FL
**39**	MOL001915	paeonoside	3.47	0.71	CM
**40**	MOL001955	Heriguard	11.93	0.33	FL
**41**	MOL001965	Dauricine (8CI)	23.65	0.37	CP
**42**	MOL001982	disacoside B_qt	15.83	0.74	FL
**43**	MOL002083	tricin	27.86	0.34	FL
**44**	MOL002215	Oleanic acid	8.41	0.77	CM
**45**	MOL002229	HEPTACOSANE	8.18	0.36	FL
**46**	MOL002329	Javanicin	17.38	0.78	CP
**47**	MOL002566	3-O-Methylquercetin	10.1	0.3	FL
**48**	MOL002635	(±)-lyoniresinol	4.87	0.54	CP
**49**	MOL002637	Obacunoic acid	20.69	0.79	CP
**50**	MOL002640	phellavin	3.9	0.83	CP

**51**	MOL002642	phellodendrine	2.61	0.58	CP
**52**	MOL002646	Vanilloloside	8.58	0.21	CP
**53**	MOL002649	Coniferin	10.28	0.27	CP
**54**	MOL002654	Amurensin	3.74	0.83	CP
**55**	MOL002655	Amurensin_qt	26.37	0.44	CP
**56**	MOL002657	hispidol B	19.72	0.81	CP
**57**	MOL002658	kihadalactone B	16.71	0.79	CP
**58**	MOL002661	nomilin	17.77	0.67	CP
**59**	MOL002669	Campesteryl ferulate	22.1	0.59	CP
**60**	MOL002702	Nonacosanol	10.57	0.43	FL
**61**	MOL002891	magnoflorine	0.48	0.55	CP
**62**	MOL002901	phellodendrine	2.5	0.58	CP
**63**	MOL003005	(-)-(3R,8S,9R,9aS,10aS)-9-ethenyl-8-	3.22	0.8	FL
**64**	MOL003008	madreselvin B	3.01	0.26	FL
**65**	MOL003009	Ochnaflavone	2.54	0.57	FL
**66**	MOL003010	quercetin-3-o-beta-D-glu	1.81	0.79	FL
**67**	MOL003011	Secologanate	17.56	0.33	FL
**68**	MOL003013	secologanic dibutylacetal	20.05	0.67	FL
**69**	MOL003015	2H-Pyran-5-carboxylic acid, 4-(2,2-di	22.59	0.45	FL
**70**	MOL003018	SCG	23.59	0.36	FL
**71**	MOL003020	secologanoside 7-methylester	3.88	0.45	FL
**72**	MOL003022	Secoxyloganin	3.79	0.39	FL
**73**	MOL003025	vogeloside	5.62	0.46	FL
**74**	MOL003030	Ginnol	11.33	0.43	FL
**75**	MOL003035	stigmasterol-β-glucoside	2.4	0.63	FL
**76**	MOL003037	hydnocarpinhydnocarpinhydnocarpin	2.06	0.94	FL
**77**	MOL003048	methyl chlorogenate	9.97	0.36	FL
**78**	MOL003051	Scolymoside	3.84	0.73	FL
**79**	MOL003054	eriodictyol-7-o-glucoside	17.57	0.78	FL
**80**	MOL003061	PHYTANTRIOL	23.2	0.18	FL
**81**	MOL003063	Octadecylglycol	16.18	0.19	FL
**82**	MOL003065	4-caffeoylquinic acid	10.48	0.33	FL
**83**	MOL003066	Neochlorogenic acid	10.65	0.33	FL
**84**	MOL003067	3,4-Dicaffeoylquinic acid	1.71	0.69	FL
**85**	MOL003068	4,5-Dicaffeoylquinic acid	1.73	0.69	FL
**86**	MOL003071	secologanoside	26.92	0.37	FL
**87**	MOL003073	8-epiloganin	11.68	0.44	FL
**88**	MOL003075	3,4-di-O-caffeoylquinic acid methyl e	1.71	0.69	FL
**89**	MOL003076	3,5-di-O-caffeoylquinic acid methyl e	1.73	0.68	FL
**90**	MOL003077	4,5-di-O-caffeoylquinic acid methyl e	1.73	0.69	FL
**91**	MOL003079	(1alpha,3R,4alpha,5R)-3,4,5-Tris[[(2I	3.01	0.45	FL
**92**	MOL003092	4-stearylmorpholine	14.8	0.27	FL
**93**	MOL003093	5-O-Caffeoyl quinic acid butyl ester	8.77	0.41	FL
**94**	MOL003096	2-(3,4-dimethoxyphenyl)-5-hydroxy-7	29.24	0.34	FL
**95**	MOL003097	Flavone der.	27.12	0.27	FL
**96**	MOL003098	2-(2,4-dimethoxyphenyl)-3-hydroxy-7	12.94	0.33	FL
**97**	MOL003099	7-epi-Loganin	4.78	0.44	FL
**98**	MOL003107	Akebiasaponin D_qt	16.44	0.74	FL
**99**	MOL003109	Caeruloside C_qt	5.4	0.37	FL
**100**	MOL003110	Centauroside	4.37	0.43	FL
**101**	MOL003113	Dehydroxymorroniside	20.69	0.46	FL
**102**	MOL003116	Ioniceracetalides B	5.48	0.63	FL
**103**	MOL003118	Isochlorogenic acid C	1.78	0.69	FL
**104**	MOL003119	Loniceracetalide A	28.29	0.58	FL

**105**	MOL003121	Loniceracetalide B	10.77	0.58	FL
**106**	MOL003130	madreselvin A	6.03	0.59	FL
**107**	MOL003304	Hentriacontan	8.07	0.51	HM
**108**	MOL003867	Paeonolide	6.3	0.64	CM
**109**	MOL003959	limonin	21.3	0.57	CP
**110**	MOL004368	Hyperin	6.94	0.77	CP、FL
**111**	MOL005090	oxypaeoniflorin_qt	19.4	0.44	CM
**112**	MOL005093	Diosmin	12.7	0.66	HM
**113**	MOL006276	SMR000232320	28.45	0.81	CP
**114**	MOL006314	Canthin-6-one	28.1	0.22	CP
**115**	MOL006384	4-[(1R,3aS,4R,6aS)-4-(4-hydroxy-3,5	3.29	0.72	CP
**116**	MOL006423	vanilloloside	18.66	0.21	CP
**117**	MOL007002	paeonioflorin	10.22	0.79	CM
**118**	MOL007006	oxypaeoniflorin	12.98	0.78	CM
**119**	MOL007364	apiopaeonoside	16.73	0.64	CM
**120**	MOL007365	galloyl-oxypaeoniflorin	3.2	0.39	CM
**121**	MOL007366	galloyl-oxypaeoniflorin_qt 2	26.21	0.44	CM
**122**	MOL007367	paeonoside	18.52	0.24	CM
**123**	MOL007368	4-O-methylpaeoniflorin	25.71	0.78	CM
**124**	MOL007370	6-o-vanillyoxypaeoniflorin	10.91	0.54	CM
**125**	MOL007371	6-o-vanillyoxypaeoniflorin_qt 2	17.71	0.37	CM
**126**	MOL007375	beta-sitosterol-beta-d-glucoside	19.58	0.62	CM
**127**	MOL007376	beta-sitosterol-beta-d-glucoside_qt	25.32	0.75	CM
**128**	MOL007377	mudanoside A	13.39	0.29	CM
**129**	MOL007379	3-[(2S,3R,4S,5S,6R)-6-[[(2R,3R,4R)-	5.6	0.63	CM
**130**	MOL007380	mudanpinoic,acid,a	13.86	0.65	CM
**131**	MOL007381	mudanpioside-h	6.77	0.61	CM
**132**	MOL007383	paeonidanin	24.64	0.78	CM
**133**	MOL007385	octadecyl (E)-3-(3,4-dihydroxyphenyl	3.18	0.55	CM
**134**	MOL009072	Prunin	9.33	0.74	HM
**135**	MOL009092	Pentagalloylglucose	3.01	0.21	CM
**136**	MOL011608	1,3-bis-[2-(3,4-dihydroxyphenyl)-1-ca	3.01	0.44	HM
**137**	MOL011609	1,3-bis[2-(3,4-dihydroxyphenyl)-1-me	3.01	0.41	HM
**138**	MOL011610	3-[2-(3,4-dihydroxyphenyl)-1-carbox	2.97	0.77	HM
**139**	MOL011612	7,8-dihydroxy-2-(3,4-dihydroxypheny	23.81	0.4	HM
**140**	MOL011614	1-(3,4-dihydroxyphenyl)-3-[2-(3,4-dil	2.97	0.76	HM
**141**	MOL011617	(1S,2R)-1-(3,4-dihydroxyphenyl)-6,7-	17.53	0.4	HM
**142**	MOL011619	1-[2-(3,4- dihydroxyphenyl)-1-metho	3.01	0.44	HM
**143**	MOL011620	Eriocitrin	4.52	0.7	HM
**144**	MOL011621	5-hydroxy-2-(4-hydroxyphenyl)-7-[(2	7.86	0.75	HM
**145**	MOL011623	1-[2-(3,4-dihydroxyphenyl)-1-carbox	3.01	0.42	HM
**146**	MOL011624	1-[2-(3,4-dihydroxyphenyl)-1-carbox	2.97	0.77	HM
**147**	MOL011865	rosmarinic acid	1.38	0.35	HM
**148**	MOL013068	Oroxindin	7.07	0.77	RA
**149**	MOL013434	Auraptene	25.62	0.24	CP
**150**	MOL001551	Trochol	15.48	0.78	CM

PHF: Pentaherbs Formula; AD: atopic dermatitis. OB: oral bioavailability; DL: drug-likeness; CM:

Table S3. Detailed information of human disease targets of atopic dermatitis

	Target	Symbol
1	15-Hydroxyprostaglandin Dehydrogenase	HPGD
2	2-Aminoethanethiol Dioxygenase	ADO
3	3-Hydroxy-3-Methylglutaryl-CoA Reductase	HMGCR
4	5-Hydroxytryptamine Receptor 1A	HTR1A
5	5-Hydroxytryptamine Receptor 2A	HTR2A
6	5-Hydroxytryptamine Receptor 3A	HTR3A
7	Abhydrolase Domain Containing 5	ABHD5
8	ABL Proto-Oncogene 2, Non-Receptor Tyrosine Kinase	ABL2
9	Absent In Melanoma 2	AIM2
10	Acetylcholinesterase (Cartwright Blood Group)	ACHE
11	Acid Phosphatase 1	ACP1
12	Actin Like 9	ACTL9
13	Actin Related Protein T1	ACTRT1
14	Activating Transcription Factor 6 Beta	ATF6B
15	ADAM Metallopeptidase Domain 10	ADAM10
16	ADAM Metallopeptidase Domain 33	ADAM33
17	ADAM Metallopeptidase Domain 8	ADAM8
18	ADAM Metallopeptidase With Thrombospondin Type 1 Motif 10	ADAMTS10
19	Adenosine A2b Receptor	ADORA2B
20	Adenosine Deaminase	ADA
21	Adenylate Cyclase 10	ADCY10
22	Adhesion G Protein-Coupled Receptor E1	ADGRE1
23	Adhesion G Protein-Coupled Receptor E3	ADGRE3
24	Adiponectin, C1Q And Collagen Domain Containing	ADIPOQ
25	ADP-Ribosylarginine Hydrolase	ADPRH
26	Adrenoceptor Alpha 1B	ADRA1B
27	Adrenoceptor Beta 1	ADRB1
28	Adrenoceptor Beta 2	ADRB2
29	Adrenoceptor Beta 3	ADRB3
30	Adrenomedullin	ADM
31	Adrenomedullin 2	ADM2
32	Advanced Glycosylation End-Product Specific Receptor	AGER
33	AKT Serine/Threonine Kinase 1	AKT1
34	AKT Serine/Threonine Kinase 3	AKT3
35	Alanyl Aminopeptidase, Membrane	ANPEP
36	Albumin	ALB
37	Alcohol Dehydrogenase 1B (Class I), Beta Polypeptide	ADH1B
38	Alcohol Dehydrogenase 1C (Class I), Gamma Polypeptide	ADH1C
39	Aldehyde Dehydrogenase 1 Family Member A1	ALDH1A1
40	Aldehyde Dehydrogenase 2 Family Member	ALDH2
41	Aldo-Keto Reductase Family 1 Member C3	AKR1C3
42	ALK Receptor Tyrosine Kinase	ALK
43	Alkaline Ceramidase 1	ACER1
44	Alkaline Phosphatase, Biomineralization Associated	ALPL
45	Alkylglycerone Phosphate Synthase	AGPS
46	Allergic Rhinitis	ALRH
47	Allograft Inflammatory Factor 1	AIF1
48	Alpha-1-Microglobulin/Bikunin Precursor	AMBP
49	Amine Oxidase Copper Containing 1	AOC1
50	Amine Oxidase Copper Containing 3	AOC3
51	Aminoacylase 1	ACY1
52	Amphiregulin	AREG
53	Androgen Receptor	AR
54	Angiotensin I Converting Enzyme	ACE

**55**	Angiotensinogen	AGT
**56**	ANKH Inorganic Pyrophosphate Transport Regulator	ANKH
**57**	Ankyrin Repeat Domain 1	ANKRD1
**58**	Annexin A1	ANXA1
**59**	Annexin A2	ANXA2
**60**	Annexin A5	ANXA5
**61**	Anosmin 1	ANOS1
**62**	Apelin	APLN
**63**	Apolipoprotein A1	APOA1
**64**	Apolipoprotein C1	APOC1
**65**	Apolipoprotein E	APOE
**66**	Aquaporin 3 (Gill Blood Group)	AQP3
**67**	Arachidonate 15-Lipoxygenase	ALOX15
**68**	Arachidonate 5-Lipoxygenase	ALOX5
**69**	Arachidonate 5-Lipoxygenase Activating Protein	ALOX5AP
**70**	Aralkylamine N-Acetyltransferase	AANAT
**71**	Arginase 1	ARG1
**72**	Arginine Vasopressin	AVP
**73**	Artemin	ARTN
**74**	Aryl Hydrocarbon Receptor	AHR
**75**	Aryl Hydrocarbon Receptor Interacting Protein	AIP
**76**	Aryl Hydrocarbon Receptor Nuclear Translocator	ARNT
**77**	Aspartic Peptidase Retroviral Like 1	ASPRV1
**78**	Asthma-Related Traits, Susceptibility To, 3	ASRT3
**79**	Asthma-Related Traits, Susceptibility To, 4	ASRT4
**80**	Asthma-Related Traits, Susceptibility To, 6	ASRT6
**81**	Asthma-Related Traits, Susceptibility To, 8	ASRT8
**82**	ATP Binding Cassette Subfamily C Member 6	ABCC6
**83**	ATP6V1G2-DDX39B Readthrough (NMD Candidate)	ATP6V1G2-DDX39B
**84**	ATPase H+/K+ Transporting Non-Gastric Alpha2 Subunit	ATP12A
**85**	ATPase H+/K+ Transporting Subunit Alpha	ATP4A
**86**	AT-Rich Interaction Domain 1B	ARID1B
**87**	Atypical Chemokine Receptor 1 (Duffy Blood Group)	ACKR1
**88**	Atypical Chemokine Receptor 2	ACKR2
**89**	Atypical Chemokine Receptor 4	ACKR4
**90**	Azurocidin 1	AZU1
**91**	Baculoviral IAP Repeat Containing 2	BIRC2
**92**	Baculoviral IAP Repeat Containing 3	BIRC3
**93**	BAF Chromatin Remodeling Complex Subunit BCL11B	BCL11B
**94**	BAF Chromatin Remodeling Complex Subunit BCL7B	BCL7B
**95**	Basic Helix-Loop-Helix Family Member E40	BHLHE40
**96**	Basigin (Ok Blood Group)	BSG
**97**	Bazex Syndrome	BZX
**98**	BCL10 Immune Signaling Adaptor	BCL10
**99**	BCL2 Apoptosis Regulator	BCL2
**100**	BCL2 Associated X, Apoptosis Regulator	BAX
**101**	BCL2 Like 1	BCL2L1
**102**	BCL2 Related Protein A1	BCL2A1
**103**	BCL3 Transcription Coactivator	BCL3
**104**	BCL6 Transcription Repressor	BCL6
**105**	BCR Activator Of RhoGEF And GTPase	BCR
**106**	Beta-2-Microglobulin	B2M
**107**	Beta-Secretase 1	BACE1
**108**	BH3 Interacting Domain Death Agonist	BID
**109**	Biotinidase	BTD
**110**	Bleomycin Hydrolase	BLMH
**111**	Bone Gamma-Carboxyglutamate Protein	BGLAP
**112**	Bone Morphogenetic Protein 6	BMP6

**113**	Bradykinin Receptor B1	BDKRB1
**114**	B-Raf Proto-Oncogene, Serine/Threonine Kinase	BRAF
**115**	Brain Derived Neurotrophic Factor	BDNF
**116**	BRCA2 DNA Repair Associated	BRCA2
**117**	Butyrylcholinesterase	BCHE
**118**	Cadherin 1	CDH1
**119**	Cadherin 3	CDH3
**120**	Cadherin 5	CDH5
**121**	Cadherin Related 23	CDH23
**122**	Cadherin Related Family Member 3	CDHR3
**123**	Calbindin 2	CALB2
**124**	Calcineurin Binding Protein 1	CABIN1
**125**	Calcitonin Related Polypeptide Alpha	CALCA
**126**	Calcium Sensing Receptor	CASR
**127**	Calcium Voltage-Gated Channel Subunit Alpha1 B	CACNA1B
**128**	Calmodulin Like 5	CALML5
**129**	Calnexin	CANX
**130**	CAMP Responsive Element Binding Protein 1	CREB1
**131**	Capping Protein Regulator And Myosin 1 Linker 2	CARMIL2
**132**	Carbohydrate Sulfotransferase 8	CHST8
**133**	Carbonic Anhydrase 2	CA2
**134**	Carbonic Anhydrase 3	CA3
**135**	Carboxypeptidase A3	CPA3
**136**	Carboxypeptidase Q	CPQ
**137**	Casein Alpha S1	CSN1S1
**138**	Casein Beta	CSN2
**139**	Casein Kappa	CSN3
**140**	Caspase 1	CASP1
**141**	Caspase 14	CASP14
**142**	Caspase 3	CASP3
**143**	Caspase 7	CASP7
**144**	Caspase 8	CASP8
**145**	Caspase Recruitment Domain Family Member 11	CARD11
**146**	Caspase Recruitment Domain Family Member 14	CARD14
**147**	Caspase Recruitment Domain Family Member 8	CARD8
**148**	Catalase	CAT
**149**	Catenin Alpha 2	CTNNA2
**150**	Catenin Alpha 3	CTNNA3
**151**	Cathelicidin Antimicrobial Peptide	CAMP
**152**	Cathepsin D	CTSD
**153**	Cathepsin E	CTSE
**154**	Cathepsin G	CTSG
**155**	Cathepsin L	CTSL
**156**	Cathepsin S	CTSS
**157**	Cathepsin V	CTSV
**158**	Caudal Type Homeobox 1	CDX1
**159**	Cbl Proto-Oncogene	CBL
**160**	C-C Motif Chemokine Ligand 1	CCL1
**161**	C-C Motif Chemokine Ligand 11	CCL11
**162**	C-C Motif Chemokine Ligand 13	CCL13
**163**	C-C Motif Chemokine Ligand 17	CCL17
**164**	C-C Motif Chemokine Ligand 18	CCL18
**165**	C-C Motif Chemokine Ligand 19	CCL19
**166**	C-C Motif Chemokine Ligand 2	CCL2
**167**	C-C Motif Chemokine Ligand 20	CCL20
**168**	C-C Motif Chemokine Ligand 21	CCL21
**169**	C-C Motif Chemokine Ligand 22	CCL22
**170**	C-C Motif Chemokine Ligand 23	CCL23

**171**	C-C Motif Chemokine Ligand 24	CCL24
**172**	C-C Motif Chemokine Ligand 26	CCL26
**173**	C-C Motif Chemokine Ligand 27	CCL27
**174**	C-C Motif Chemokine Ligand 28	CCL28
**175**	C-C Motif Chemokine Ligand 3	CCL3
**176**	C-C Motif Chemokine Ligand 4	CCL4
**177**	C-C Motif Chemokine Ligand 5	CCL5
**178**	C-C Motif Chemokine Ligand 7	CCL7
**179**	C-C Motif Chemokine Receptor 1	CCR1
**180**	C-C Motif Chemokine Receptor 10	CCR10
**181**	C-C Motif Chemokine Receptor 2	CCR2
**182**	C-C Motif Chemokine Receptor 3	CCR3
**183**	C-C Motif Chemokine Receptor 4	CCR4
**184**	C-C Motif Chemokine Receptor 5 (Gene/Pseudogene)	CCR5
**185**	C-C Motif Chemokine Receptor 6	CCR6
**186**	C-C Motif Chemokine Receptor 7	CCR7
**187**	C-C Motif Chemokine Receptor 8	CCR8
**188**	CCAAT Enhancer Binding Protein Alpha	CEBPA
**189**	CCCTC-Binding Factor	CTCF
**190**	CD14 Molecule	CD14
**191**	CD151 Molecule (Raph Blood Group)	CD151
**192**	CD160 Molecule	CD160
**193**	CD163 Molecule	CD163
**194**	CD164 Molecule	CD164
**195**	CD180 Molecule	CD180
**196**	CD19 Molecule	CD19
**197**	CD1a Molecule	CD1A
**198**	CD1c Molecule	CD1C
**199**	CD1d Molecule	CD1D
**200**	CD2 Molecule	CD2
**201**	CD200 Receptor 1	CD200R1
**202**	CD207 Molecule	CD207
**203**	CD209 Molecule	CD209
**204**	CD247 Molecule	CD247
**205**	CD27 Molecule	CD27
**206**	CD28 Molecule	CD28
**207**	CD300 Molecule Like Family Member B	CD300LB
**208**	CD300 Molecule Like Family Member D	CD300LD
**209**	CD300a Molecule	CD300A
**210**	CD300c Molecule	CD300C
**211**	CD300e Molecule	CD300E
**212**	CD34 Molecule	CD34
**213**	CD36 Molecule	CD36
**214**	CD3g Molecule	CD3G
**215**	CD4 Molecule	CD4
**216**	CD40 Ligand	CD40LG
**217**	CD40 Molecule	CD40
**218**	CD44 Molecule (Indian Blood Group)	CD44
**219**	CD48 Molecule	CD48
**220**	CD5 Molecule Like	CD5L
**221**	CD53 Molecule	CD53
**222**	CD63 Molecule	CD63
**223**	CD69 Molecule	CD69
**224**	CD7 Molecule	CD7
**225**	CD79a Molecule	CD79A
**226**	CD80 Molecule	CD80
**227**	CD81 Molecule	CD81
**228**	CD83 Molecule	CD83

**229**	CD86 Molecule	CD86
**230**	CD8a Molecule	CD8A
**231**	CD9 Molecule	CD9
**232**	CEA Cell Adhesion Molecule 1	CEACAM1
**233**	Cell Adhesion Molecule 1	CADM1
**234**	Cellular Retinoic Acid Binding Protein 2	CRABP2
**235**	Ceruloplasmin	CP
**236**	CF Transmembrane Conductance Regulator	CFTR
**237**	Chemerin Chemokine-Like Receptor 1	CMKLR1
**238**	Chemokine Like Factor	CKLF
**239**	Chitinase 1	CHIT1
**240**	Chitinase 3 Like 1	CHI3L1
**241**	Chitinase Acidic	CHIA
**242**	Chloride Channel Accessory 1	CLCA1
**243**	Chloride Intracellular Channel 4	CLIC4
**244**	Cholesteryl Ester Transfer Protein	CETP
**245**	Chromodomain Helicase DNA Binding Protein 7	CHD7
**246**	Chromogranin A	CHGA
**247**	Chromosome 18q Deletion Syndrome	DEL18Q
**248**	Chymase 1	CMA1
**249**	Chymotrypsin Like	CTRL
**250**	Ciliary Neurotrophic Factor	CNTF
**251**	Ciliary Rootlet Coiled-Coil, Rootletin	CROCC
**252**	Class II Major Histocompatibility Complex Transactivator	CIITA
**253**	Claudin 1	CLDN1
**254**	Claudin 3	CLDN3
**255**	Claudin 4	CLDN4
**256**	Claudin 7	CLDN7
**257**	Coagulation Factor II Thrombin Receptor	F2R
**258**	Coagulation Factor II Thrombin Receptor Like 2	F2RL2
**259**	Coagulation Factor II, Thrombin	F2
**260**	Coiled-Coil Alpha-Helical Rod Protein 1	CCHCR1
**261**	Coiled-Coil Domain Containing 80	CCDC80
**262**	Coilin	COIL
**263**	Collagen Type I Alpha 1 Chain	COL1A1
**264**	Collagen Type VI Alpha 3 Chain	COL6A3
**265**	Collagen Type VI Alpha 4 Pseudogene 1	COL6A4P1
**266**	Collagen Type VI Alpha 5 Chain	COL6A5
**267**	Collagen Type VI Alpha 6 Chain	COL6A6
**268**	Collagen Type VII Alpha 1 Chain	COL7A1
**269**	Collagen Type XXVI Alpha 1 Chain	COL26A1
**270**	Colony Stimulating Factor 1	CSF1
**271**	Colony Stimulating Factor 2	CSF2
**272**	Colony Stimulating Factor 2 Receptor Alpha Subunit	CSF2RA
**273**	Colony Stimulating Factor 3	CSF3
**274**	Complement C3	C3
**275**	Complement C3a Receptor 1	C3AR1
**276**	Complement C4A (Rodgers Blood Group)	C4A
**277**	Complement C4B (Chido Blood Group)	C4B
**278**	Complement C5	C5
**279**	Complement C5a Receptor 1	C5AR1
**280**	Coproporphyrinogen Oxidase	CPOX
**281**	Corneodesmosin	CDSN
**282**	Cornifelin	CNFN
**283**	Cornulin	CRNN
**284**	Corticotropin Releasing Hormone	CRH
**285**	Corticotropin Releasing Hormone Receptor 1	CRHR1
**286**	C-Reactive Protein	CRP

**287**	C-Type Lectin Domain Containing 16A	CLEC16A
**288**	C-Type Lectin Domain Containing 7A	CLEC7A
**289**	C-Type Lectin Domain Family 4 Member D	CLEC4D
**290**	C-Type Lectin Domain Family 4 Member E	CLEC4E
**291**	C-X3-C Motif Chemokine Ligand 1	CX3CL1
**292**	C-X3-C Motif Chemokine Receptor 1	CX3CR1
**293**	C-X-C Motif Chemokine Ligand 1	CXCL1
**294**	C-X-C Motif Chemokine Ligand 10	CXCL10
**295**	C-X-C Motif Chemokine Ligand 11	CXCL11
**296**	C-X-C Motif Chemokine Ligand 12	CXCL12
**297**	C-X-C Motif Chemokine Ligand 16	CXCL16
**298**	C-X-C Motif Chemokine Ligand 17	CXCL17
**299**	C-X-C Motif Chemokine Ligand 2	CXCL2
**300**	C-X-C Motif Chemokine Ligand 5	CXCL5
**301**	C-X-C Motif Chemokine Ligand 8	CXCL8
**302**	C-X-C Motif Chemokine Ligand 9	CXCL9
**303**	C-X-C Motif Chemokine Receptor 1	CXCR1
**304**	C-X-C Motif Chemokine Receptor 2	CXCR2
**305**	C-X-C Motif Chemokine Receptor 3	CXCR3
**306**	C-X-C Motif Chemokine Receptor 4	CXCR4
**307**	C-X-C Motif Chemokine Receptor 6	CXCR6
**308**	Cyclin D1	CCND1
**309**	Cyclin Dependent Kinase Inhibitor 1A	CDKN1A
**310**	Cystatin A	CSTA
**311**	Cystatin C	CST3
**312**	Cystatin E/M	CST6
**313**	Cysteine Rich Secretory Protein 2	CRISP2
**314**	Cysteine Rich Secretory Protein 3	CRISP3
**315**	Cysteinyl Leukotriene Receptor 1	CYSLTR1
**316**	Cysteinyl Leukotriene Receptor 2	CYSLTR2
**317**	Cytochrome C Oxidase Subunit 4I2	COX4I2
**318**	Cytochrome C Oxidase Subunit 5A	COX5A
**319**	Cytochrome P450 Family 1 Subfamily A Member 1	CYP1A1
**320**	Cytochrome P450 Family 1 Subfamily A Member 2	CYP1A2
**321**	Cytochrome P450 Family 19 Subfamily A Member 1	CYP19A1
**322**	Cytochrome P450 Family 2 Subfamily C Member 19	CYP2C19
**323**	Cytochrome P450 Family 24 Subfamily A Member 1	CYP24A1
**324**	Cytochrome P450 Family 27 Subfamily A Member 1	CYP27A1
**325**	Cytochrome P450 Family 27 Subfamily B Member 1	CYP27B1
**326**	Cytochrome P450 Family 3 Subfamily A Member 4	CYP3A4
**327**	Cytochrome P450 Family 4 Subfamily F Member 22	CYP4F22
**328**	Cytochrome P450 Oxidoreductase	POR
**329**	Cytokine Inducible SH2 Containing Protein	CISH
**330**	Cytokine Receptor Like Factor 2	CRLF2
**331**	Cytotoxic T-Lymphocyte Associated Protein 4	CTLA4
**332**	D-Dopachrome Tautomerase	DDT
**333**	Decorin	DCN
**334**	Dedicator Of Cytokinesis 2	DOCK2
**335**	Dedicator Of Cytokinesis 8	DOCK8
**336**	Defensin Alpha 4	DEFA4
**337**	Defensin Alpha 5	DEFA5
**338**	Defensin Alpha 6	DEFA6
**339**	Defensin Beta 1	DEFB1
**340**	Defensin Beta 103B	DEFB103B
**341**	Defensin Beta 4A	DEFB4A
**342**	Dehydrogenase/Reductase 7	DHRS7
**343**	DENN Domain Containing 1B	DENND1B
**344**	Dermatitis, Atopic	ATOD1

**345**	Dermatitis, Atopic, 3	ATOD3
**346**	Dermatitis, atopic, 4	ATOD4
**347**	Dermatitis, Atopic, 5	ATOD5
**348**	Dermatitis, Atopic, 6	ATOD6
**349**	Dermatitis, Atopic, 8	ATOD8
**350**	Dermatitis, Atopic, 9	ATOD9
**351**	Dermatitis, Atopic, Susceptibility To, 7	ATOD7
**352**	Dermcidin	DCD
**353**	Desmin	DES
**354**	Desmocollin 1	DSC1
**355**	Desmoglein 1	DSG1
**356**	Desmoglein 4	DSG4
**357**	Desmoplakin	DSP
**358**	DExD-Box Helicase 39A	DDX39A
**359**	DExD-Box Helicase 39B	DDX39B
**360**	Dipeptidase 1	DPEP1
**361**	Dipeptidyl Peptidase 4	DPP4
**362**	Dipeptidyl Peptidase 9	DPP9
**363**	Dipeptidyl Peptidase Like 10	DPP10
**364**	Dishevelled Segment Polarity Protein 1	DVL1
**365**	Distal-Less Homeobox 3	DLX3
**366**	DMRT Like Family A1	DMRTA1
**367**	DNA Ligase 1	LIG1
**368**	DNA Methyltransferase 1	DNMT1
**369**	DNA Polymerase Epsilon, Catalytic Subunit	POLE
**370**	DNA Topoisomerase II Alpha	TOP2A
**371**	DnaJ Heat Shock Protein Family (Hsp40) Member B6	DNAJB6
**372**	DnaJ Heat Shock Protein Family (Hsp40) Member C21	DNAJC21
**373**	Dopamine Beta-Hydroxylase	DBH
**374**	Dual Specificity Phosphatase 1	DUSP1
**375**	Dynein Axonemal Heavy Chain 5	DNAH5
**376**	Dynein Axonemal Heavy Chain 8	DNAH8
**377**	Early Growth Response 1	EGR1
**378**	Early Growth Response 2	EGR2
**379**	EBP Cholestenol Delta-Isomerase	EBP
**380**	Ectodysplasin A	EDA
**381**	Ectonucleoside Triphosphate Diphosphohydrolase 6	ENTPD6
**382**	Ectonucleotide Pyrophosphatase/Phosphodiesterase 1	ENPP1
**383**	Ectonucleotide Pyrophosphatase/Phosphodiesterase 2	ENPP2
**384**	Ectonucleotide Pyrophosphatase/Phosphodiesterase 3	ENPP3
**385**	EF-Hand Domain Family Member D2	EFHD2
**386**	Elastase, Neutrophil Expressed	ELANE
**387**	Elastin	ELN
**388**	Elongation Factor 1 Homolog	ELOF1
**389**	Elongation Factor Like GTPase 1	EFL1
**390**	Elongator Complex Protein 1	ELP1
**391**	ELOVL Fatty Acid Elongase 2	ELOVL2
**392**	ELOVL Fatty Acid Elongase 5	ELOVL5
**393**	EMSY Transcriptional Repressor, BRCA2 Interacting	EMSY
**394**	Endogenous Retrovirus Group 9 Member 1	ERV9-1
**395**	Endomucin	EMCN
**396**	Endothelial Cell Specific Molecule 1	ESM1
**397**	Endothelin 1	EDN1
**398**	Endothelin Receptor Type A	EDNRA
**399**	Endothelin Receptor Type B	EDNRB
**400**	Enolase 1	ENO1
**401**	Enolase 2	ENO2
**402**	Eosinophil Peroxidase	EPX

**403**	EPH Receptor B2	EPHB2
**404**	Epidermal Growth Factor	EGF
**405**	Epidermal Growth Factor Receptor	EGFR
**406**	Epidermal Growth Factor Receptor Pathway Substrate 15	EPS15
**407**	ER Membrane Protein Complex Subunit 3	EMC3
**408**	Erb-B2 Receptor Tyrosine Kinase 2	ERBB2
**409**	Erb-B2 Receptor Tyrosine Kinase 4	ERBB4
**410**	ERCC Excision Repair 2, TFIIH Core Complex Helicase Subunit	ERCC2
**411**	ERCC Excision Repair 3, TFIIH Core Complex Helicase Subunit	ERCC3
**412**	ERCC Excision Repair 4, Endonuclease Catalytic Subunit	ERCC4
**413**	Erythropoietin	EPO
**414**	Esterase D	ESD
**415**	Estrogen Receptor 1	ESR1
**416**	ETS Proto-Oncogene 1, Transcription Factor	ETS1
**417**	ETS Proto-Oncogene 2, Transcription Factor	ETS2
**418**	Euchromatic Histone Lysine Methyltransferase 2	EHMT2
**419**	Eukaryotic Translation Initiation Factor 2 Alpha Kinase 3	EIF2AK3
**420**	F2R Like Thrombin Or Trypsin Receptor 3	F2RL3
**421**	F2R Like Trypsin Receptor 1	F2RL1
**422**	Fas Associated Factor 1	FAF1
**423**	Fas Associated Factor Family Member 2	FAF2
**424**	Fas Cell Surface Death Receptor	FAS
**425**	Fas Ligand	FASLG
**426**	FAT Atypical Cadherin 4	FAT4
**427**	Fatty Acid Binding Protein 5	FABP5
**428**	Fatty Acid Desaturase 1	FADS1
**429**	Fatty Acid Desaturase 2	FADS2
**430**	Fatty Acid Synthase	FASN
**431**	Fc Fragment Of IgE Receptor Ia	FCER1A
**432**	Fc Fragment Of IgE Receptor Ig	FCER1G
**433**	Fc Fragment Of IgE Receptor II	FCER2
**434**	Fc Fragment Of IgG Receptor And Transporter	FCGRT
**435**	Fc Fragment Of IgG Receptor Ia	FCGR1A
**436**	Fc Fragment Of IgG Receptor IIa	FCGR2A
**437**	Fc Fragment Of IgG Receptor IIb	FCGR2B
**438**	Fc Fragment Of IgG Receptor IIc (Gene/Pseudogene)	FCGR2C
**439**	Fc Fragment Of IgG Receptor IIIa	FCGR3A
**440**	Fc Fragment Of IgG Receptor IIIb	FCGR3B
**441**	Fermitin Family Member 3	FERMT3
**442**	Ferrochelatase	FECH
**443**	Fibrillin 1	FBN1
**444**	Fibrinogen Alpha Chain	FGA
**445**	Fibroblast Activation Protein Alpha	FAP
**446**	Fibroblast Growth Factor 5	FGF5
**447**	Fibroblast Growth Factor 7	FGF7
**448**	Fibroblast Growth Factor Binding Protein 2	FGFBP2
**449**	Fibroblast Growth Factor Receptor 2	FGFR2
**450**	Fibulin 5	FBLN5
**451**	Ficolin 3	FCN3
**452**	Filaggrin	FLG
**453**	Filaggrin Family Member 2	FLG2
**454**	FKBP Prolyl Isomerase 1A	FKBP1A
**455**	FKBP Prolyl Isomerase 5	FKBP5
**456**	FKBP Prolyl Isomerase 8	FKBP8
**457**	FLG Antisense RNA 1	FLG-AS1
**458**	Fli-1 Proto-Oncogene, ETS Transcription Factor	FLI1
**459**	Fms Related Tyrosine Kinase 4	FLT4
**460**	Forkhead Box J1	FOXJ1

**461**	Forkhead Box P3	FOXP3
**462**	Fos Proto-Oncogene, AP-1 Transcription Factor Subunit	FOS
**463**	Fucosyltransferase 2	FUT2
**464**	Fucosyltransferase 4	FUT4
**465**	Fucosyltransferase 7	FUT7
**466**	G Protein Signaling Modulator 3	GPSM3
**467**	G Protein Subunit Alpha 11	GNA11
**468**	G Protein Subunit Alpha I1	GNAI1
**469**	Galactosidase Beta 1	GLB1
**470**	Galanin And GMAP Prepropeptide	GAL
**471**	Galectin 1	LGALS1
**472**	Galectin 3	LGALS3
**473**	Galectin 7	LGALS7
**474**	Galectin 9	LGALS9
**475**	Gamma-Glutamyltransferase 1	GGT1
**476**	Gap Junction Protein Alpha 1	GJA1
**477**	Gap Junction Protein Beta 2	GJB2
**478**	Gastrin Releasing Peptide	GRP
**479**	GATA Binding Protein 1	GATA1
**480**	GATA Binding Protein 3	GATA3
**481**	Gelsolin	GSN
**482**	General Transcription Factor IIE Subunit 2	GTF2E2
**483**	General Transcription Factor IIH Subunit 5	GTF2H5
**484**	GLI Family Zinc Finger 3	GLI3
**485**	Glucocorticoid Induced 1	GLCCI1
**486**	Glucosylceramidase Beta	GBA
**487**	Glucuronidase Beta	GUSB
**488**	Glutathione S-Transferase Mu 1	GSTM1
**489**	Glutathione S-Transferase Pi 1	GSTP1
**490**	Glutathione S-Transferase Theta 1	GSTT1
**491**	Glutathione-Disulfide Reductase	GSR
**492**	Glyceraldehyde-3-Phosphate Dehydrogenase	GAPDH
**493**	Glycogen Synthase Kinase 3 Beta	GSK3B
**494**	Glyoxalase I	GLO1
**495**	Grainyhead Like Transcription Factor 2	GRHL2
**496**	Grancalcin	GCA
**497**	Granzyme B	GZMB
**498**	Growth Factor Independent 1 Transcriptional Repressor	GFI1
**499**	Growth Factor Receptor Bound Protein 7	GRB7
**500**	H2.0 Like Homeobox	HLX
**501**	Heat Shock Protein 90 Alpha Family Class A Member 1	HSP90AA1
**502**	Heat Shock Protein Family A (Hsp70) Member 4	HSPA4
**503**	Heat Shock Protein Family A (Hsp70) Member 9	HSPA9
**504**	Heat Shock Protein Family B (Small) Member 1	HSPB1
**505**	Heat Shock Protein Family B (Small) Member 2	HSPB2
**506**	Heat Shock Protein Family D (Hsp60) Member 1	HSPD1
**507**	Heme Oxygenase 1	HMOX1
**508**	Hemoglobin Subunit Beta	HBB
**509**	Hemojuvelin BMP Co-Receptor	HJV
**510**	Heparan Sulfate Proteoglycan 2	HSPG2
**511**	Heparin Binding EGF Like Growth Factor	HBEGF
**512**	Hepatitis A Virus Cellular Receptor 1	HAVCR1
**513**	Hepatitis A Virus Cellular Receptor 2	HAVCR2
**514**	Hepcidin Antimicrobial Peptide	HAMP
**515**	High Mobility Group Box 1	HMGB1
**516**	Histamine N-Methyltransferase	HNMT
**517**	Histamine Receptor H1	HRH1
**518**	Histamine Receptor H2	HRH2

**519**	Histamine Receptor H4	HRH4
**520**	Histidine Decarboxylase	HDC
**521**	Histone Deacetylase 2	HDAC2
**522**	HLA Complex Group 27	HCG27
**523**	Homeostatic Iron Regulator	HFE
**524**	Hornerin	HRNR
**525**	HRas Proto-Oncogene, GTPase	HRAS
**526**	Hyaluronan Synthase 1	HAS1
**527**	Hyaluronan Synthase 2	HAS2
**528**	Hyaluronan Synthase 3	HAS3
**529**	Hydroxy-Delta-5-Steroid Dehydrogenase, 3 Beta- And Steroid Delta-Isomerase 2	HSD3B2
**530**	Hydroxysteroid 11-Beta Dehydrogenase 1	HSD11B1
**531**	Hypoxia Inducible Factor 1 Subunit Alpha	HIF1A
**532**	IL2 Inducible T Cell Kinase	ITK
**533**	Immediate Early Response 3 Interacting Protein 1	IER3IP1
**534**	Immunoglobulin E Concentration, Serum	IGES
**535**	Immunoglobulin Heavy Constant Epsilon	IGHE
**536**	Immunoglobulin Heavy Variable 1/OR21-1 (Non-Functional)	IGHV1OR21-1
**537**	Indoleamine 2,3-Dioxygenase 1	IDO1
**538**	Inducible T Cell Costimulator	ICOS
**539**	Inducible T Cell Costimulator Ligand	ICOSLG
**540**	Inhibitor Of Nuclear Factor Kappa B Kinase Regulatory Subunit Gamma	IKBKG
**541**	Insulin	INS
**542**	Insulin Like Growth Factor 1	IGF1
**543**	Insulin Like Growth Factor 2	IGF2
**544**	Insulin Like Growth Factor Binding Protein 3	IGFBP3
**545**	Integrin Subunit Alpha 1	ITGA1
**546**	Integrin Subunit Alpha 2	ITGA2
**547**	Integrin Subunit Alpha 4	ITGA4
**548**	Integrin Subunit Alpha 6	ITGA6
**549**	Integrin Subunit Alpha L	ITGAL
**550**	Integrin Subunit Alpha M	ITGAM
**551**	Integrin Subunit Alpha X	ITGAX
**552**	Integrin Subunit Beta 1	ITGB1
**553**	Integrin Subunit Beta 2	ITGB2
**554**	Integrin Subunit Beta 3	ITGB3
**555**	Integrin Subunit Beta 4	ITGB4
**556**	Intelectin 1	ITLN1
**557**	Intercellular Adhesion Molecule 1	ICAM1
**558**	Intercellular Adhesion Molecule 2	ICAM2
**559**	Intercellular Adhesion Molecule 3	ICAM3
**560**	Interferon Alpha 1	IFNA1
**561**	Interferon Alpha 2	IFNA2
**562**	Interferon Beta 1	IFNB1
**563**	Interferon Gamma	IFNG
**564**	Interferon Gamma Receptor 1	IFNGR1
**565**	Interferon Induced With Helicase C Domain 1	IFIH1
**566**	Interferon Kappa	IFNK
**567**	Interferon Lambda 1	IFNL1
**568**	Interferon Regulatory Factor 1	IRF1
**569**	Interferon Regulatory Factor 2	IRF2
**570**	Interferon Regulatory Factor 3	IRF3
**571**	Interferon Regulatory Factor 5	IRF5
**572**	Interferon Regulatory Factor 7	IRF7
**573**	Interferon, Type 1, Cluster	IFN1@
**574**	Interleukin 1 Alpha	IL1A
**575**	Interleukin 1 Beta	IL1B

**576**	Interleukin 1 Family Member 10	IL1F10
**577**	Interleukin 1 Receptor Accessory Protein	IL1RAP
**578**	Interleukin 1 Receptor Accessory Protein Like 2	IL1RAPL2
**579**	Interleukin 1 Receptor Antagonist	IL1RN
**580**	Interleukin 1 Receptor Associated Kinase 3	IRAK3
**581**	Interleukin 1 Receptor Like 1	IL1RL1
**582**	Interleukin 1 Receptor Like 2	IL1RL2
**583**	Interleukin 1 Receptor Type 1	IL1R1
**584**	Interleukin 10	IL10
**585**	Interleukin 10 Receptor Subunit Alpha	IL10RA
**586**	Interleukin 10 Receptor Subunit Beta	IL10RB
**587**	Interleukin 11	IL11
**588**	Interleukin 12 Receptor Subunit Beta 1	IL12RB1
**589**	Interleukin 12 Receptor Subunit Beta 2	IL12RB2
**590**	Interleukin 12A	IL12A
**591**	Interleukin 12B	IL12B
**592**	Interleukin 13	IL13
**593**	Interleukin 13 Receptor Subunit Alpha 1	IL13RA1
**594**	Interleukin 13 Receptor Subunit Alpha 2	IL13RA2
**595**	Interleukin 15	IL15
**596**	Interleukin 16	IL16
**597**	Interleukin 17 Receptor A	IL17RA
**598**	Interleukin 17A	IL17A
**599**	Interleukin 17C	IL17C
**600**	Interleukin 17D	IL17D
**601**	Interleukin 17F	IL17F
**602**	Interleukin 18	IL18
**603**	Interleukin 18 Binding Protein	IL18BP
**604**	Interleukin 18 Receptor 1	IL18R1
**605**	Interleukin 18 Receptor Accessory Protein	IL18RAP
**606**	Interleukin 19	IL19
**607**	Interleukin 2	IL2
**608**	Interleukin 2 Receptor Subunit Alpha	IL2RA
**609**	Interleukin 2 Receptor Subunit Beta	IL2RB
**610**	Interleukin 2 Receptor Subunit Gamma	IL2RG
**611**	Interleukin 21	IL21
**612**	Interleukin 21 Receptor	IL21R
**613**	Interleukin 22	IL22
**614**	Interleukin 23 Receptor	IL23R
**615**	Interleukin 23 Subunit Alpha	IL23A
**616**	Interleukin 24	IL24
**617**	Interleukin 25	IL25
**618**	Interleukin 26	IL26
**619**	Interleukin 27	IL27
**620**	Interleukin 3	IL3
**621**	Interleukin 3 Receptor Subunit Alpha	IL3RA
**622**	Interleukin 31	IL31
**623**	Interleukin 31 Receptor A	IL31RA
**624**	Interleukin 32	IL32
**625**	Interleukin 33	IL33
**626**	Interleukin 36 Gamma	IL36G
**627**	Interleukin 37	IL37
**628**	Interleukin 4	IL4
**629**	Interleukin 4 Receptor	IL4R
**630**	Interleukin 5	IL5
**631**	Interleukin 5 Receptor Subunit Alpha	IL5RA
**632**	Interleukin 6	IL6
**633**	Interleukin 6 Receptor	IL6R

**634**	Interleukin 6 Signal Transducer	IL6ST
**635**	Interleukin 7	IL7
**636**	Interleukin 7 Receptor	IL7R
**637**	Interleukin 9	IL9
**638**	Interleukin 9 Receptor	IL9R
**639**	Intersectin 2	ITSN2
**640**	Involucrin	IVL
**641**	IQ Motif Containing GTPase Activating Protein 1	IQGAP1
**642**	Itchy E3 Ubiquitin Protein Ligase	ITCH
**643**	IVL Promoter Region	LOC107880064
**644**	Janus Kinase 1	JAK1
**645**	Janus Kinase 2	JAK2
**646**	Janus Kinase 3	JAK3
**647**	Janus Kinase And Microtubule Interacting Protein 1	JAKMIP1
**648**	Joining Chain Of Multimeric IgA And IgM	JCHAIN
**649**	Jun Proto-Oncogene, AP-1 Transcription Factor Subunit	JUN
**650**	Junction Plakoglobin	JUP
**651**	Kallikrein B1	KLKB1
**652**	Kallikrein Related Peptidase 11	KLK11
**653**	Kallikrein Related Peptidase 5	KLK5
**654**	Kallikrein Related Peptidase 6	KLK6
**655**	Kallikrein Related Peptidase 7	KLK7
**656**	Katanin Catalytic Subunit A1 Like 1	KATNAL1
**657**	Kelch Domain Containing 1	KLHDC1
**658**	Keratin 1	KRT1
**659**	Keratin 10	KRT10
**660**	Keratin 14	KRT14
**661**	Keratin 16	KRT16
**662**	Keratin 17	KRT17
**663**	Keratin 18	KRT18
**664**	Keratin 19	KRT19
**665**	Keratin 5	KRT5
**666**	Keratin 6A	KRT6A
**667**	Keratin 7	KRT7
**668**	Keratin 75	KRT75
**669**	Keratin 8	KRT8
**670**	KIAA1109	KIAA1109
**671**	Killer Cell Immunoglobulin Like Receptor, Two Ig Domains And Long Cytoplasmic Tail 2	KIR2DL2
**672**	Killer Cell Immunoglobulin Like Receptor, Two Ig Domains And Long Cytoplasmic Tail 3	KIR2DL3
**673**	Killer Cell Immunoglobulin Like Receptor, Two Ig Domains And Short Cytoplasmic Tail 1	KIR2DS1
**674**	Killer Cell Immunoglobulin Like Receptor, Two Ig Domains And Short Cytoplasmic Tail 2	KIR2DS2
**675**	Killer Cell Lectin Like Receptor K1	KLRK1
**676**	Kinesin Family Member 3A	KIF3A
**677**	Kininogen 1	KNG1
**678**	KIT Ligand	KITLG
**679**	KIT Proto-Oncogene, Receptor Tyrosine Kinase	KIT
**680**	Kondoh Syndrome	KONDS
**681**	KRAS Proto-Oncogene, GTPase	KRAS
**682**	Kynureninase	KYNU
**683**	Kynurenine 3-Monooxygenase	KMO
**684**	L1 Cell Adhesion Molecule	L1CAM
**685**	Lactalbumin Alpha	LALBA
**686**	Lactoperoxidase	LPO
**687**	Lactotransferrin	LTF

**688**	Lamin B Receptor	LBR
**689**	Laminin Subunit Alpha 3	LAMA3
**690**	Laminin Subunit Beta 3	LAMB3
**691**	Laminin Subunit Gamma 2	LAMC2
**692**	Late Cornified Envelope 3A	LCE3A
**693**	Late Cornified Envelope 3B	LCE3B
**694**	Late Cornified Envelope 3C	LCE3C
**695**	Late Cornified Envelope 5A	LCE5A
**696**	Late Cornified Envelope Like Proline Rich 1	LELP1
**697**	LDL Receptor Related Protein 1	LRP1
**698**	Legumain	LGMN
**699**	Legumain Pseudogene 1	LGMNP1
**700**	Leptin	LEP
**701**	Leucine Rich Repeat Containing 32	LRRC32
**702**	Leukocyte Immunoglobulin Like Receptor A6	LILRA6
**703**	Leukotriene A4 Hydrolase	LTA4H
**704**	Leukotriene B4 Receptor	LTB4R
**705**	Leukotriene B4 Receptor 2	LTB4R2
**706**	Leukotriene C4 Synthase	LTC4S
**707**	LIF Interleukin 6 Family Cytokine	LIF
**708**	LIF Receptor Subunit Alpha	LIFR
**709**	Lipin 2	LPIN2
**710**	Lipocalin 2	LCN2
**711**	Long Intergenic Non-Protein Coding RNA 709	LINC00709
**712**	Long Intergenic Non-Protein Coding RNA 824	LINC00824
**713**	Loricrin	LOR
**714**	Lymphocyte Activating 3	LAG3
**715**	Lymphocyte Antigen 96	LY96
**716**	Lymphocyte Cytosolic Protein 1	LCP1
**717**	Lymphotoxin Alpha	LTA
**718**	LYN Proto-Oncogene, Src Family Tyrosine Kinase	LYN
**719**	Macrophage Migration Inhibitory Factor	MIF
**720**	Macrophage Receptor With Collagenous Structure	MARCO
**721**	Major Histocompatibility Complex, Class I, A	HLA-A
**722**	Major Histocompatibility Complex, Class I, B	HLA-B
**723**	Major Histocompatibility Complex, Class I, C	HLA-C
**724**	Major Histocompatibility Complex, Class I, G	HLA-G
**725**	Major Histocompatibility Complex, Class II, DM Alpha	HLA-DMA
**726**	Major Histocompatibility Complex, Class II, DM Beta	HLA-DMB
**727**	Major Histocompatibility Complex, Class II, DO Alpha	HLA-DOA
**728**	Major Histocompatibility Complex, Class II, DP Alpha 1	HLA-DPA1
**729**	Major Histocompatibility Complex, Class II, DP Beta 1	HLA-DPB1
**730**	Major Histocompatibility Complex, Class II, DQ Alpha 1	HLA-DQA1
**731**	Major Histocompatibility Complex, Class II, DQ Alpha 2	HLA-DQA2
**732**	Major Histocompatibility Complex, Class II, DQ Beta 1	HLA-DQB1
**733**	Major Histocompatibility Complex, Class II, DR Alpha	HLA-DRA
**734**	Major Histocompatibility Complex, Class II, DR Beta 1	HLA-DRB1
**735**	Major Histocompatibility Complex, Class II, DR Beta 5	HLA-DRB5
**736**	Major Intrinsic Protein Of Lens Fiber	MIP
**737**	Malate Dehydrogenase 2	MDH2
**738**	MALT1 Paracaspase	MALT1
**739**	Mannose Binding Lectin 2	MBL2
**740**	Mannose Receptor C-Type 1	MRC1
**741**	Matrix Metallopeptidase 1	MMP1
**742**	Matrix Metallopeptidase 10	MMP10
**743**	Matrix Metallopeptidase 12	MMP12
**744**	Matrix Metallopeptidase 13	MMP13
**745**	Matrix Metallopeptidase 2	MMP2

**746**	Matrix Metallopeptidase 3	MMP3
**747**	Matrix Metallopeptidase 7	MMP7
**748**	Matrix Metallopeptidase 8	MMP8
**749**	Matrix Metallopeptidase 9	MMP9
**750**	MCC Regulator Of WNT Signaling Pathway	MCC
**751**	MCL1 Apoptosis Regulator, BCL2 Family Member	MCL1
**752**	MDM2 Proto-Oncogene	MDM2
**753**	Mechanistic Target Of Rapamycin Kinase	MTOR
**754**	MEFV Innate Immuity Regulator, Pyrin	MEFV
**755**	Melanocortin 1 Receptor	MC1R
**756**	Melanocortin 3 Receptor	MC3R
**757**	Melanocortin 5 Receptor	MC5R
**758**	Membrane Bound Transcription Factor Peptidase, Site 2	MBTPS2
**759**	Membrane Metalloendopeptidase	MME
**760**	Membrane Spanning 4-Domains A2	MS4A2
**761**	Mesencephalic Astrocyte Derived Neurotrophic Factor	MANF
**762**	Metastasis Associated Lung Adenocarcinoma Transcript 1	MALAT1
**763**	Methylenetetrahydrofolate Reductase	MTHFR
**764**	Methylsterol Monooxygenase 1	MSMO1
**765**	MHC Class I Polypeptide-Related Sequence A	MICA
**766**	MHC Class I Polypeptide-Related Sequence B	MICB
**767**	MicroRNA 1208	MIR1208
**768**	MicroRNA 124-1	MIR124-1
**769**	MicroRNA 125b-1	MIR125B1
**770**	MicroRNA 126	MIR126
**771**	MicroRNA 143	MIR143
**772**	MicroRNA 146a	MIR146A
**773**	MicroRNA 148a	MIR148A
**774**	MicroRNA 148b	MIR148B
**775**	MicroRNA 151a	MIR151A
**776**	MicroRNA 152	MIR152
**777**	MicroRNA 155	MIR155
**778**	MicroRNA 184	MIR184
**779**	MicroRNA 19a	MIR19A
**780**	MicroRNA 203a	MIR203A
**781**	MicroRNA 21	MIR21
**782**	MicroRNA 210	MIR210
**783**	MicroRNA 223	MIR223
**784**	MicroRNA 29a	MIR29A
**785**	MicroRNA 323a	MIR323A
**786**	MicroRNA 34a	MIR34A
**787**	MicroRNA 424	MIR424
**788**	MicroRNA 483	MIR483
**789**	MicroRNA 99a	MIR99A
**790**	MIR4435-2 Host Gene	MIR4435-2HG
**791**	Mitochondrial Calcium Uptake 1	MICU1
**792**	Mitogen-Activated Protein Kinase 1	MAPK1
**793**	Mitogen-Activated Protein Kinase 14	MAPK14
**794**	Mitogen-Activated Protein Kinase 3	MAPK3
**795**	Mitogen-Activated Protein Kinase 8	MAPK8
**796**	Mitogen-Activated Protein Kinase Kinase 1	MAP2K1
**797**	Mitogen-Activated Protein Kinase Kinase 2	MAP2K2
**798**	Mitogen-Activated Protein Kinase Kinase 7	MAP2K7
**799**	Monoamine Oxidase A	MAOA
**800**	M-Phase Specific PLK1 Interacting Protein	MPLKIP
**801**	MPL Proto-Oncogene, Thrombopoietin Receptor	MPL
**802**	Mucin 1, Cell Surface Associated	MUC1
**803**	Mucin 5AC, Oligomeric Mucus/Gel-Forming	MUC5AC

**804**	Mucin 7, Secreted	MUC7
**805**	MX Dynamin Like GTPase 1	MX1
**806**	MYD88 Innate Immune Signal Transduction Adaptor	MYD88
**807**	Myelin Associated Glycoprotein	MAG
**808**	Myelin Basic Protein	MBP
**809**	Myeloperoxidase	MPO
**810**	Myosin Light Chain 10	MYL10
**811**	Myosin Light Chain 12A	MYL12A
**812**	Myosin Light Chain 2	MYL2
**813**	Myosin Light Chain 5	MYL5
**814**	Myosin Light Chain 6B	MYL6B
**815**	Myosin Light Chain 9	MYL9
**816**	Myosin Light Chain Kinase	MYLK
**817**	N-Acetyltransferase 1	NAT1
**818**	N-Acetyltransferase 2	NAT2
**819**	N-Acetyltransferase 9 (Putative)	NAT9
**820**	N-Acylsphingosine Amidohydrolase 2	ASAH2
**821**	NAD(P)H Quinone Dehydrogenase 1	NQO1
**822**	NADPH Oxidase 5	NOX5
**823**	Nascent Polypeptide Associated Complex Subunit Alpha	NACA
**824**	Nascent Polypeptide Associated Complex Subunit Alpha 2	NACA2
**825**	Natriuretic Peptide A	NPPA
**826**	Natural Cytotoxicity Triggering Receptor 2	NCR2
**827**	Natural Cytotoxicity Triggering Receptor 3	NCR3
**828**	Natural Killer Cell Cytotoxicity Receptor 3 Ligand 1	NCR3LG1
**829**	NBAS Subunit Of NRZ Tethering Complex	NBAS
**830**	Negative Elongation Factor Complex Member C/D	NELFCD
**831**	Nerve Growth Factor	NGF
**832**	Nerve Growth Factor Receptor	NGFR
**833**	Neural Cell Adhesion Molecule 1	NCAM1
**834**	Neural EGFL Like 2	NELL2
**835**	Neurofibromin 1	NF1
**836**	Neuropeptide S Receptor 1	NPSR1
**837**	Neuropeptide Y	NPY
**838**	Neurotensin	NTS
**839**	Neurotrophic Receptor Tyrosine Kinase 1	NTRK1
**840**	Neurotrophin 3	NTF3
**841**	Neurotrophin 4	NTF4
**842**	Neutrophil Cytosolic Factor 4	NCF4
**843**	NFKB Inhibitor Alpha	NFKBIA
**844**	N-Glycanase 1	NGLY1
**845**	Nicotinamide Phosphoribosyltransferase	NAMPT
**846**	NIMA Related Kinase 9	NEK9
**847**	Nitric Oxide Synthase 1	NOS1
**848**	Nitric Oxide Synthase 2	NOS2
**849**	Nitric Oxide Synthase 3	NOS3
**850**	NLR Family CARD Domain Containing 4	NLRC4
**851**	NLR Family Pyrin Domain Containing 1	NLRP1
**852**	NLR Family Pyrin Domain Containing 10	NLRP10
**853**	NLR Family Pyrin Domain Containing 12	NLRP12
**854**	NLR Family Pyrin Domain Containing 2	NLRP2
**855**	NLR Family Pyrin Domain Containing 3	NLRP3
**856**	Notch Receptor 1	NOTCH1
**857**	NPSR1 Antisense RNA 1	NPSR1-AS1
**858**	NRAS Proto-Oncogene, GTPase	NRAS
**859**	Nuclear Factor Kappa B Subunit 1	NFKB1
**860**	Nuclear Factor Of Activated T Cells 1	NFATC1
**861**	Nuclear Factor Of Activated T Cells 2	NFATC2

**862**	Nuclear Receptor Subfamily 1 Group H Member 2	NR1H2
**863**	Nuclear Receptor Subfamily 1 Group H Member 3	NR1H3
**864**	Nuclear Receptor Subfamily 1 Group I Member 2	NR1I2
**865**	Nuclear Receptor Subfamily 3 Group C Member 1	NR3C1
**866**	Nuclear Receptor Subfamily 4 Group A Member 1	NR4A1
**867**	Nuclear Receptor Subfamily 4 Group A Member 2	NR4A2
**868**	Nucleophosmin 1	NPM1
**869**	Nucleosome Assembly Protein 1 Like 2	NAP1L2
**870**	Nucleotide Binding Oligomerization Domain Containing 1	NOD1
**871**	Nucleotide Binding Oligomerization Domain Containing 2	NOD2
**872**	Occludin	OCLN
**873**	Olfactory Receptor Family 10 Subfamily A Member 3	OR10A3
**874**	Oncostatin M	OSM
**875**	Oncostatin M Receptor	OSMR
**876**	Opioid Receptor Kappa 1	OPRK1
**877**	Opioid Receptor Mu 1	OPRM1
**878**	ORAI Calcium Release-Activated Calcium Modulator 1	ORAI1
**879**	ORMDL Sphingolipid Biosynthesis Regulator 3	ORMDL3
**880**	Ornithine Decarboxylase 1	ODC1
**881**	Orosomucoid 1	ORM1
**882**	Ovo Like Transcriptional Repressor 1	OVOL1
**883**	OXA1L Mitochondrial Inner Membrane Protein	OXA1L
**884**	Paraoxonase 1	PON1
**885**	Parkinsonism Associated Deglycase	PARK7
**886**	Parvalbumin	PVALB
**887**	Patatin Like Phospholipase Domain Containing 6	PNPLA6
**888**	PBX Homeobox 2	PBX2
**889**	PC4 And SFRS1 Interacting Protein 1	PSIP1
**890**	PDZK1 Interacting Protein 1	PDZK1IP1
**891**	Peptidase D	PEPD
**892**	Peptidase Inhibitor 3	PI3
**893**	Peptidoglycan Recognition Protein 3	PGLYRP3
**894**	Peptidyl Arginine Deiminase 3	PADI3
**895**	Peptidylprolyl Isomerase A	PPIA
**896**	Perforin 1	PRF1
**897**	Periostin	POSTN
**898**	Periplakin	PPL
**899**	Peroxisome Proliferator Activated Receptor Alpha	PPARA
**900**	Peroxisome Proliferator Activated Receptor Delta	PPARD
**901**	Peroxisome Proliferator Activated Receptor Gamma	PPARG
**902**	PHD Finger Protein 11	PHF11
**903**	Phenylethanolamine N-Methyltransferase	PNMT
**904**	Phosphatidylethanolamine Binding Protein 1	PEBP1
**905**	Phosphatidylinositol Transfer Protein Beta	PITPNB
**906**	Phosphatidylinositol-4,5-Bisphosphate 3-Kinase Catalytic Subunit Alpha	PIK3CA
**907**	Phosphatidylinositol-4,5-Bisphosphate 3-Kinase Catalytic Subunit Gamma	PIK3CG
**908**	Phosphodiesterase 3B	PDE3B
**909**	Phosphodiesterase 4A	PDE4A
**910**	Phosphodiesterase 7A	PDE7A
**911**	Phosphoglucomutase 3	PGM3
**912**	Phospholipase A2 Group IIA	PLA2G2A
**913**	Phospholipase A2 Group IIE	PLA2G2E
**914**	Phospholipase A2 Group IIF	PLA2G2F
**915**	Phospholipase A2 Group IVD	PLA2G4D
**916**	Phospholipase A2 Group VI	PLA2G6
**917**	Phospholipase A2 Group VII	PLA2G7
**918**	Phospholipase C Delta 1	PLCD1
**919**	Phospholipase D1	PLD1

**920**	Phospholipid Scramblase 1	PLSCR1
**921**	Phosphomannomutase 2	PMM2
**922**	Plakophilin 2	PKP2
**923**	Plasminogen Activator, Urokinase	PLAU
**924**	Plasminogen Activator, Urokinase Receptor	PLAUR
**925**	Plastin 1	PLS1
**926**	Plastin 3	PLS3
**927**	Platelet Activating Factor Receptor	PTAFR
**928**	Platelet And Endothelial Cell Adhesion Molecule 1	PECAM1
**929**	Platelet Factor 4	PF4
**930**	Plexin B2	PLXNB2
**931**	Pogo Transposable Element Derived With ZNF Domain	POGZ
**932**	Potassium Inwardly Rectifying Channel Subfamily J Member 11	KCNJ11
**933**	POU Class 2 Homeobox 1	POU2F1
**934**	Prefoldin Subunit 4	PFDN4
**935**	Presenilin 1	PSEN1
**936**	Presenilin Enhancer, Gamma-Secretase Subunit	PSENEN
**937**	Prion Protein	PRNP
**938**	Prodynorphin	PDYN
**939**	Programmed Cell Death 1	PDCD1
**940**	Prohibitin	PHB
**941**	Prolactin	PRL
**942**	Prolactin Induced Protein	PIP
**943**	Proopiomelanocortin	POMC
**944**	Propionyl-CoA Carboxylase Subunit Alpha	PCCA
**945**	Propionyl-CoA Carboxylase Subunit Beta	PCCB
**946**	Pro-Platelet Basic Protein	PPBP
**947**	Pro-Platelet Basic Protein Pseudogene 1	PPBPP1
**948**	Prosaposin	PSAP
**949**	Prostaglandin D2 Receptor	PTGDR
**950**	Prostaglandin D2 Receptor 2	PTGDR2
**951**	Prostaglandin D2 Synthase	PTGDS
**952**	Prostaglandin E Receptor 1	PTGER1
**953**	Prostaglandin E Receptor 2	PTGER2
**954**	Prostaglandin E Receptor 3	PTGER3
**955**	Prostaglandin E Receptor 4	PTGER4
**956**	Prostaglandin-Endoperoxide Synthase 1	PTGS1
**957**	Prostaglandin-Endoperoxide Synthase 2	PTGS2
**958**	Prostate Cancer Associated Transcript 4	PCAT4
**959**	Proteasome Subunit Beta 8	PSMB8
**960**	Proteasome Subunit Beta 9	PSMB9
**961**	Protein Activator Of Interferon Induced Protein Kinase EIF2AK2	PRKRA
**962**	Protein C, Inactivator Of Coagulation Factors Va And VIIIa	PROC
**963**	Protein Kinase C Alpha	PRKCA
**964**	Protein Kinase C Beta	PRKCB
**965**	Protein Kinase C Delta	PRKCD
**966**	Protein Kinase C Eta	PRKCH
**967**	Protein Kinase CAMP-Activated Catalytic Subunit Alpha	PRKACA
**968**	Protein Phosphatase 3 Catalytic Subunit Beta	PPP3CB
**969**	Protein Phosphatase, Mg2+/Mn2+ Dependent 1A	PPM1A
**970**	Protein S	PROS1
**971**	Protein Tyrosine Kinase 2	PTK2
**972**	Protein Tyrosine Phosphatase Non-Receptor Type 11	PTPN11
**973**	Protein Tyrosine Phosphatase Non-Receptor Type 22	PTPN22
**974**	Protein Tyrosine Phosphatase Non-Receptor Type 6	PTPN6
**975**	Protein Tyrosine Phosphatase Receptor Type C	PTPRC
**976**	Proteoglycan 2, Pro Eosinophil Major Basic Protein	PRG2
**977**	Protocadherin 1	PCDH1

**978**	Protocadherin 9	PCDH9
**979**	Pseudouridine Synthase 10	PUS10
**980**	Pvt1 Oncogene	PVT1
**981**	RAB GTPase Activating Protein 1	RABGAP1
**982**	RAB Guanine Nucleotide Exchange Factor 1	RABGEF1
**983**	Rac Family Small GTPase 1	RAC1
**984**	RAD50 Double Strand Break Repair Protein	RAD50
**985**	RAD51 Recombinase	RAD51
**986**	Rap Guanine Nucleotide Exchange Factor 3	RAPGEF3
**987**	RAR Related Orphan Receptor A	RORA
**988**	RAS Guanyl Releasing Protein 4	RASGRP4
**989**	RAS P21 Protein Activator 1	RASA1
**990**	RB Binding Protein 7, Chromatin Remodeling Factor	RBBP7
**991**	Recombination Activating 1	RAG1
**992**	Recombination Activating 2	RAG2
**993**	Regulator Of Cell Cycle	RGCC
**994**	REL Proto-Oncogene, NF-KB Subunit	REL
**995**	RELA Proto-Oncogene, NF-KB Subunit	RELA
**996**	RELB Proto-Oncogene, NF-KB Subunit	RELB
**997**	Repetin	RPTN
**998**	Retinoic Acid Receptor Responder 2	RARRES2
**999**	Retinoid X Receptor Alpha	RXRA
**1000**	Ribonuclease A Family Member 2	RNASE2
**1001**	Ribonuclease A Family Member 2 Pseudogene	ECRP
**1002**	Ribonuclease A Family Member 3	RNASE3
**1003**	Ribonuclease A Family Member 7	RNASE7
**1004**	Ribosomal Protein L17 Pseudogene 2	RPL17P2
**1005**	Ribosomal Protein L21 Pseudogene 119	RPL21P119
**1006**	Ribosomal Protein S14 Pseudogene 8	RPS14P8
**1007**	Ribosomal Protein S23	RPS23
**1008**	Ribosomal Protein S5 Pseudogene 5	RPS5P5
**1009**	Ring Finger Protein 113A	RNF113A
**1010**	Ring Finger Protein 39	RNF39
**1011**	RNA Polymerase III Subunit A	POLR3A
**1012**	RNA, U4atac Small Nuclear (U12-Dependent Splicing)	RNU4ATAC
**1013**	S100 Calcium Binding Protein A1	S100A1
**1014**	S100 Calcium Binding Protein A10	S100A10
**1015**	S100 Calcium Binding Protein A11	S100A11
**1016**	S100 Calcium Binding Protein A12	S100A12
**1017**	S100 Calcium Binding Protein A14	S100A14
**1018**	S100 Calcium Binding Protein A2	S100A2
**1019**	S100 Calcium Binding Protein A3	S100A3
**1020**	S100 Calcium Binding Protein A4	S100A4
**1021**	S100 Calcium Binding Protein A5	S100A5
**1022**	S100 Calcium Binding Protein A6	S100A6
**1023**	S100 Calcium Binding Protein A7	S100A7
**1024**	S100 Calcium Binding Protein A7A	S100A7A
**1025**	S100 Calcium Binding Protein A8	S100A8
**1026**	S100 Calcium Binding Protein A9	S100A9
**1027**	S100 Calcium Binding Protein B	S100B
**1028**	SATB Homeobox 1	SATB1
**1029**	SBDS Ribosome Maturation Factor	SBDS
**1030**	Secreted LY6/PLAUR Domain Containing 1	SLURP1
**1031**	Secreted Phosphoprotein 1	SPP1
**1032**	Secretoglobin Family 1A Member 1	SCGB1A1
**1033**	Secretoglobin Family 3A Member 2	SCGB3A2
**1034**	Secretory Leukocyte Peptidase Inhibitor	SLPI
**1035**	Selectin E	SELE

**1036**	Selectin L	SELL
**1037**	Selectin P	SELP
**1038**	Selectin P Ligand	SELPLG
**1039**	Semaphorin 3A	SEMA3A
**1040**	Semaphorin 4A	SEMA4A
**1041**	Semaphorin 4D	SEMA4D
**1042**	Semaphorin 6A	SEMA6A
**1043**	Sequestosome 1	SQSTM1
**1044**	Serine Palmitoyltransferase Long Chain Base Subunit 1	SPTLC1
**1045**	Serine Palmitoyltransferase Long Chain Base Subunit 2	SPTLC2
**1046**	Serine Peptidase Inhibitor, Kazal Type 5	SPINK5
**1047**	Serine Peptidase Inhibitor, Kazal Type 6	SPINK6
**1048**	Serine Peptidase Inhibitor, Kazal Type 9	SPINK9
**1049**	Serine Peptidase Inhibitor, Kunitz Type 2	SPINT2
**1050**	Serine/Threonine Kinase 4	STK4
**1051**	Serotonin Transporter Intronic VNTR Enhancer	STIN2-VNTR
**1052**	Serpin Family A Member 1	SERPINA1
**1053**	Serpin Family A Member 3	SERPINA3
**1054**	Serpin Family B Member 1	SERPINB1
**1055**	Serpin Family B Member 13	SERPINB13
**1056**	Serpin Family B Member 2	SERPINB2
**1057**	Serpin Family B Member 3	SERPINB3
**1058**	Serpin Family B Member 4	SERPINB4
**1059**	Serpin Family B Member 8	SERPINB8
**1060**	SET Domain Bifurcated Histone Lysine Methyltransferase 2	SETDB2
**1061**	SETDB2-PHF11 Readthrough	SETDB2-PHF11
**1062**	SH2B Adaptor Protein 3	SH2B3
**1063**	SH3 And PX Domains 2B	SH3PXD2B
**1064**	SHOC2 Leucine Rich Repeat Scaffold Protein	SHOC2
**1065**	Sialophorin	SPN
**1066**	Signal Recognition Particle 54	SRP54
**1067**	Signal Transducer And Activator Of Transcription 1	STAT1
**1068**	Signal Transducer And Activator Of Transcription 3	STAT3
**1069**	Signal Transducer And Activator Of Transcription 4	STAT4
**1070**	Signal Transducer And Activator Of Transcription 5A	STAT5A
**1071**	Signal Transducer And Activator Of Transcription 5B	STAT5B
**1072**	Signal Transducer And Activator Of Transcription 6	STAT6
**1073**	SIK Family Kinase 3	SIK3
**1074**	SMAD Family Member 2	SMAD2
**1075**	SMAD Family Member 3	SMAD3
**1076**	SMAD Family Member 7	SMAD7
**1077**	Small Nuclear Ribonucleoprotein Polypeptide N	SNRPN
**1078**	Small Nuclear Ribonucleoprotein U1 Subunit 70	SNRNP70
**1079**	Small Proline Rich Protein 1A	SPRR1A
**1080**	Small Proline Rich Protein 1B	SPRR1B
**1081**	Small Proline Rich Protein 3	SPRR3
**1082**	Sodium Channel Epithelial 1 Alpha Subunit	SCNN1A
**1083**	Sodium Voltage-Gated Channel Alpha Subunit 7	SCN7A
**1084**	Solute Carrier Family 11 Member 1	SLC11A1
**1085**	Solute Carrier Family 17 Member 5	SLC17A5
**1086**	Solute Carrier Family 2 Member 2	SLC2A2
**1087**	Solute Carrier Family 25 Member 17	SLC25A17
**1088**	Solute Carrier Family 25 Member 46	SLC25A46
**1089**	Solute Carrier Family 26 Member 2	SLC26A2
**1090**	Solute Carrier Family 29 Member 3	SLC29A3
**1091**	Solute Carrier Family 39 Member 13	SLC39A13
**1092**	Solute Carrier Family 4 Member 1 (Diego Blood Group)	SLC4A1
**1093**	Solute Carrier Family 6 Member 4	SLC6A4

**1094**	Solute Carrier Family 6 Member 4 Gene Promoter	LOC110806262
**1095**	Solute Carrier Family 7 Member 9	SLC7A9
**1096**	Solute Carrier Organic Anion Transporter Family Member 2A1	SLCO2A1
**1097**	Somatostatin	SST
**1098**	Somatostatin Receptor 5	SSTR5
**1099**	SOS Ras/Rac Guanine Nucleotide Exchange Factor 1	SOS1
**1100**	Sp1 Transcription Factor	SP1
**1101**	S-Phase Cyclin A Associated Protein In The ER	SCAPER
**1102**	Sphingomyelin Phosphodiesterase 1	SMPD1
**1103**	Sphingomyelin Phosphodiesterase 2	SMPD2
**1104**	Sphingosine-1-Phosphate Lyase 1	SGPL1
**1105**	Spi-1 Proto-Oncogene	SPI1
**1106**	Spleen Associated Tyrosine Kinase	SYK
**1107**	Spliceosome Associated Factor 1, Recruiter Of U4/U6.U5 Tri-SnRNP	SART1
**1108**	Sprouty Related EVH1 Domain Containing 1	SPRED1
**1109**	SRC Proto-Oncogene, Non-Receptor Tyrosine Kinase	SRC
**1110**	SRY-Box Transcription Factor 10	SOX10
**1111**	Ssemaphorin 4F	SEMA4F
**1112**	StAR Related Lipid Transfer Domain Containing 7	STARD7
**1113**	Steroid Sulfatase	STS
**1114**	Steroidogenic Acute Regulatory Protein Pseudogene 1	STARP1
**1115**	Stromal Interaction Molecule 1	STIM1
**1116**	Sulfotransferase Family 2B Member 1	SULT2B1
**1117**	Superoxide Dismutase 1	SOD1
**1118**	Superoxide Dismutase 2	SOD2
**1119**	Suppression Of Tumorigenicity 2	ST2
**1120**	Suppressor Of Cytokine Signaling 1	SOCS1
**1121**	Suppressor Of Cytokine Signaling 3	SOCS3
**1122**	Surfactant Protein D	SFTPD
**1123**	Syndecan 4	SDC4
**1124**	T Cell Immune Regulator 1, ATPase H+ Transporting V0 Subunit A3	TCIRG1
**1125**	T Cell Immunoglobulin And Mucin Domain Containing 4	TIMD4
**1126**	T Cell Immunoreceptor With Ig And ITIM Domains	TIGIT
**1127**	T Cell Receptor Beta Locus	TRB
**1128**	Tachykinin Precursor 1	TAC1
**1129**	Tachykinin Receptor 1	TACR1
**1130**	Tachykinin Receptor 2	TACR2
**1131**	TATA-Box Binding Protein Associated Factor 1	TAF1
**1132**	TBC1 Domain Family Member 4	TBC1D4
**1133**	T-Box Transcription Factor 21	TBX21
**1134**	T-Box Transcription Factor T	TBXT
**1135**	Teashirt Zinc Finger Homeobox 1	TSHZ1
**1136**	Tec Protein Tyrosine Kinase	TEC
**1137**	TEK Receptor Tyrosine Kinase	TEK
**1138**	Tenascin C	TNC
**1139**	Tenascin XB	TNXB
**1140**	Testis Expressed Basic Protein 1	TSBP1
**1141**	Tetraspanin 32	TSPAN32
**1142**	TGFB Induced Factor Homeobox 1	TGIF1
**1143**	Thiopurine S-Methyltransferase	TPMT
**1144**	Thioredoxin	TXN
**1145**	Thrombomodulin	THBD
**1146**	Thromboxane A2 Receptor	TBXA2R
**1147**	Thy-1 Cell Surface Antigen	THY1
**1148**	Thymic Stromal Lymphopoietin	TSLP
**1149**	Thymocyte Selection Associated	THEMIS
**1150**	Thymocyte Selection Associated High Mobility Group Box	TOX
**1151**	Thymopoietin	TMPO

**1152**	Tight Junction Protein 1	TJP1
**1153**	Timeless Circadian Regulator	TIMELESS
**1154**	TIMP Metallopeptidase Inhibitor 1	TIMP1
**1155**	TIMP Metallopeptidase Inhibitor 2	TIMP2
**1156**	TIMP Metallopeptidase Inhibitor 4	TIMP4
**1157**	TIR Domain Containing Adaptor Protein	TIRAP
**1158**	Titin	TTN
**1159**	TNF Receptor Associated Factor 6	TRAF6
**1160**	TNF Receptor Superfamily Member 11b	TNFRSF11B
**1161**	TNF Receptor Superfamily Member 14	TNFRSF14
**1162**	TNF Receptor Superfamily Member 18	TNFRSF18
**1163**	TNF Receptor Superfamily Member 1A	TNFRSF1A
**1164**	TNF Receptor Superfamily Member 1B	TNFRSF1B
**1165**	TNF Receptor Superfamily Member 4	TNFRSF4
**1166**	TNF Receptor Superfamily Member 8	TNFRSF8
**1167**	TNF Receptor Superfamily Member 9	TNFRSF9
**1168**	TNF Superfamily Member 11	TNFSF11
**1169**	TNF Superfamily Member 12	TNFSF12
**1170**	TNF Superfamily Member 13	TNFSF13
**1171**	TNF Superfamily Member 13b	TNFSF13B
**1172**	TNF Superfamily Member 14	TNFSF14
**1173**	TNF Superfamily Member 15	TNFSF15
**1174**	TNF Superfamily Member 18	TNFSF18
**1175**	TNF Superfamily Member 4	TNFSF4
**1176**	TNF Superfamily Member 8	TNFSF8
**1177**	TNFAIP3 Interacting Protein 1	TNIP1
**1178**	Toll Interacting Protein	TOLLIP
**1179**	Toll Like Receptor 1	TLR1
**1180**	Toll Like Receptor 10	TLR10
**1181**	Toll Like Receptor 2	TLR2
**1182**	Toll Like Receptor 3	TLR3
**1183**	Toll Like Receptor 4	TLR4
**1184**	Toll Like Receptor 5	TLR5
**1185**	Toll Like Receptor 6	TLR6
**1186**	Toll Like Receptor 7	TLR7
**1187**	Toll Like Receptor 8	TLR8
**1188**	Toll Like Receptor 9	TLR9
**1189**	TRAF3 Interacting Protein 2	TRAF3IP2
**1190**	Transcription Factor 7	TCF7
**1191**	Transcription Termination Factor 2	TTF2
**1192**	Transferrin Receptor	TFRC
**1193**	Transforming Growth Factor Alpha	TGFA
**1194**	Transforming Growth Factor Beta 1	TGFB1
**1195**	Transforming Growth Factor Beta 2	TGFB2
**1196**	Transforming Growth Factor Beta Receptor 3	TGFBR3
**1197**	Transglutaminase 1	TGM1
**1198**	Transglutaminase 2	TGM2
**1199**	Transglutaminase 3	TGM3
**1200**	Transglutaminase 5	TGM5
**1201**	Transient Receptor Potential Cation Channel Subfamily A Member 1	TRPA1
**1202**	Transient Receptor Potential Cation Channel Subfamily C Member 6	TRPC6
**1203**	Transient Receptor Potential Cation Channel Subfamily V Member 1	TRPV1
**1204**	Transient Receptor Potential Cation Channel Subfamily V Member 3	TRPV3
**1205**	Translocator Protein	TSPO
**1206**	Transmembrane Protein 108	TMEM108
**1207**	Transmembrane Protein 232	TMEM232
**1208**	Transmembrane Protein 79	TMEM79
**1209**	Transporter 1, ATP Binding Cassette Subfamily B Member	TAP1

**1210**	Transporter 2, ATP Binding Cassette Subfamily B Member	TAP2
**1211**	Tripartite Motif Containing 13	TRIM13
**1212**	Troponin T1, Slow Skeletal Type	TNNT1
**1213**	Tryptase Alpha/Beta 1	TPSAB1
**1214**	Tumor Necrosis Factor	TNF
**1215**	Tumor Protein P53	TP53
**1216**	Tumor Protein P63	TP63
**1217**	Tumor Protein P73	TP73
**1218**	Tumor Protein, Translationally-Controlled 1	TPT1
**1219**	TXK Tyrosine Kinase	TXK
**1220**	Tyrosinase Related Protein 1	TYRP1
**1221**	Tyrosine Hydroxylase	TH
**1222**	Tyrosine Kinase 2	TYK2
**1223**	Ubiquitin Associated And SH3 Domain Containing A	UBASH3A
**1224**	Ubiquitin C-Terminal Hydrolase L1	UCHL1
**1225**	Ubiquitin Specific Peptidase 8	USP8
**1226**	UDP Glucuronosyltransferase Family 1 Member A9	UGT1A9
**1227**	UDP-Glucose Ceramide Glucosyltransferase	UGCG
**1228**	Uncharacterized LOC105379193	LOC105379193
**1229**	Urocortin	UCN
**1230**	Urocortin 2	UCN2
**1231**	Urocortin 3	UCN3
**1232**	Uroporphyrinogen III Synthase	UROS
**1233**	Vanin 1	VNN1
**1234**	Vanin 2	VNN2
**1235**	Vanin 3	VNN3
**1236**	Vascular Cell Adhesion Molecule 1	VCAM1
**1237**	Vascular Endothelial Growth Factor A	VEGFA
**1238**	Vascular Endothelial Growth Factor C	VEGFC
**1239**	Vasoactive Intestinal Peptide	VIP
**1240**	Vimentin	VIM
**1241**	Vitamin D Receptor	VDR
**1242**	Vitronectin	VTN
**1243**	Voltage Dependent Anion Channel 2	VDAC2
**1244**	Von Willebrand Factor	VWF
**1245**	VPS50 Subunit Of EARP/GARPII Complex	VPS50
**1246**	V-Set And Transmembrane Domain Containing 1	VSTM1
**1247**	WASP Actin Nucleation Promoting Factor	WAS
**1248**	Xin Actin Binding Repeat Containing 2	XIRP2
**1249**	X-Linked Inhibitor Of Apoptosis	XIAP
**1250**	X-Ray Repair Cross Complementing 2	XRCC2
**1251**	Zeta Chain Of T Cell Receptor Associated Protein Kinase 70	ZAP70
**1252**	Zinc Finger And BTB Domain Containing 10	ZBTB10
**1253**	Zinc Finger Protein 341	ZNF341
**1254**	Zinc Finger Protein 365	ZNF365
**1255**	Zinc Finger Protein 750	ZNF750
**1256**	Zinc Metallopeptidase STE24	ZMPSTE24

Table S4. The gene ontology enrichment analysis for intersection targets between compound (OB ⩾ 30% and DL ⩾ 0.18) and AD related targets

	Term	Count	%	PValue	Genes	Fold Enrichment	Bonferroni	Benjamini	FDR
1	GO:0043066~negative regulation of apoptotic process	27	23.07692	4.84E-17	PPARD, XIAP, MCL1, MMP9, NFKBIA, TP63, BCL2L1, AKT1, CASP3, ALB, BCL2, CAT, NQO1, EGFR, IL4, IL6, RELA, CDKN1A, CD40LG, GSK3B, VEGFA, MDM2, HSPB1, MPO, MAPK8, GSTP1, IL2	8.51665258	8.92E-14	8.92E-14	8.25E-14
2	GO:0042493~response to drug	21	17.94872	2.25E-14	IL4, ICAM1, IL6, HSP90AA1, PTGS2, CYP1A1, RELA, PPARG, SLC6A4, SOD1, STAT1, POR, FOS, CASP3, CDKN1A, CCND1, JUN, BCL2, MDM2, CAT, COL1A1	9.91430499	4.15E-11	2.08E-11	3.84E-11
3	GO:0032496~response to lipopolysaccharide	17	14.52991	2.45E-14	OPRM1, PTGS2, CYP1A1, CXCL2, FASLG, CYP1A2, CXCL11, CXCL10, VCAM1, FOS, CASP3, THBD, JUN, CASP8, SLPI, MPO, SELE	14.8772149	4.52E-11	1.51E-11	4.18E-11
4	GO:0097192~extrinsic apoptotic signaling pathway in absence of ligand	10	8.547009	1.19E-12	IL4, CASP3, MCL1, BAX, BCL2, GSK3B, IL1B, BCL2L1, IL1A, IL2	42.2121669	2.19E-09	5.47E-10	2.02E-09
5	GO:0045471~response to ethanol	13	11.11111	7.53E-12	IL4, VCAM1, ICAM1, CCND1, CCL2, HMGCR, CASP8, CAT, SOD1, HTR3A, NQO1, GSTP1, IL2	17.7693122	1.39E-08	2.78E-09	1.28E-08
6	GO:0001666~response to hypoxia	15	12.82051	1.34E-11	PPARA, CCL2, CYP1A1, SLC6A4, MMP2, PRKCB, VCAM1, CASP3, HIF1A, HMOX1, VEGFA, CAT, NOS2, PLAU, DPP4	12.5163983	2.48E-08	4.13E-09	2.29E-08
7	GO:0009636~response to toxic substance	12	10.25641	1.47E-11	MAPK1, FOS, CDKN1A, BAX, BCL2, SLC6A4, MAPK3, PON1, MDM2, NQO1, GSTP1, AHR	20.2618401	2.70E-08	3.86E-09	2.50E-08
8	GO:0045944~positive regulation of transcription from RNA polymerase II promoter	30	25.64103	1.54E-11	PPARA, PPARG, NFKBIA, TP63, NR3C1, CXCL10, AKT1, FOS, IL1B, TOP2A, IL1A, EGFR, IL4, AR, IL6, RELA, RXRA, ESR1, STAT1, AHR, ADRB2, NR1I2, HIF1A, JUN, GSK3B, MAPK14, VEGFA, MAPK3, IRF1, IL2	4.38903265	2.84E-08	3.55E-09	2.62E-08
9	GO:0006915~apoptotic process	23	19.65812	4.06E-11	PPARD, XIAP, NFKBIA, GJA1, TP63, FASLG, NR3C1, STAT1, PRKCD, AHR, PRKCB, MAPK1, CASP3, CASP7, BCL2, BAX, MAPK14, CASP8, MAPK3, IRF1, IL1B, IGFBP3, IL1A	5.82185441	7.48E-08	8.31E-09	6.91E-08
10	GO:0007568~aging	14	11.96581	1.13E-10	IL6, CCL2, CYP1A1, HMGCR, RELA, SOD1, AKT1, VCAM1, FOS, CASP7, JUN, MPO, CAT, NQO1	12.1775706	2.09E-07	2.09E-08	1.93E-07
11	GO:0045893~positive regulation of transcription, DNA-templated	21	17.94872	3.44E-10	IL4, PPARA, AR, IL6, PPARD, RELA, PPARG, ESR1, TP63, STAT1, AHR, FOS, MAPK1, NR1I2, HIF1A, JUN, MAPK3, IRF1, IL1B, COL1A1, EGF	5.85232761	6.35E-07	5.77E-08	5.86E-07
12	GO:0045429~positive regulation of nitric oxide biosynthetic process	9	7.692308	4.82E-10	OPRM1, AKT1, EGFR, ICAM1, IL6, HSP90AA1, PTGS2, ESR1, IL1B	30.039356	8.87E-07	7.40E-08	8.20E-07
13	GO:0032355~response to estradiol	11	9.401709	6.71E-10	CASP3, CCND1, PTGS2, SLC6A4, CASP8, ESR1, CAT, COL1A1, CYP1A2, NQO1, GSTP1	17.3487367	1.24E-06	9.52E-08	1.14E-06
14	GO:0006954~inflammatory response	18	15.38462	9.56E-10	IL6, CCL2, PTGER3, PTGS2, RELA, CRP, PTGS1, CXCL2, CXCL11, CXCL10, AKT1, FOS, IL10RB, CD40LG, IL1B, SELE, IL1A, SPP1	6.81631825	1.76E-06	1.26E-07	1.63E-06
15	GO:0071260~cellular response to mechanical stimulus	10	8.547009	1.36E-09	AKT1, EGFR, PTGS2, MAPK3, CASP8, IRF1, IL1B, GJA1, MAPK8, COL1A1	20.2142771	2.51E-06	1.67E-07	2.32E-06
16	GO:0046677~response to antibiotic	8	6.837607	1.82E-09	CASP3, IL6, HSP90AA1, CCL2, CYP1A1, CASP8, MDM2, SOD1	35.8803419	3.36E-06	2.10E-07	3.11E-06
17	GO:0031663~lipopolysaccharide-mediated signaling pathway	8	6.837607	1.82E-09	AKT1, MAPK1, CCL2, MAPK14, MAPK3, NFKBIA, IL1B, NOS3	35.8803419	3.36E-06	2.10E-07	3.11E-06
18	GO:0010628~positive regulation of gene expression	15	12.82051	3.51E-09	AR, IL6, PPARD, ERBB2, CRP, SLC6A4, GJA1, NR1I2, HIF1A, MAPK14, VEGFA, IL1B, MDM2, MAPK8, IL1A	8.21687219	6.48E-06	3.81E-07	5.98E-06
19	GO:0007165~signal transduction	29	24.78632	3.83E-09	CCL2, CLDN4, ERBB2, PPARG, TNFSF15, FASLG, GJA1, NR3C1, CXCL11, CXCL10, AKT1, IL10RB, IL1B, HTR3A, EGF, RASA1, IFNGR1, EGFR, AR, HSP90AA1, ESR1, PRKCD, PRKCB, MAPK1, THBD, HIF1A, NR1I2, MAPK14, PLAU	3.58494372	7.06E-06	3.92E-07	6.52E-06
20	GO:0009409~response to cold	8	6.837607	4.42E-09	FOS, IL6, ADRB2, HSP90AA1, ADRB1, CASP8, PPARG, CXCL10	31.8936372	8.15E-06	4.29E-07	7.53E-06
21	GO:0007566~embryo implantation	8	6.837607	1.38E-08	PPARD, PTGS2, RXRA, MMP9, IL1B, SOD1, MMP2, SPP1	27.3374033	2.55E-05	1.27E-06	2.35E-05
22	GO:0051090~regulation of sequence-specific DNA binding transcription factor activity	7	5.982906	1.52E-08	MAPK1, FOS, MAPK14, JUN, HMOX1, MAPK3, MAPK8	40.1859829	2.80E-05	1.33E-06	2.58E-05
23	GO:0008284~positive regulation of cell proliferation	18	15.38462	2.10E-08	EGFR, ODC1, AR, IL6, RELA, FASLG, IGF2, BCL2L1, CXCL10, AKR1C3, MAPK1, BCL2, VEGFA, MDM2, HAS2, EGF, DPP4, IL2	5.54374381	3.86E-05	1.76E-06	3.57E-05
24	GO:0007584~response to nutrient	9	7.692308	4.19E-08	AKR1C3, IL4, VCAM1, HMGCR, SLC6A4, PPARG, STAT1, NQO1, POR	17.4553015	7.72E-05	3.36E-06	7.14E-05
25	GO:0042542~response to hydrogen peroxide	8	6.837607	5.63E-08	CASP3, JUN, HMOX1, BCL2, CAT, COL1A1, SOD1, STAT1	22.5131557	1.04E-04	4.33E-06	9.59E-05
26	GO:0071222~cellular response to lipopolysaccharide	10	8.547009	8.57E-08	ICAM1, PPARD, IL6, CCL2, MAPK14, RELA, MAPK8, NOS2, GSTP1, CXCL10	12.701006	1.58E-04	6.32E-06	1.46E-04
27	GO:0055114~oxidation-reduction process	19	16.23932	1.23E-07	CYP3A4, PTGS2, CYP1A1, HMGCR, MAOA, PTGS1, CYP1A2, SOD1, POR, AKR1C3, VCAM1, GSR, FASN, MPO, NOS3, ALOX5, NOS2, NQO1, CYP19A1	4.60626011	2.27E-04	8.74E-06	2.10E-04
28	GO:0071407~cellular response to organic cyclic compound	8	6.837607	1.59E-07	AKT1, CASP3, CCL2, CYP1A1, CASP8, NFKBIA, IL1B, STAT1	19.4605244	2.92E-04	1.08E-05	2.70E-04
29	GO:0048661~positive regulation of smooth muscle cell proliferation	8	6.837607	1.79E-07	AKT1, EGFR, IL6, PTGS2, HMGCR, JUN, HMOX1, STAT1	19.1361823	3.29E-04	1.18E-05	3.04E-04
30	GO:0018105~peptidyl-serine phosphorylation	10	8.547009	2.05E-07	PRKCA, AKT1, MAPK1, MAPK14, BCL2, GSK3B, MAPK3, MAPK8, PRKCD, PRKCB	11.4817094	3.78E-04	1.30E-05	3.49E-04
31	GO:0032930~positive regulation of superoxide anion generation	5	4.273504	2.65E-07	EGFR, CRP, SOD1, PRKCD, GSTP1	79.7340931	4.89E-04	1.63E-05	4.52E-04

**32**	GO:0071456~cellular response to hypoxia	9	7.692308	3.25E-07	AKT1, ICAM1, PPARD, HIF1A, PTGS2, HMOX1, BCL2, VEGFA, MDM2	13.4551282	5.99E-04	1.93E-05	5.53E-04
**33**	GO:0006805~xenobiotic metabolic process	8	6.837607	1.10E-06	CYP3A4, NR1I2, PTGS1, CYP1A2, NQO1, GSTP1, AHR, POR	14.7201403	0.0020297	6.35E-05	0.001877
**34**	GO:0045766~positive regulation of angiogenesis	9	7.692308	1.30E-06	PRKCA, HIF1A, HMOX1, VEGFA, HSPB1, IL1B, NOS3, IL1A, PRKCB	11.232107	0.0023939	7.26E-05	0.002215
**35**	GO:0030168~platelet activation	9	7.692308	1.30E-06	PRKCA, AKT1, MAPK1, IL6, CD40LG, MAPK3, COL1A1, PRKCD, PRKCB	11.232107	0.0023939	7.26E-05	0.002215
**36**	GO:0034097~response to cytokine	7	5.982906	1.50E-06	IL4, FOS, MCL1, JUN, BCL2, BCL2L1, STAT1	19.3201841	0.0027591	8.13E-05	0.002553
**37**	GO:0045909~positive regulation of vasodilation	6	5.128205	1.50E-06	EGFR, PPARD, HMOX1, GJA1, NOS3, NOS2	29.694076	0.0027616	7.90E-05	0.002555
**38**	GO:0043200~response to amino acid	6	5.128205	2.12E-06	ICAM1, CASP3, IL6, CCL2, RELA, GSTP1	27.7783292	0.0039057	1.09E-04	0.003616
**39**	GO:0045907~positive regulation of vasoconstriction	6	5.128205	2.50E-06	AKT1, EGFR, ICAM1, PTGS2, ADRA1B, GJA1	26.9102564	0.0046019	1.25E-04	0.004262
**40**	GO:0001934~positive regulation of protein phosphorylation	9	7.692308	2.75E-06	AKT1, EGFR, CCND1, MMP9, ERBB2, MAPK3, VEGFA, IL1B, IGF2	10.1708056	0.005058	1.33E-04	0.004685
**41**	GO:0001525~angiogenesis	11	9.401709	3.33E-06	PRKCA, HIF1A, CCL2, PTGS2, MAPK14, JUN, HMOX1, VEGFA, NOS3, EGF, MMP2	7.07952934	0.0061256	1.58E-04	0.005677
**42**	GO:0070374~positive regulation of ERK1 and ERK2 cascade	10	8.547009	3.46E-06	OPRM1, PRKCA, EGFR, ICAM1, IL6, CCL2, HMGCR, JUN, MAPK3, VEGFA	8.201221	0.0063568	1.59E-04	0.005892
**43**	GO:0035994~response to muscle stretch	5	4.273504	3.69E-06	FOS, MAPK14, RELA, JUN, NFKBIA	44.8504274	0.0067787	1.66E-04	0.006285
**44**	GO:0051092~positive regulation of NF-kappaB transcription factor activity	9	7.692308	3.89E-06	ICAM1, AR, IL6, CD40LG, RELA, NFKBIA, IL1B, CAT, PRKCB	9.71197224	0.0071366	1.71E-04	0.006618
**45**	GO:0060749~mammary gland alveolus development	5	4.273504	4.80E-06	AR, CCND1, VEGFA, ESR1, EGF	42.2121669	0.0088082	2.06E-04	0.008174
**46**	GO:0030574~collagen catabolic process	7	5.982906	5.16E-06	MMP10, MMP9, CTSD, COL1A1, MMP3, MMP2, MMP1	15.6976496	0.0094672	2.16E-04	0.008789
**47**	GO:2001240~negative regulation of extrinsic apoptotic signaling pathway in absence of ligand	6	5.128205	5.27E-06	AKT1, MCL1, BCL2, IL1B, BCL2L1, IL1A	23.2737353	0.009666	2.16E-04	0.008974
**48**	GO:0043627~response to estrogen	7	5.982906	5.65E-06	MAPK1, CCND1, HSP90AA1, HMOX1, PPARG, ESR1, CA2	15.4561473	0.0103669	2.27E-04	0.009628
**49**	GO:0018107~peptidyl-threonine phosphorylation	6	5.128205	6.04E-06	AKT1, MAPK1, BCL2, GSK3B, MAPK8, PRKCD	22.6612686	0.0110617	2.37E-04	0.010277
**50**	GO:0006955~immune response	14	11.96581	6.81E-06	IL4, IL6, CCL2, CXCL2, TNFSF15, FASLG, CXCL11, CXCL10, IL10RB, CD40LG, IL1B, SLPI,	4.77268206	0.0124691	2.61E-04	0.011593
**51**	GO:0043536~positive regulation of blood vessel endothelial cell migration	5	4.273504	7.73E-06	PRKCA, AKT1, MAPK14, VEGFA, HSPB1	37.7687809	0.0141542	2.91E-04	0.013171
**52**	GO:0001541~ovarian follicle development	6	5.128205	1.00E-05	ICAM1, BAX, BCL2, VEGFA, BCL2L1, SOD1	20.5030525	0.0182718	3.69E-04	0.017038
**53**	GO:0010165~response to X-ray	5	4.273504	1.44E-05	CDKN1A, CASP3, CCND1, THBD, TP63	32.6184926	0.0261288	5.19E-04	0.024461
**54**	GO:0051591~response to cAMP	6	5.128205	1.58E-05	FOS, THBD, RELA, JUN, COL1A1, STAT1	18.7201784	0.0286501	5.59E-04	0.026855
**55**	GO:0035924~cellular response to vascular endothelial growth factor stimulus	5	4.273504	1.73E-05	VCAM1, AKT1, MAPK14, VEGFA, HSPB1	31.2002973	0.0313769	6.01E-04	0.029452
**56**	GO:0042060~wound healing	7	5.982906	1.89E-05	OPRM1, EGFR, PPARA, CASP3, PPARD, IL6,	12.5581197	0.0342544	6.45E-04	0.0322
**57**	GO:0009408~response to heat	6	5.128205	1.95E-05	AKT1, IL6, HSP90AA1, CCL2, NOS3, SOD1	17.9401709	0.0352771	6.53E-04	0.033179
**58**	GO:0006935~chemotaxis	8	6.837607	2.18E-05	IL4, MAPK1, CCL2, MAPK14, CXCL2, CXCL11, PLAU, CXCL10	9.41123721	0.0393368	7.16E-04	0.037074
**59**	GO:0050999~regulation of nitric-oxide synthase activity	5	4.273504	2.87E-05	AKT1, EGFR, HSP90AA1, IL1B, NOS3	27.600263	0.0515906	9.29E-04	0.048931
**60**	GO:0043524~negative regulation of neuron apoptotic process	8	6.837607	3.62E-05	CCL2, JUN, HMOX1, BAX, BCL2, BCL2L1, SOD1, RASA1	8.6982647	0.0645399	0.00115	0.061626
**61**	GO:0042127~regulation of cell proliferation	9	7.692308	4.30E-05	XIAP, JUN, CXCL2, PTGS1, NFKBIA, NOS2, CXCL11, PLAU, CXCL10	6.98212058	0.076212	0.001343	0.07322
**62**	GO:0043065~positive regulation of apoptotic process	11	9.401709	4.38E-05	AKT1, IL6, PTGS2, HMOX1, BAX, FASLG, MAPK8, BCL2L1, SOD1, IGFBP3, TOP2A	5.26245014	0.0775785	0.001345	0.074587
**63**	GO:0043401~steroid hormone mediated signaling pathway	6	5.128205	4.54E-05	PPARA, PPARD, NR1I2, RXRA, PPARG, ESR1	15.1075124	0.0801863	0.001369	0.077201
**64**	GO:0032091~negative regulation of protein binding	6	5.128205	4.54E-05	PPARA, BAX, GSK3B, SLPI, MAPK8, PRKCD	15.1075124	0.0801863	0.001369	0.077201
**65**	GO:0010332~response to gamma radiation	5	4.273504	5.89E-05	CCL2, BAX, BCL2, TP63, CXCL10	23.1486077	0.1028692	0.001749	0.100252
**66**	GO:0007267~cell-cell signaling	10	8.547009	6.71E-05	AR, ADRB2, ADRB1, ADRA1B, IL1B, GJA1, FASLG, CXCL11, CXCL10, IL2	5.65044754	0.1163596	0.001962	0.114236
**67**	GO:0043950~positive regulation of cAMP-mediated signaling	4	3.418803	6.75E-05	OPRM1, ADRB1, CXCL11, CXCL10	47.8404558	0.117028	0.001943	0.114935
**68**	GO:0019430~removal of superoxide radicals	4	3.418803	6.75E-05	MPO, NOS3, SOD1, NQO1	47.8404558	0.117028	0.001943	0.114935
**69**	GO:1902042~negative regulation of extrinsic apoptotic signaling pathway via death domain receptors	5	4.273504	7.58E-05	ICAM1, HMOX1, CASP8, FASLG, NOS3	21.7456617	0.1303625	0.002147	0.128978
**70**	GO:0051402~neuron apoptotic process	5	4.273504	7.58E-05	CASP7, BAX, BCL2, TP63, BCL2L1	21.7456617	0.1303625	0.002147	0.128978
**71**	GO:0071364~cellular response to epidermal growth factor stimulus	5	4.273504	7.58E-05	AKT1, EGFR, ERBB2, COL1A1, GSTP1	21.7456617	0.1303625	0.002147	0.128978
**72**	GO:0001649~osteoblast differentiation	7	5.982906	8.35E-05	AKT1, FASN, GJA1, CAT, COL1A1, IGFBP3, SPP1	9.66009204	0.1426243	0.002329	0.142081
**73**	GO:0008217~regulation of blood pressure	6	5.128205	8.56E-05	PTGS2, HMOX1, PTGS1, PPARG, NOS3, SOD1	13.2481262	0.1459823	0.002353	0.145702
**74**	GO:0006809~nitric oxide biosynthetic process	4	3.418803	8.73E-05	AKT1, NOS3, NOS2, NQO1	44.1604208	0.1486972	0.002365	0.148639
**75**	GO:0010745~negative regulation of macrophage derived foam cell differentiation	4	3.418803	8.73E-05	PPARA, CRP, PPARG, NFKBIA	44.1604208	0.1486972	0.002365	0.148639

**76**	GO:0006367~transcription initiation from RNA polymerase II	8	6.837607	8.87E-05	PPARA, AR, PPARD, NR1I2, RXRA, PPARG, ESR1, NR3C1	7.55375619	0.150771	0.002366	0.15089
**77**	GO:0006974~cellular response to DNA damage stimulus	9	7.692308	9.80E-05	AKT1, MAPK1, CDKN1A, CCND1, XIAP, BCL2, MAPK3, TP63, TOP2A	6.21005917	0.1652308	0.002577	0.166733
**78**	GO:0071356~cellular response to tumor necrosis factor	7	5.982906	1.14E-04	VCAM1, ICAM1, IL6, CCL2, RELA, HAS2, COL1A1	9.13317793	0.1894519	0.002954	0.19389
**79**	GO:0043154~negative regulation of cysteine-type endopeptidase activity involved in apoptotic process	6	5.128205	1.14E-04	AKT1, IL6, XIAP, VEGFA, MDM2, POR	12.4801189	0.1894575	0.002913	0.193896
**80**	GO:0001938~positive regulation of endothelial cell proliferation	6	5.128205	1.14E-04	PRKCA, AKT1, HIF1A, CCL2, JUN, VEGFA	12.4801189	0.1894575	0.002913	0.193896
**81**	GO:0098869~cellular oxidant detoxification	6	5.128205	1.22E-04	GSR, PTGS2, ALB, PTGS1, CAT, GSTP1	12.3018315	0.2014621	0.003077	0.207656
**82**	GO:0071347~cellular response to interleukin-1	6	5.128205	1.31E-04	ICAM1, IL6, HIF1A, CCL2, RELA, HAS2	12.1285663	0.2139175	0.003247	0.22215
**83**	GO:0060333~interferon-gamma-mediated signaling pathway	6	5.128205	1.31E-04	VCAM1, ICAM1, IRF1, STAT1, PRKCD, IFNGR1	12.1285663	0.2139175	0.003247	0.22215
**84**	GO:0097190~apoptotic signaling pathway	6	5.128205	1.31E-04	PRKCA, CASP3, PPARD, BAX, CASP8, FASLG	12.1285663	0.2139175	0.003247	0.22215
**85**	GO:0038128~ERBB2 signaling pathway	5	4.273504	1.33E-04	AKT1, EGFR, HSP90AA1, ERBB2, EGF	18.8843905	0.2175737	0.003266	0.226448
**86**	GO:0030522~intracellular receptor signaling pathway	5	4.273504	1.33E-04	PPARA, AR, PPARD, NR1I2, AHR	18.8843905	0.2175737	0.003266	0.226448
**87**	GO:0070141~response to UV-A	3	2.564103	1.41E-04	AKT1, EGFR, CCND1	143.521368	0.2292766	0.003421	0.24034
**88**	GO:0031281~positive regulation of cyclase activity	3	2.564103	1.41E-04	MAPK14, MAPK3, MAPK8	143.521368	0.2292766	0.003421	0.24034
**89**	GO:0030949~positive regulation of vascular endothelial growth factor receptor signaling pathway	4	3.418803	1.68E-04	HIF1A, VEGFA, IL1B, PRKCB	35.8803419	0.2669184	0.004024	0.286485
**90**	GO:0097194~execution phase of apoptosis	4	3.418803	1.68E-04	AKT1, CASP3, CASP7, CASP8	35.8803419	0.2669184	0.004024	0.286485
**91**	GO:0007623~circadian rhythm	6	5.128205	1.69E-04	EGFR, CLDN4, JUN, GSK3B, SLC6A4, NOS2	11.4817094	0.2681297	0.003994	0.288009
**92**	GO:0006508~proteolysis	13	11.11111	1.77E-04	MMP9, MMP3, MMP2, MMP1, MMP10, CASP3, CASP7, BACE1, CASP8, CTSD, LTA4H, PLAU, DPP4	3.73155556	0.2779449	0.004114	0.300447
**93**	GO:0022617~extracellular matrix disassembly	6	5.128205	1.80E-04	MMP10, MMP9, MMP3, MMP2, MMP1, SPP1	11.3306343	0.2827351	0.004145	0.306579
**94**	GO:0055093~response to	4	3.418803	2.04E-04	CDKN1A, CYP1A1, CAT, COL1A1	33.7697335	0.3128098	0.004621	0.346028
**95**	GO:0071276~cellular response to cadmium ion	4	3.418803	2.04E-04	AKR1C3, HMOX1, CYP1A2, SOD1	33.7697335	0.3128098	0.004621	0.346028
**96**	GO:0014066~regulation of phosphatidylinositol 3-kinase signaling	6	5.128205	2.04E-04	AKT1, EGFR, MAPK1, ERBB2, MAPK3, EGF	11.0401052	0.3131189	0.00457	0.346442
**97**	GO:0043525~positive regulation of neuron apoptotic process	5	4.273504	2.17E-04	CASP3, JUN, BAX, FASLG, NQO1	16.6885311	0.3293186	0.004801	0.368416
**98**	GO:0008283~cell proliferation	11	9.401709	2.25E-04	AKT1, EGFR, AR, ACHE, PPARD, CYP1A1, BCL2, ERBB2, ADRA1B, BCL2L1, IL1A	4.31348372	0.3395256	0.004926	0.382533
**99**	GO:0033189~response to vitamin A	4	3.418803	2.43E-04	PPARD, CYP1A1, PPARG, CAT	31.8936372	0.3610434	0.005256	0.413015
**100**	GO:0043410~positive regulation of MAPK cascade	6	5.128205	2.43E-04	AR, IL6, ADRB2, ADRA1B, IGF2, IGFBP3	10.6312124	0.3613639	0.005201	0.413477
**101**	GO:0006919~activation of cysteine-type endopeptidase activity involved in apoptotic process	6	5.128205	2.73E-04	CASP3, BAX, CASP8, PPARG, TNFSF15, FASLG	10.3750386	0.3950576	0.005761	0.463339
**102**	GO:0008637~apoptotic mitochondrial changes	4	3.418803	2.87E-04	AKT1, MCL1, BAX, BCL2L1	30.2150247	0.4109487	0.005996	0.487818
**103**	GO:0008630~intrinsic apoptotic signaling pathway in response to DNA damage	5	4.273504	3.07E-04	MCL1, HMOX1, BAX, BCL2, BCL2L1	15.2682306	0.4317704	0.006331	0.520903
**104**	GO:0051781~positive regulation of cell division	5	4.273504	3.07E-04	VEGFA, IL1B, IGF2, CAT, IL1A	15.2682306	0.4317704	0.006331	0.520903
**105**	GO:0006468~protein phosphorylation	12	10.25641	3.18E-04	PRKCA, AKT1, MAPK1, CCND1, CCL2, GSK3B, ERBB2, MAPK3, MAPK8, IGFBP3, PRKCD, PRKCB	3.77687809	0.4438512	0.006498	0.540654
**106**	GO:1902895~positive regulation of pri-miRNA transcription from RNA polymerase II promoter	4	3.418803	3.36E-04	FOS, HIF1A, RELA, JUN	28.7042735	0.4617998	0.006785	0.570797
**107**	GO:0006921~cellular component disassembly involved in execution phase of apoptosis	4	3.418803	3.90E-04	CASP3, CASP7, CASP8, PRKCD	27.3374033	0.5128425	0.007787	0.662298
**108**	GO:0032094~response to food	4	3.418803	3.90E-04	OPRM1, AKT1, CYP1A1, MPO	27.3374033	0.5128425	0.007787	0.662298
**109**	GO:0035902~response to immobilization stress	4	3.418803	3.90E-04	FOS, CYP1A1, PPARG, CYP1A2	27.3374033	0.5128425	0.007787	0.662298
**110**	GO:0008285~negative regulation of cell proliferation	11	9.401709	4.20E-04	OPRM1, AR, CDKN1A, IL6, PTGS2, JUN, IRF1, IL1B, NOS3, IGFBP3, IL1A	3.98670465	0.5392215	0.008297	0.713382
**111**	GO:2001243~negative regulation of intrinsic apoptotic signaling pathway	4	3.418803	4.49E-04	MCL1, BCL2, MMP9, BCL2L1	26.0947941	0.5633241	0.008776	0.762658
**112**	GO:0031622~positive regulation of fever generation	3	2.564103	4.67E-04	PTGER3, PTGS2, IL1B	86.1128205	0.5770033	0.009016	0.791837
**113**	GO:0000165~MAPK cascade	9	7.692308	4.70E-04	EGFR, MAPK1, CCL2, ERBB2, MAPK3, IL1B, EGF, RASA1, IL2	4.93012331	0.5792408	0.008977	0.796699
**114**	GO:0001836~release of cytochrome c from mitochondria	4	3.418803	5.14E-04	JUN, BAX, BCL2, BCL2L1	24.9602378	0.6125221	0.009727	0.872202
**115**	GO:0007159~leukocyte cell-cell adhesion	4	3.418803	6.61E-04	VCAM1, ICAM1, CD40LG, SELE	22.9634188	0.7044918	0.012362	1.120073
**116**	GO:0034612~response to tumor necrosis factor	4	3.418803	6.61E-04	CASP3, PTGS2, CASP8, SELE	22.9634188	0.7044918	0.012362	1.120073

**117**	GO:0042981~regulation of apoptotic process	8	6.837607	6.94E-04	MCL1, BAX, CASP8, ESR1, TP63, BCL2L1, STAT1, IGFBP3	5.3904739	0.7219023	0.012844	1.175537
**118**	GO:0009612~response to mechanical stimulus	5	4.273504	7.34E-04	CCL2, JUN, PPARG, MPO, STAT1	12.1628278	0.7416965	0.013445	1.242937
**119**	GO:0045840~positive regulation of mitotic nuclear division	4	3.418803	7.44E-04	IL1B, IGF2, EGF, IL1A	22.0802104	0.7462008	0.013485	1.258988
**120**	GO:0042102~positive regulation of T cell proliferation	5	4.273504	7.82E-04	IL4, VCAM1, IL6, CD40LG, IL1B	11.960114	0.7637062	0.014044	1.32417
**121**	GO:0051091~positive regulation of sequence-specific DNA binding transcription factor activity	6	5.128205	8.04E-04	IL4, AKT1, IL6, PPARG, ESR1, IL1B	8.201221	0.7729345	0.01429	1.360485
**122**	GO:0010575~positive regulation of vascular endothelial growth factor production	4	3.418803	8.33E-04	HIF1A, PTGS2, IL1B, IL1A	21.2624248	0.784533	0.014651	1.40826
**123**	GO:0000187~activation of MAPK activity	6	5.128205	8.76E-04	MAPK1, MAPK14, MAPK3, IL1B, SOD1, EGF	8.04792715	0.8010121	0.015259	1.480714
**124**	GO:0043123~positive regulation of I-kappaB kinase/NF-kappaB signaling	7	5.982906	8.81E-04	RELA, HMOX1, CASP8, GJA1, FASLG, IL1A, PRKCB	6.24005946	0.8030826	0.015213	1.490235
**125**	GO:0051881~regulation of mitochondrial membrane potential	4	3.418803	9.28E-04	BAX, BCL2, BCL2L1, SOD1	20.5030525	0.8192438	0.01586	1.56815
**126**	GO:0050727~regulation of inflammatory response	5	4.273504	9.41E-04	XIAP, PTGS2, RELA, ESR1, SELE	11.3905847	0.8235381	0.015933	1.590016
**127**	GO:0034351~negative regulation of glial cell apoptotic process	3	2.564103	9.71E-04	PRKCA, CCL2, PRKCD	61.5091575	0.8332038	0.016297	1.641225
**128**	GO:0071312~cellular response to alkaloid	3	2.564103	9.71E-04	ICAM1, MDM2, BCL2L1	61.5091575	0.8332038	0.016297	1.641225
**129**	GO:0006979~response to oxidative stress	6	5.128205	9.92E-04	AKT1, EGFR, PTGS2, HMOX1, PTGS1, MPO	7.82843823	0.8393751	0.016487	1.675483
**130**	GO:0051607~defense response to virus	7	5.982906	0.001001	IL6, IL10RB, RELA, BCL2, IRF1, STAT1, CXCL10	6.08878529	0.8420834	0.01649	1.69093
**131**	GO:0097421~liver regeneration	4	3.418803	0.00103	EGFR, CCND1, HMOX1, NFKBIA	19.7960507	0.8502099	0.016808	1.738909
**132**	GO:0046627~negative regulation of insulin receptor signaling pathway	4	3.418803	0.00103	RELA, IL1B, PRKCD, PRKCB	19.7960507	0.8502099	0.016808	1.738909
**133**	GO:0048662~negative regulation of smooth muscle cell proliferation	4	3.418803	0.00103	PPARD, HMOX1, PPARG, IGFBP3	19.7960507	0.8502099	0.016808	1.738909
**134**	GO:0060326~cell chemotaxis	5	4.273504	0.001058	VCAM1, CCL2, CXCL2, PRKCD, CXCL10	11.0401052	0.8578649	0.017117	1.786525
**135**	GO:1900740~positive regulation of protein insertion into mitochondrial membrane involved in apoptotic signaling pathway	4	3.418803	0.001138	BCL2, CASP8, TP63, MAPK8	19.1361823	0.8774229	0.018244	1.920774
**136**	GO:0008203~cholesterol metabolic process	5	4.273504	0.001253	IL4, PPARD, RXRA, PON1, CAT	10.5530417	0.9007415	0.019887	2.111816
**137**	GO:0002674~negative regulation of acute inflammatory response	3	2.564103	0.001289	IL4, PPARG, GSTP1	53.8205128	0.9072246	0.020288	2.17289
**138**	GO:0032966~negative regulation of collagen biosynthetic process	3	2.564103	0.001289	PPARD, IL6, PPARG	53.8205128	0.9072246	0.020288	2.17289
**139**	GO:0010888~negative regulation of lipid storage	3	2.564103	0.001289	IL6, CRP, NFKBIA	53.8205128	0.9072246	0.020288	2.17289
**140**	GO:0035234~ectopic germ cell programmed cell death	3	2.564103	0.001289	BAX, IL1B, IL1A	53.8205128	0.9072246	0.020288	2.17289
**141**	GO:0032872~regulation of stress-activated MAPK cascade	3	2.564103	0.001289	MAPK1, MAPK3, GSTP1	53.8205128	0.9072246	0.020288	2.17289
**142**	GO:0042220~response to cocaine	4	3.418803	0.001377	OPRM1, HSP90AA1, MDM2, HTR3A	17.9401709	0.9210549	0.021467	2.318698
**143**	GO:0033138~positive regulation of peptidyl-serine phosphorylation	5	4.273504	0.001395	AKT1, IL6, BCL2, GSK3B, VEGFA	10.2515263	0.9237414	0.021574	2.349942
**144**	GO:0038095~Fc-epsilon receptor signaling pathway	7	5.982906	0.001478	MAPK1, FOS, RELA, JUN, MAPK3, NFKBIA, MAPK8	5.64409872	0.9344833	0.022643	2.486833
**145**	GO:0048010~vascular endothelial growth factor receptor signaling pathway	5	4.273504	0.001549	HSP90AA1, CCL2, MAPK14, VEGFA, HSPB1	9.96676163	0.9425911	0.023532	2.605789
**146**	GO:0050900~leukocyte migration	6	5.128205	0.001573	ICAM1, THBD, MMP9, COL1A1, SELE, MMP1	7.05842791	0.9450777	0.023697	2.645629
**147**	GO:0034405~response to fluid shear stress	3	2.564103	0.00165	AKT1, GJA1, NOS3	47.8404558	0.9523388	0.024639	2.773102
**148**	GO:0008635~activation of cysteine-type endopeptidase activity involved in apoptotic process by cytochrome c	3	2.564103	0.00165	CASP3, CASP7, BAX	47.8404558	0.9523388	0.024639	2.773102
**149**	GO:0050665~hydrogen peroxide biosynthetic process	3	2.564103	0.00165	CYP1A1, CYP1A2, SOD1	47.8404558	0.9523388	0.024639	2.773102
**150**	GO:0030335~positive regulation of cell migration	7	5.982906	0.001749	PRKCA, EGFR, MAPK1, VEGFA, HAS2, COL1A1, PLAU	5.46005203	0.9602735	0.025885	2.936556
**151**	GO:0035556~intracellular signal transduction	10	8.547009	0.001922	PRKCA, AKT1, MAPK14, HMOX1, GSK3B, ADRA1B, HSPB1, PRKCD, RASA1, PRKCB	3.56132426	0.9711534	0.02819	3.223147
**152**	GO:0043407~negative regulation of MAP kinase activity	4	3.418803	0.001942	HMGCR, IL1B, PRKCD, GSTP1	15.9468186	0.972218	0.02826	3.256768
**153**	GO:0010507~negative regulation of autophagy	4	3.418803	0.001942	AKT1, MCL1, BCL2, BCL2L1	15.9468186	0.972218	0.02826	3.256768
**154**	GO:0032869~cellular response to insulin stimulus	5	4.273504	0.001985	AKT1, CCL2, PPARG, STAT1, GSTP1	9.31956932	0.9743104	0.028643	3.326736
**155**	GO:0045080~positive regulation of chemokine biosynthetic process	3	2.564103	0.002053	IL4, HMOX1, IL1B	43.0564103	0.9773626	0.029388	3.439652
**156**	GO:0019371~cyclooxygenase pathway	3	2.564103	0.002053	AKR1C3, PTGS2, PTGS1	43.0564103	0.9773626	0.029388	3.439652
**157**	GO:0035094~response to nicotine	4	3.418803	0.002103	VCAM1, CASP3, HMOX1, BCL2	15.5158235	0.9793568	0.02986	3.521891
**158**	GO:0034605~cellular response to heat	4	3.418803	0.002103	CDKN1A, HMOX1, IL1A, CXCL10	15.5158235	0.9793568	0.02986	3.521891
**159**	GO:0019221~cytokine-mediated signaling pathway	6	5.128205	0.002151	IL6, CCL2, IL10RB, RELA, IL1B, IL1A	6.57349775	0.9810903	0.030292	3.600048

**160**	GO:2001237~negative regulation of extrinsic apoptotic signaling pathway	4	3.418803	0.002272	IL4, AR, RELA, GSTP1	15.1075124	0.9848881	0.031734	3.799538
**161**	GO:0001503~ossification	5	4.273504	0.002283	EGFR, BCL2, MMP9, IGF2, SPP1	8.97008547	0.9851954	0.031647	3.817798
**162**	GO:0007204~positive regulation of cytosolic calcium ion concentration	6	5.128205	0.002374	OPRM1, PTGER3, ADRA1B, ESR1, GJA1, IL2	6.42632989	0.9874739	0.032637	3.966203
**163**	GO:0032570~response to progesterone	4	3.418803	0.002449	FOS, CCL2, CLDN4, RELA	14.7201403	0.9891038	0.033409	4.089825
**164**	GO:0000302~response to reactive oxygen species	4	3.418803	0.002449	GSR, CAT, SOD1, GSTP1	14.7201403	0.9891038	0.033409	4.089825
**165**	GO:0030890~positive regulation of B cell proliferation	4	3.418803	0.002449	IL4, CDKN1A, BCL2, IL2	14.7201403	0.9891038	0.033409	4.089825
**166**	GO:0032495~response to muramyl dipeptide	3	2.564103	0.002498	MAPK14, RELA, NFKBIA	39.1421911	0.9900494	0.033819	4.170239
**167**	GO:0030278~regulation of ossification	3	2.564103	0.002498	MAPK1, MAPK14, MAPK3	39.1421911	0.9900494	0.033819	4.170239
**168**	GO:0043124~negative regulation of I-kappaB kinase/NF-kappaB signaling	4	3.418803	0.002635	CASP8, ESR1, STAT1, GSTP1	14.3521368	0.9922637	0.035373	4.392857
**169**	GO:0051262~protein tetramerization	4	3.418803	0.002635	ACHE, HMGCR, TP63, CAT	14.3521368	0.9922637	0.035373	4.392857
**170**	GO:0030307~positive regulation of cell growth	5	4.273504	0.002727	AKT1, EGFR, BCL2, ERBB2, IL2	8.54293854	0.9934834	0.036334	4.544297
**171**	GO:0001822~kidney development	5	4.273504	0.00297	ODC1, BAX, VEGFA, HAS2, CA2	8.34426555	0.9958398	0.039226	4.939315
**172**	GO:0042738~exogenous drug catabolic process	3	2.564103	0.002984	CYP3A4, NR1I2, CYP1A2	35.8803419	0.9959481	0.039131	4.962477
**173**	GO:0071498~cellular response to fluid shear stress	3	2.564103	0.002984	PTGS2, HAS2, CA2	35.8803419	0.9959481	0.039131	4.962477
**174**	GO:0008202~steroid metabolic process	4	3.418803	0.003242	AKR1C3, CYP3A4, NR1I2, CYP1A1	13.3508249	0.9974818	0.042138	5.379242
**175**	GO:0048538~thymus development	4	3.418803	0.003242	MAPK1, BCL2, MAPK3, SOD1	13.3508249	0.9974818	0.042138	5.379242
**176**	GO:0007565~female pregnancy	5	4.273504	0.003362	IL4, FOS, THBD, CLDN4, BCL2	8.06299818	0.9979821	0.043358	5.572693
**177**	GO:0051926~negative regulation of calcium ion transport	3	2.564103	0.003511	ICAM1, PTGS2, NOS3	33.1203156	0.9984701	0.044934	5.813904
**178**	GO:0048546~digestive tract morphogenesis	3	2.564103	0.003511	EGFR, HIF1A, BCL2	33.1203156	0.9984701	0.044934	5.813904
**179**	GO:0097296~activation of cysteine-type endopeptidase activity involved in apoptotic signaling pathway	3	2.564103	0.003511	BAX, CASP8, FASLG	33.1203156	0.9984701	0.044934	5.813904
**180**	GO:0045780~positive regulation of bone resorption	3	2.564103	0.003511	EGFR, CA2, SPP1	33.1203156	0.9984701	0.044934	5.813904
**181**	GO:0071318~cellular response to ATP	3	2.564103	0.003511	CCL2, PTGS2, SOD1	33.1203156	0.9984701	0.044934	5.813904
**182**	GO:0071363~cellular response to growth factor stimulus	4	3.418803	0.00369	ERBB2, MDM2, CAT, HTR3A	12.7574549	0.9989016	0.046852	6.101833
**183**	GO:0019395~fatty acid oxidation	3	2.564103	0.004078	MAPK14, PPARG, POR	30.7545788	0.9994638	0.051302	6.721994
**184**	GO:0032461~positive regulation of protein oligomerization	3	2.564103	0.004078	BAX, MMP3, MMP1	30.7545788	0.9994638	0.051302	6.721994
**185**	GO:2000353~positive regulation of endothelial cell apoptotic process	3	2.564103	0.004078	AKR1C3, CD40LG, FASLG	30.7545788	0.9994638	0.051302	6.721994
**186**	GO:0035729~cellular response to hepatocyte growth factor stimulus	3	2.564103	0.004078	IL6, RELA, GSK3B	30.7545788	0.9994638	0.051302	6.721994
**187**	GO:0071230~cellular response to amino acid stimulus	4	3.418803	0.004175	EGFR, COL1A1, BCL2L1, MMP2	12.2145845	0.999552	0.052138	6.876726
**188**	GO:0031100~organ regeneration	4	3.418803	0.004175	CDKN1A, CCL2, PPARG, GSTP1	12.2145845	0.999552	0.052138	6.876726
**189**	GO:1904707~positive regulation of vascular smooth muscle cell proliferation	3	2.564103	0.004684	MMP9, MDM2, MMP2	28.7042735	0.9998254	0.057932	7.684169
**190**	GO:0046688~response to copper ion	3	2.564103	0.004684	ICAM1, SOD1, IL1A	28.7042735	0.9998254	0.057932	7.684169
**191**	GO:0042752~regulation of circadian rhythm	4	3.418803	0.004697	PPARA, PPARG, MAPK8, TOP2A	11.71603	0.9998294	0.057696	7.703934
**192**	GO:0042593~glucose homeostasis	5	4.273504	0.005277	AKT1, IL6, HIF1A, PPARG, ADRA1B	7.10501819	0.9999418	0.064187	8.616569
**193**	GO:0008209~androgen metabolic process	3	2.564103	0.005329	CYP3A4, ESR1, CYP19A1	26.9102564	0.9999472	0.064376	8.6978
**194**	GO:0019233~sensory perception of pain	4	3.418803	0.00555	OPRM1, MAPK1, PTGS2, MAPK3	11.0401052	0.9999649	0.066525	9.04235
**195**	GO:0002576~platelet degranulation	5	4.273504	0.005655	ALB, VEGFA, IGF2, SOD1, EGF	6.96705668	0.9999711	0.067306	9.205525
**196**	GO:0045727~positive regulation of translation	4	3.418803	0.005854	MAPK1, IL6, ERBB2, MAPK3	10.8318013	0.99998	0.069151	9.5143
**197**	GO:0070542~response to fatty acid	3	2.564103	0.006013	PTGS2, PON1, CAT	25.3273002	0.9999851	0.070515	9.760227
**198**	GO:2000811~negative regulation of anoikis	3	2.564103	0.006013	MCL1, BCL2, BCL2L1	25.3273002	0.9999851	0.070515	9.760227
**199**	GO:0030224~monocyte differentiation	3	2.564103	0.006013	JUN, VEGFA, PPARG	25.3273002	0.9999851	0.070515	9.760227
**200**	GO:0048146~positive regulation of fibroblast proliferation	4	3.418803	0.006167	EGFR, CDKN1A, JUN, ESR1	10.6312124	0.9999888	0.071812	9.999029
**201**	GO:0030225~macrophage differentiation	3	2.564103	0.006734	MMP9, CASP8, VEGFA	23.9202279	0.9999961	0.077678	10.86876
**202**	GO:0033280~response to vitamin D	3	2.564103	0.006734	PTGS2, SPP1, CXCL10	23.9202279	0.9999961	0.077678	10.86876
**203**	GO:0002690~positive regulation of leukocyte chemotaxis	3	2.564103	0.006734	IL6, CXCL11, CXCL10	23.9202279	0.9999961	0.077678	10.86876
**204**	GO:0051412~response to corticosterone	3	2.564103	0.006734	FOS, CDKN1A, CCND1	23.9202279	0.9999961	0.077678	10.86876
**205**	GO:0010039~response to iron ion	3	2.564103	0.006734	CCND1, BCL2, MDM2	23.9202279	0.9999961	0.077678	10.86876
**206**	GO:0006749~glutathione metabolic process	4	3.418803	0.006824	GSTM1, GSR, SOD1, GSTP1	10.2515263	0.9999967	0.078192	11.00643

**207**	GO:0070301~cellular response to hydrogen peroxide	4	3.418803	0.007167	IL6, RELA, MDM2, PRKCD	10.0716749	0.9999983	0.081471	11.52888
**208**	GO:0046827~positive regulation of protein export from nucleus	3	2.564103	0.007492	GSK3B, MDM2, IL1B	22.6612686	0.999999	0.084498	12.02068
**209**	GO:0046716~muscle cell cellular homeostasis	3	2.564103	0.007492	IL6, HIF1A, SOD1	22.6612686	0.999999	0.084498	12.02068
**210**	GO:0071880~adenylate cyclase-activating adrenergic receptor signaling pathway	3	2.564103	0.007492	ADRB2, ADRB1, ADRA1B	22.6612686	0.999999	0.084498	12.02068
**211**	GO:0051259~protein oligomerization	4	3.418803	0.007521	AR, BAX, SLC6A4, GJA1	9.89802535	0.9999991	0.084293	12.06365
**212**	GO:0045892~negative regulation of transcription, DNA-templated	10	8.547009	0.007741	IL4, PPARD, NR1I2, RELA, JUN, PPARG, IRF1, TP63, MDM2, AHR	2.87617971	0.9999994	0.086134	12.39458
**213**	GO:0050776~regulation of immune response	6	5.128205	0.007881	IL4, VCAM1, ICAM1, CD40LG, IRF1, COL1A1	4.83779891	0.9999995	0.087107	12.60515
**214**	GO:0043406~positive regulation of MAP kinase activity	4	3.418803	0.007884	EGFR, ERBB2, VEGFA, EGF	9.7302622	0.9999995	0.086628	12.61061
**215**	GO:0050679~positive regulation of epithelial cell proliferation	4	3.418803	0.008258	EGFR, IL6, ERBB2, VEGFA	9.56809117	0.9999998	0.090027	13.16959
**216**	GO:0048545~response to steroid hormone	3	2.564103	0.008288	MDM2, CA2, SPP1	21.5282051	0.9999998	0.089804	13.21329
**217**	GO:0071375~cellular response to peptide hormone stimulus	3	2.564103	0.008288	RELA, MDM2, POR	21.5282051	0.9999998	0.089804	13.21329
**218**	GO:0046697~decidualization	3	2.564103	0.008288	PPARD, PTGS2, SPP1	21.5282051	0.9999998	0.089804	13.21329
**219**	GO:0006950~response to stress	4	3.418803	0.008642	EGFR, MAPK1, HSP90AA1, MAPK8	9.41123721	0.9999999	0.092936	13.74044
**220**	GO:0007507~heart development	6	5.128205	0.008824	PPARA, PPARD, CASP7, ERBB2, PPARG, GJA1	4.70561861	0.9999999	0.094258	14.00951
**221**	GO:0016525~negative regulation of angiogenesis	4	3.418803	0.009037	CCL2, FASLG, STAT1, CXCL10	9.25944307	0.9999999	0.095872	14.32297
**222**	GO:0044267~cellular protein metabolic process	5	4.273504	0.009073	BACE1, IGF2, IGFBP3, MMP2, MMP1	6.08141388	0.9999999	0.09569	14.37608
**223**	GO:0001701~in utero embryonic development	6	5.128205	0.009633	AR, RXRA, VEGFA, GJA1, NOS3, BCL2L1	4.60496366	1	0.100745	15.19675
**224**	GO:0030308~negative regulation of cell growth	5	4.273504	0.009888	CDKN1A, PPARD, BCL2, PPARG, GJA1	5.93063502	1	0.1027	15.56729
**225**	GO:0051000~positive regulation of nitric-oxide synthase activity	3	2.564103	0.009986	AKT1, HIF1A, ESR1	19.5710956	1	0.103092	15.70964
**226**	GO:0051384~response to glucocorticoid	4	3.418803	0.010282	CASP3, IL6, PTGS2, BCL2	8.83208416	1	0.105415	16.13877
**227**	GO:0007200~phospholipase C-activating G-protein coupled receptor signaling pathway	4	3.418803	0.010719	OPRM1, PTGER3, ADRA1B, ESR1	8.6982647	1	0.109055	16.76608
**228**	GO:0000122~negative regulation of transcription from RNA polymerase II promoter	12	10.25641	0.011082	PPARA, PPARD, CCND1, RELA, RXRA, VEGFA, PPARG, ESR1, TP63, MDM2, FASLG, STAT1	2.39202279	1	0.111938	17.28447
**229**	GO:0006006~glucose metabolic process	4	3.418803	0.011166	AKT1, PPARD, MAPK14, IGF2	8.56843985	1	0.112131	17.40405
**230**	GO:0032868~response to insulin	4	3.418803	0.011166	PPARA, IL6, RELA, CAT	8.56843985	1	0.112131	17.40405
**231**	GO:0070371~ERK1 and ERK2 cascade	3	2.564103	0.011824	MAPK1, MAPK3, EGF	17.9401709	1	0.117742	18.33616
**232**	GO:0045742~positive regulation of epidermal growth factor receptor signaling pathway	3	2.564103	0.011824	AKT1, MMP9, FASLG	17.9401709	1	0.117742	18.33616
**233**	GO:0007166~cell surface receptor signaling pathway	7	5.982906	0.011916	EGFR, ADRB2, CCL2, MAPK14, ERBB2, CASP8, CXCL10	3.66660428	1	0.117966	18.46441
**234**	GO:0035690~cellular response to drug	4	3.418803	0.012091	EGFR, CCL2, IL1B, NOS2	8.32007928	1	0.118974	18.71098
**235**	GO:0071300~cellular response to retinoic acid	4	3.418803	0.01257	CCL2, SLC6A4, PPARG, COL1A1	8.201221	1	0.122762	19.37945
**236**	GO:0007569~cell aging	3	2.564103	0.012795	ICAM1, BCL2, SOD1	17.2225641	1	0.124176	19.69154
**237**	GO:0042327~positive regulation of phosphorylation	3	2.564103	0.012795	EGFR, AR, EGF	17.2225641	1	0.124176	19.69154
**238**	GO:0070098~chemokine-mediated signaling pathway	4	3.418803	0.01306	CCL2, CXCL2, CXCL11, CXCL10	8.08571085	1	0.125935	20.05758
**239**	GO:0032227~negative regulation of synaptic transmission, dopaminergic	2	1.709402	0.013769	PTGS2, SLC6A4	143.521368	1	0.13166	21.02973
**240**	GO:0035408~histone H3-T6 phosphorylation	2	1.709402	0.013769	PRKCA, PRKCB	143.521368	1	0.13166	21.02973
**241**	GO:0046666~retinal cell programmed cell death	2	1.709402	0.013769	BAX, FASLG	143.521368	1	0.13166	21.02973
**242**	GO:0001912~positive regulation of leukocyte mediated cytotoxicity	2	1.709402	0.013769	CCL2, NOS2	143.521368	1	0.13166	21.02973
**243**	GO:0014806~smooth muscle hyperplasia	2	1.709402	0.013769	HMOX1, NOS3	143.521368	1	0.13166	21.02973
**244**	GO:0006808~regulation of nitrogen utilization	2	1.709402	0.013769	BAX, BCL2	143.521368	1	0.13166	21.02973
**245**	GO:0071306~cellular response to vitamin E	2	1.709402	0.013769	PPARG, COL1A1	143.521368	1	0.13166	21.02973
**246**	GO:0007202~activation of phospholipase C activity	3	2.564103	0.013799	EGFR, PRKCD, SELE	16.5601578	1	0.13126	21.07149
**247**	GO:0002053~positive regulation of mesenchymal cell proliferation	3	2.564103	0.013799	VEGFA, TP63, STAT1	16.5601578	1	0.13126	21.07149
**248**	GO:0007155~cell adhesion	9	7.692308	0.014304	PRKCA, VCAM1, ICAM1, ACHE, CCL2, COL1A1, SELE, SPP1, IL2	2.81414446	1	0.135059	21.75603
**249**	GO:0017144~drug metabolic process	3	2.564103	0.014837	CYP3A4, CYP1A1, CYP1A2	15.9468186	1	0.139054	22.47343
**250**	GO:0010800~positive regulation of peptidyl-threonine phosphorylation	3	2.564103	0.014837	MAPK1, GSK3B, EGF	15.9468186	1	0.139054	22.47343

251	GO:0051281~positive regulation of release of sequestered calcium ion into cytosol	3	2.564103	0.014837	BAX, CXCL11, CXCL10	15.9468186	1	0.139054	22.47343
252	GO:0042104~positive regulation of activated T cell proliferation	3	2.564103	0.014837	IL4, IGF2, IL2	15.9468186	1	0.139054	22.47343
253	GO:0010629~negative regulation of gene expression	5	4.273504	0.015035	AKT1, CDKN1A, ESR1, GJA1, NOS2	5.23800611	1	0.140084	22.73891
254	GO:1900034~regulation of cellular response to heat	4	3.418803	0.015127	MAPK1, HSP90AA1, GSK3B, MAPK3	7.65447293	1	0.140179	22.8612
255	GO:0030324~lung development	4	3.418803	0.015671	EGFR, VEGFA, NOS3, CYP1A2	7.55375619	1	0.144156	23.58346
256	GO:2000145~regulation of cell motility	3	2.564103	0.015907	EGFR, ERBB2, EGF	15.3772894	1	0.145458	23.89481
257	GO:0000060~protein import into nucleus, translocation	3	2.564103	0.015907	AKR1C3, AKT1, NFKBIA	15.3772894	1	0.145458	23.89481
258	GO:0009314~response to radiation	3	2.564103	0.015907	OPRM1, JUN, BCL2	15.3772894	1	0.145458	23.89481
259	GO:0043392~negative regulation of DNA binding	3	2.564103	0.015907	JUN, HMOX1, NFKBIA	15.3772894	1	0.145458	23.89481
260	GO:0008360~regulation of cell shape	5	4.273504	0.016157	ICAM1, IL6, CCL2, VEGFA, RASA1	5.12576313	1	0.146867	24.22395
261	GO:0045454~cell redox homeostasis	4	3.418803	0.016226	GSR, IL6, NOS3, NOS2	7.45565546	1	0.14673	24.31365
262	GO:0043547~positive regulation of GTPase activity	10	8.547009	0.016413	EGFR, ICAM1, CCL2, ADRB1, JUN, GSK3B, ERBB2, EGF, RASA1, IL2	2.5402012	1	0.147585	24.5582
263	GO:0071549~cellular response to dexamethasone stimulus	3	2.564103	0.017009	EGFR, IL6, CCL2	14.847038	1	0.151827	25.33315
264	GO:0043542~endothelial cell migration	3	2.564103	0.017009	NOS3, STAT1, DPP4	14.847038	1	0.151827	25.33315
265	GO:0042113~B cell activation	3	2.564103	0.017009	IL4, CASP8, PRKCB	14.847038	1	0.151827	25.33315
266	GO:0050728~negative regulation of inflammatory response	4	3.418803	0.017368	PPARA, PPARD, PRKCD, IL2	7.26690468	1	0.154062	25.79665
267	GO:0007049~cell cycle	6	5.128205	0.017376	MAPK1, CCND1, MAPK3, TP63, PRKCD, AHR	3.96833274	1	0.153401	25.80745
268	GO:0016032~viral process	7	5.982906	0.017621	VCAM1, MAPK1, BAX, MAPK3, NFKBIA, MDM2, MMP1	3.36003202	1	0.154668	26.12113
269	GO:0007281~germ cell development	3	2.564103	0.018142	AKT1, BAX, BCL2L1	14.3521368	1	0.158155	26.786
270	GO:2000379~positive regulation of reactive oxygen species metabolic process	3	2.564103	0.018142	AKR1C3, CDKN1A, MAPK14	14.3521368	1	0.158155	26.786
271	GO:0045776~negative regulation of blood pressure	3	2.564103	0.018142	PPARA, NOS3, NOS2	14.3521368	1	0.158155	26.786
272	GO:0043085~positive regulation of catalytic activity	4	3.418803	0.018555	BCL2, IGF2, SOD1, IGFBP3	7.08747494	1	0.160723	27.30767
273	GO:0050731~positive regulation of peptidyl-tyrosine phosphorylation	4	3.418803	0.019164	ICAM1, IL6, VEGFA, IGF2	7.00104232	1	0.164825	28.07288
274	GO:0071392~cellular response to estradiol stimulus	3	2.564103	0.019307	EGFR, IL6, ESR1	13.8891646	1	0.165195	28.25098
275	GO:0045765~regulation of angiogenesis	3	2.564103	0.019307	IL6, HMOX1, ERBB2	13.8891646	1	0.165195	28.25098
276	GO:0051897~positive regulation of protein kinase B signaling	4	3.418803	0.020417	AKR1C3, EGFR, IL6, IGF2	6.83435083	1	0.173113	29.62108
277	GO:0050873~brown fat cell differentiation	3	2.564103	0.020502	ADRB2, ADRB1, PTGS2	13.4551282	1	0.172994	29.72578
278	GO:0042177~negative regulation of protein catabolic process	3	2.564103	0.020502	EGFR, RELA, NOS2	13.4551282	1	0.172994	29.72578
279	GO:0001974~blood vessel remodeling	3	2.564103	0.020502	BAX, MDM2, NOS3	13.4551282	1	0.172994	29.72578
280	GO:0042100~B cell proliferation	3	2.564103	0.020502	CD40LG, BCL2, PRKCD	13.4551282	1	0.172994	29.72578
281	GO:0010829~negative regulation of glucose transport	2	1.709402	0.020583	IL1B, PRKCB	95.6809117	1	0.172834	29.82364
282	GO:0001660~fever generation	2	1.709402	0.020583	IL1B, IL1A	95.6809117	1	0.172834	29.82364
283	GO:0061308~cardiac neural crest cell development involved in heart development	2	1.709402	0.020583	MAPK1, MAPK3	95.6809117	1	0.172834	29.82364
284	GO:0002693~positive regulation of cellular extravasation	2	1.709402	0.020583	ICAM1, CCL2	95.6809117	1	0.172834	29.82364
285	GO:0031649~heat generation	2	1.709402	0.020583	ADRB2, ADRB1	95.6809117	1	0.172834	29.82364
286	GO:0000189~MAPK import into nucleus	2	1.709402	0.020583	MAPK1, MAPK3	95.6809117	1	0.172834	29.82364
287	GO:0010871~negative regulation of receptor biosynthetic process	2	1.709402	0.020583	PPARA, PPARG	95.6809117	1	0.172834	29.82364
288	GO:0033590~response to cobalamin	2	1.709402	0.020583	EGFR, RELA	95.6809117	1	0.172834	29.82364
289	GO:0002025~vasodilation by norepinephrine-epinephrine involved in regulation of systemic arterial blood pressure	2	1.709402	0.020583	ADRB2, ADRB1	95.6809117	1	0.172834	29.82364
290	GO:0018108~peptidyl-tyrosine phosphorylation	5	4.273504	0.02162	EGFR, HSP90AA1, ERBB2, EGF, PRKCD	4.69024077	1	0.17999	31.07896
291	GO:0043491~protein kinase B signaling	3	2.564103	0.021728	AKT1, CCL2, IL1B	13.047397	1	0.180008	31.20815
292	GO:0070555~response to interleukin-1	3	2.564103	0.021728	PRKCA, RELA, SELE	13.047397	1	0.180008	31.20815
293	GO:2001244~positive regulation of intrinsic apoptotic signaling pathway	3	2.564103	0.021728	BAX, BCL2, BCL2L1	13.047397	1	0.180008	31.20815
294	GO:0070059~intrinsic apoptotic signaling pathway in response to endoplasmic reticulum stress	3	2.564103	0.021728	BAX, BCL2, GSK3B	13.047397	1	0.180008	31.20815

**295**	GO:0043330~response to exogenous dsRNA	3	2.564103	0.022983	MAPK1, MAPK3, NFKBIA	12.6636501	1	0.188634	32.6959
**296**	GO:0006629~lipid metabolic process	5	4.273504	0.023502	CYP3A4, PPARA, PPARD, PTGS1, PPARG	4.57074419	1	0.191664	33.30251
**297**	GO:0001890~placenta development	3	2.564103	0.024268	MAPK14, PPARG, SOD1	12.3018315	1	0.19646	34.18691
**298**	GO:0006366~transcription from RNA polymerase II promoter	9	7.692308	0.025877	FOS, HIF1A, RELA, JUN, IRF1, ESR1, TP63, NR3C1, AHR	2.51791873	1	0.207291	36.01072
**299**	GO:0008584~male gonad development	4	3.418803	0.027341	AKR1C3, BCL2, ESR1, BCL2L1	6.10729223	1	0.216871	37.6297
**300**	GO:0060574~intestinal epithelial cell maturation	2	1.709402	0.02735	CDKN1A, HIF1A	71.7606838	1	0.216018	37.63872
**301**	GO:0002677~negative regulation of chronic inflammatory response	2	1.709402	0.02735	IL4, CYP19A1	71.7606838	1	0.216018	37.63872
**302**	GO:1900015~regulation of cytokine production involved in inflammatory response	2	1.709402	0.02735	MAPK14, NOS2	71.7606838	1	0.216018	37.63872
**303**	GO:0070849~response to epidermal growth factor	2	1.709402	0.02735	MAPK1, MAPK3	71.7606838	1	0.216018	37.63872
**304**	GO:0010574~regulation of vascular endothelial growth factor	2	1.709402	0.02735	IL6, CCL2	71.7606838	1	0.216018	37.63872
**305**	GO:1900127~positive regulation of hyaluronan biosynthetic process	2	1.709402	0.02735	HAS2, EGF	71.7606838	1	0.216018	37.63872
**306**	GO:0002248~connective tissue replacement involved in inflammatory response wound healing	2	1.709402	0.02735	HIF1A, IL1A	71.7606838	1	0.216018	37.63872
**307**	GO:0090400~stress-induced premature senescence	2	1.709402	0.02735	CDKN1A, MAPK14	71.7606838	1	0.216018	37.63872
**308**	GO:0032079~positive regulation of endodeoxyribonuclease activity	2	1.709402	0.02735	AKT1, PRKCD	71.7606838	1	0.216018	37.63872
**309**	GO:0060571~morphogenesis of an epithelial fold	2	1.709402	0.02735	EGFR, AR	71.7606838	1	0.216018	37.63872
**310**	GO:0045986~negative regulation of smooth muscle contraction	2	1.709402	0.02735	ADRB2, PTGS2	71.7606838	1	0.216018	37.63872
**311**	GO:0006778~porphyrin-containing compound metabolic process	2	1.709402	0.02735	CYP1A1, CYP1A2	71.7606838	1	0.216018	37.63872
**312**	GO:0061419~positive regulation of transcription from RNA polymerase II promoter in response to hypoxia	2	1.709402	0.02735	HIF1A, VEGFA	71.7606838	1	0.216018	37.63872
**313**	GO:0090170~regulation of Golgi inheritance	2	1.709402	0.02735	MAPK1, MAPK3	71.7606838	1	0.216018	37.63872
**314**	GO:0070988~demethylation	2	1.709402	0.02735	CYP1A1, POR	71.7606838	1	0.216018	37.63872
**315**	GO:0000320~re-entry into mitotic cell cycle	2	1.709402	0.02735	CCND1, GSK3B	71.7606838	1	0.216018	37.63872
**316**	GO:0008625~extrinsic apoptotic signaling pathway via death domain receptors	3	2.564103	0.028292	BAX, BCL2, FASLG	11.3306343	1	0.22173	38.65956
**317**	GO:0001568~blood vessel development	3	2.564103	0.028292	MDM2, COL1A1, AHR	11.3306343	1	0.22173	38.65956
**318**	GO:1901215~negative regulation of neuron death	3	2.564103	0.031113	AKT1, PPARA, IL6	10.7641026	1	0.240255	41.62235
**319**	GO:0001895~retina homeostasis	3	2.564103	0.031113	ALB, HSPB1, SOD1	10.7641026	1	0.240255	41.62235
**320**	GO:0007190~activation of adenylate cyclase activity	3	2.564103	0.031113	PRKCA, ADRB2, ADRB1	10.7641026	1	0.240255	41.62235
**321**	GO:0006469~negative regulation of protein kinase activity	4	3.418803	0.031217	AKT1, IL6, HSPB1, GSTP1	5.79884313	1	0.239979	41.72879
**322**	GO:0010468~regulation of gene expression	4	3.418803	0.032024	HIF1A, BCL2, CYP1A2, AHR	5.7408547	1	0.244452	42.55078
**323**	GO:0030163~protein catabolic process	3	2.564103	0.032563	AKT1, BACE1, CTSD	10.5015635	1	0.247068	43.09288
**324**	GO:0007595~lactation	3	2.564103	0.03404	CCND1, HIF1A, VEGFA	10.2515263	1	0.255841	44.55405
**325**	GO:0045740~positive regulation of DNA replication	3	2.564103	0.03404	EGFR, IL6, JUN	10.2515263	1	0.255841	44.55405
**326**	GO:0097191~extrinsic apoptotic signaling pathway	3	2.564103	0.03404	BAX, CASP8, FASLG	10.2515263	1	0.255841	44.55405
**327**	GO:0099565~chemical synaptic transmission, postsynaptic	2	1.709402	0.03407	AKT1, GSK3B	57.408547	1	0.255026	44.58388
**328**	GO:0006706~steroid catabolic process	2	1.709402	0.03407	CYP3A4, CYP1A2	57.408547	1	0.255026	44.58388
**329**	GO:0033591~response to L-ascorbic acid	2	1.709402	0.03407	CAT, GSTP1	57.408547	1	0.255026	44.58388
**330**	GO:0022614~membrane to membrane docking	2	1.709402	0.03407	VCAM1, ICAM1	57.408547	1	0.255026	44.58388
**331**	GO:0010891~negative regulation of sequestering of triglyceride	2	1.709402	0.03407	PPARA, PPARG	57.408547	1	0.255026	44.58388
**332**	GO:0070431~nucleotide-binding oligomerization domain containing 2 signaling pathway	2	1.709402	0.03407	RELA, NFKBIA	57.408547	1	0.255026	44.58388
**333**	GO:0072584~caveolin-mediated endocytosis	2	1.709402	0.03407	MAPK1, MAPK3	57.408547	1	0.255026	44.58388
**334**	GO:0038033~positive regulation of endothelial cell chemotaxis by VEGF-activated vascular endothelial growth factor receptor signaling pathway	2	1.709402	0.03407	VEGFA, HSPB1	57.408547	1	0.255026	44.58388
**335**	GO:0043488~regulation of mRNA stability	4	3.418803	0.034513	PRKCA, AKT1, HSPB1, PRKCD	5.57364534	1	0.256908	45.01516
**336**	GO:0045785~positive regulation of cell adhesion	3	2.564103	0.035542	PRKCA, ERBB2, VEGFA	10.0131187	1	0.262545	46.00438

**337**	GO:0014823~response to activity	3	2.564103	0.035542	PPARD, CCL2, CAT	10.0131187	1	0.262545	46.00438
**338**	GO:0006879~cellular iron ion homeostasis	3	2.564103	0.03707	HIF1A, HMOX1, SOD1	9.78554779	1	0.271266	47.44246
**339**	GO:0034644~cellular response to UV	3	2.564103	0.03707	PTGS2, BAX, TP63	9.78554779	1	0.271266	47.44246
**340**	GO:0043434~response to peptide hormone	3	2.564103	0.03707	GJA1, COL1A1, STAT1	9.78554779	1	0.271266	47.44246
**341**	GO:0048015~phosphatidylinositol-mediated signaling	4	3.418803	0.0371	AKT1, EGFR, ERBB2, EGF	5.41590066	1	0.270412	47.47049
**342**	GO:0032755~positive regulation of interleukin-6 production	3	2.564103	0.038622	IL6, IL1B, IL1A	9.56809117	1	0.27891	48.86698
**343**	GO:0003151~outflow tract morphogenesis	3	2.564103	0.040199	HIF1A, JUN, VEGFA	9.36008919	1	0.287584	50.2767
**344**	GO:0009615~response to virus	4	3.418803	0.040699	ODC1, CYP1A1, HSPB1, IFNGR1	5.21895882	1	0.289556	50.71587
**345**	GO:0043619~regulation of transcription from RNA polymerase II promoter in response to oxidative stress	2	1.709402	0.040745	HIF1A, HMOX1	47.8404558	1	0.288754	50.75591
**346**	GO:0030816~positive regulation of cAMP metabolic process	2	1.709402	0.040745	CXCL11, CXCL10	47.8404558	1	0.288754	50.75591
**347**	GO:0042737~drug catabolic process	2	1.709402	0.040745	CYP3A4, CYP1A2	47.8404558	1	0.288754	50.75591
**348**	GO:0032025~response to cobalt ion	2	1.709402	0.040745	CASP3, CASP8	47.8404558	1	0.288754	50.75591
**349**	GO:0046321~positive regulation of fatty acid oxidation	2	1.709402	0.040745	PPARA, PPARG	47.8404558	1	0.288754	50.75591
**350**	GO:0016098~monoterpenoid metabolic process	2	1.709402	0.040745	CYP3A4, CYP1A2	47.8404558	1	0.288754	50.75591
**351**	GO:0010742~macrophage derived foam cell differentiation	2	1.709402	0.040745	PPARG, STAT1	47.8404558	1	0.288754	50.75591
**352**	GO:0010887~negative regulation of cholesterol storage	2	1.709402	0.040745	PPARA, PPARG	47.8404558	1	0.288754	50.75591
**353**	GO:0046483~heterocycle metabolic process	2	1.709402	0.040745	CYP3A4, CYP1A2	47.8404558	1	0.288754	50.75591
**354**	GO:0045908~negative regulation of vasodilation	2	1.709402	0.040745	HMGCR, CRP	47.8404558	1	0.288754	50.75591
**355**	GO:0060440~trachea formation	2	1.709402	0.040745	MAPK1, MAPK3	47.8404558	1	0.288754	50.75591
**356**	GO:0002933~lipid hydroxylation	2	1.709402	0.040745	CYP3A4, CYP1A1	47.8404558	1	0.288754	50.75591
**357**	GO:0090336~positive regulation of brown fat cell differentiation	2	1.709402	0.040745	PTGS2, MAPK14	47.8404558	1	0.288754	50.75591
**358**	GO:0045084~positive regulation of interleukin-12 biosynthetic process	2	1.709402	0.040745	RELA, IRF1	47.8404558	1	0.288754	50.75591
**359**	GO:0060137~maternal process involved in parturition	2	1.709402	0.040745	CCL2, CYP1A1	47.8404558	1	0.288754	50.75591
**360**	GO:0001957~intramembranous ossification	2	1.709402	0.040745	COL1A1, MMP2	47.8404558	1	0.288754	50.75591
**361**	GO:0045600~positive regulation of fat cell differentiation	3	2.564103	0.0418	AKT1, PPARD, PPARG	9.16093835	1	0.294047	51.67045
**362**	GO:0014070~response to organic cyclic compound	3	2.564103	0.045073	IL4, ICAM1, CYP1A1	8.7870225	1	0.312333	54.40582
**363**	GO:0007157~heterophilic cell-cell adhesion via plasma membrane cell adhesion molecules	3	2.564103	0.046744	VCAM1, ICAM1, SELE	8.61128205	1	0.320885	55.7455
**364**	GO:0060687~regulation of branching involved in prostate gland morphogenesis	2	1.709402	0.047374	RXRA, ESR1	41.006105	1	0.323345	56.24083
**365**	GO:0090050~positive regulation of cell migration involved in sprouting angiogenesis	2	1.709402	0.047374	PTGS2, VEGFA	41.006105	1	0.323345	56.24083
**366**	GO:0043496~regulation of protein homodimerization activity	2	1.709402	0.047374	BAX, BCL2	41.006105	1	0.323345	56.24083
**367**	GO:0035357~peroxisome proliferator activated receptor signaling pathway	2	1.709402	0.047374	RXRA, PPARG	41.006105	1	0.323345	56.24083
**368**	GO:0030213~hyaluronan biosynthetic process	2	1.709402	0.047374	IL1B, HAS2	41.006105	1	0.323345	56.24083
**369**	GO:0051549~positive regulation of keratinocyte migration	2	1.709402	0.047374	MMP9, HAS2	41.006105	1	0.323345	56.24083
**370**	GO:0071639~positive regulation of monocyte chemotactic protein-1 production	2	1.709402	0.047374	IL1B, IL1A	41.006105	1	0.323345	56.24083
**371**	GO:0032287~peripheral nervous system myelin maintenance	2	1.709402	0.047374	AKT1, SOD1	41.006105	1	0.323345	56.24083
**372**	GO:0033209~tumor necrosis factor-mediated signaling pathway	4	3.418803	0.048406	CD40LG, TNFSF15, FASLG, STAT1	4.8651311	1	0.328058	57.04137
**373**	GO:0071277~cellular response to calcium ion	3	2.564103	0.048437	AKR1C3, FOS, JUN	8.44243338	1	0.327077	57.06534
**374**	GO:0032880~regulation of protein localization	3	2.564103	0.050153	AKT1, BCL2, MAPK8	8.2800789	1	0.335521	58.36458
**375**	GO:0050853~B cell receptor signaling pathway	3	2.564103	0.053649	MAPK1, BCL2, PRKCB	7.97340931	1	0.353487	60.89844
**376**	GO:0048511~rhythmic process	3	2.564103	0.053649	PPARG, MAPK8, TOP2A	7.97340931	1	0.353487	60.89844
**377**	GO:2000641~regulation of early endosome to late endosome transport	2	1.709402	0.053957	MAPK1, MAPK3	35.8803419	1	0.353943	61.1151
**378**	GO:0060736~prostate gland growth	2	1.709402	0.053957	AR, CYP19A1	35.8803419	1	0.353943	61.1151
**379**	GO:0006527~arginine catabolic process	2	1.709402	0.053957	NOS3, NOS2	35.8803419	1	0.353943	61.1151

**380**	GO:0009308~amine metabolic process	2	1.709402	0.053957	VCAM1, CYP1A1	35.8803419	1	0.353943	61.1151
**381**	GO:0009812~flavonoid metabolic process	2	1.709402	0.053957	CYP1A1, POR	35.8803419	1	0.353943	61.1151
**382**	GO:0033129~positive regulation of histone phosphorylation	2	1.709402	0.053957	MAPK3, IL1B	35.8803419	1	0.353943	61.1151
**383**	GO:0002438~acute inflammatory response to antigenic stimulus	2	1.709402	0.053957	OPRM1, ICAM1	35.8803419	1	0.353943	61.1151
**384**	GO:0010818~T cell chemotaxis	2	1.709402	0.053957	CXCL11, CXCL10	35.8803419	1	0.353943	61.1151
**385**	GO:1904706~negative regulation of vascular smooth muscle cell proliferation	2	1.709402	0.053957	HMOX1, GSTP1	35.8803419	1	0.353943	61.1151
**386**	GO:0060346~bone trabecula formation	2	1.709402	0.053957	COL1A1, MMP2	35.8803419	1	0.353943	61.1151
**387**	GO:0048304~positive regulation of isotype switching to IgG isotypes	2	1.709402	0.053957	IL4, IL2	35.8803419	1	0.353943	61.1151
**388**	GO:0097011~cellular response to granulocyte macrophage colony-stimulating factor stimulus	2	1.709402	0.053957	AKT1, MAPK1	35.8803419	1	0.353943	61.1151
**389**	GO:0010544~negative regulation of platelet activation	2	1.709402	0.053957	THBD, NOS3	35.8803419	1	0.353943	61.1151
**390**	GO:0033598~mammary gland epithelial cell proliferation	2	1.709402	0.053957	MAPK1, CCND1	35.8803419	1	0.353943	61.1151
**391**	GO:0031659~positive regulation of cyclin-dependent protein serine/threonine kinase activity involved in G1/S transition of mitotic cell cycle	2	1.709402	0.053957	AKT1, EGFR	35.8803419	1	0.353943	61.1151
**392**	GO:0043497~regulation of protein heterodimerization activity	2	1.709402	0.053957	BAX, BCL2	35.8803419	1	0.353943	61.1151
**393**	GO:0002262~myeloid cell homeostasis	2	1.709402	0.053957	BAX, SOD1	35.8803419	1	0.353943	61.1151
**394**	GO:1901796~regulation of signal transduction by p53 class mediator	4	3.418803	0.054622	AKT1, MAPK14, TP63, MDM2	4.62972153	1	0.356299	61.57769
**395**	GO:0042475~odontogenesis of dentin-containing tooth	3	2.564103	0.055428	BAX, TP63, CA2	7.82843823	1	0.359378	62.13185
**396**	GO:0001570~vasculogenesis	3	2.564103	0.057228	VEGFA, HAS2, RASA1	7.68864469	1	0.367624	63.34222
**397**	GO:0016049~cell growth	3	2.564103	0.057228	AR, IL6, BCL2	7.68864469	1	0.367624	63.34222
**398**	GO:0038096~Fc-gamma receptor signaling pathway involved in phagocytosis	4	3.418803	0.057867	MAPK1, HSP90AA1, MAPK3, PRKCD	4.52035803	1	0.36972	63.76263
**399**	GO:0006959~humoral immune response	3	2.564103	0.059049	IL6, CCL2, BCL2	7.55375619	1	0.374586	64.52911
**400**	GO:0009651~response to salt stress	2	1.709402	0.060496	HSP90AA1, BAX	31.8936372	1	0.380724	65.44668
**401**	GO:0060068~vagina development	2	1.709402	0.060496	BAX, ESR1	31.8936372	1	0.380724	65.44668
**402**	GO:0042473~outer ear morphogenesis	2	1.709402	0.060496	MAPK1, MAPK3	31.8936372	1	0.380724	65.44668
**403**	GO:0002024~diet induced thermogenesis	2	1.709402	0.060496	ADRB2, ADRB1	31.8936372	1	0.380724	65.44668
**404**	GO:0032886~regulation of microtubule-based process	2	1.709402	0.060496	GSK3B, ERBB2	31.8936372	1	0.380724	65.44668
**405**	GO:0010469~regulation of receptor activity	2	1.709402	0.060496	PRKCD, PLAU	31.8936372	1	0.380724	65.44668
**406**	GO:0010224~response to UV-B	2	1.709402	0.060496	RELA, BCL2	31.8936372	1	0.380724	65.44668
**407**	GO:1902894~negative regulation of pri-miRNA transcription from RNA polymerase II promoter	2	1.709402	0.060496	PPARA, PPARD	31.8936372	1	0.380724	65.44668
**408**	GO:0060020~Bergmann glial cell differentiation	2	1.709402	0.060496	MAPK1, MAPK3	31.8936372	1	0.380724	65.44668
**409**	GO:0043589~skin morphogenesis	2	1.709402	0.060496	TP63, COL1A1	31.8936372	1	0.380724	65.44668
**410**	GO:0010838~positive regulation of keratinocyte proliferation	2	1.709402	0.060496	TP63, HAS2	31.8936372	1	0.380724	65.44668
**411**	GO:0097267~omega-hydroxylase P450 pathway	2	1.709402	0.060496	CYP1A1, CYP1A2	31.8936372	1	0.380724	65.44668
**412**	GO:0002544~chronic inflammatory response	2	1.709402	0.060496	VCAM1, GJA1	31.8936372	1	0.380724	65.44668
**413**	GO:0051592~response to calcium ion	3	2.564103	0.060889	EGFR, IL6, CCND1	7.42351901	1	0.381475	65.69213
**414**	GO:0060041~retina development in camera-type eye	3	2.564103	0.060889	IL4, ACHE, BAX	7.42351901	1	0.381475	65.69213
**415**	GO:0045669~positive regulation of osteoblast differentiation	3	2.564103	0.064628	IL6, TP63, GJA1	7.17606838	1	0.398791	67.94533
**416**	GO:0051289~protein homotetramerization	3	2.564103	0.064628	RXRA, TP63, CAT	7.17606838	1	0.398791	67.94533
**417**	GO:0033160~positive regulation of protein import into nucleus, translocation	2	1.709402	0.06699	IL6, HSP90AA1	28.7042735	1	0.408972	69.29596
**418**	GO:1901030~positive regulation of mitochondrial outer membrane permeabilization involved in apoptotic signaling pathway	2	1.709402	0.06699	BAX, GSK3B	28.7042735	1	0.408972	69.29596
**419**	GO:0001893~maternal placenta development	2	1.709402	0.06699	AKT1, RXRA	28.7042735	1	0.408972	69.29596
**420**	GO:0051146~striated muscle cell differentiation	2	1.709402	0.06699	AKT1, MAPK14	28.7042735	1	0.408972	69.29596
**421**	GO:0033327~Leydig cell differentiation	2	1.709402	0.06699	AR, CCND1	28.7042735	1	0.408972	69.29596

422	GO:0046902~regulation of mitochondrial membrane permeability	2	1.709402	0.06699	BCL2, BCL2L1	28.7042735	1	0.408972	69.29596
423	GO:0010996~response to auditory stimulus	2	1.709402	0.06699	IL6, CXCL10	28.7042735	1	0.408972	69.29596
424	GO:0042359~vitamin D metabolic process	2	1.709402	0.06699	CYP3A4, CYP1A1	28.7042735	1	0.408972	69.29596
425	GO:0006977~DNA damage response, signal transduction by p53 class mediator resulting in cell cycle arrest	3	2.564103	0.068442	CDKN1A, BAX, MDM2	6.9445823	1	0.414626	70.09988
426	GO:0001501~skeletal system development	4	3.418803	0.069321	MMP9, TP63, IGF2, COL1A1	4.19040489	1	0.417497	70.57681
427	GO:0009611~response to	3	2.564103	0.070377	ACHE, CCL2, CYP1A1	6.83435083	1	0.421157	71.1398
428	GO:0032436~positive regulation of proteasomal ubiquitin-dependent protein catabolic process	3	2.564103	0.072329	AKT1, GSK3B, MDM2	6.7275641	1	0.428903	72.1547
429	GO:0009743~response to carbohydrate	2	1.709402	0.073439	MDM2, IL1B	26.0947941	1	0.432681	72.71661
430	GO:0031284~positive regulation of guanylate cyclase activity	2	1.709402	0.073439	NOS3, NOS2	26.0947941	1	0.432681	72.71661
431	GO:0051024~positive regulation of immunoglobulin secretion	2	1.709402	0.073439	IL6, IL2	26.0947941	1	0.432681	72.71661
432	GO:0033197~response to vitamin E	2	1.709402	0.073439	CCND1, CAT	26.0947941	1	0.432681	72.71661
433	GO:0061045~negative regulation of wound healing	2	1.709402	0.073439	HMGCR, GJA1	26.0947941	1	0.432681	72.71661
434	GO:0002523~leukocyte migration involved in inflammatory response	2	1.709402	0.073439	CCL2, SELE	26.0947941	1	0.432681	72.71661
435	GO:0031571~mitotic G1 DNA damage checkpoint	2	1.709402	0.073439	CCND1, TP63	26.0947941	1	0.432681	72.71661
436	GO:0002064~epithelial cell development	2	1.709402	0.073439	ESR1, TP63	26.0947941	1	0.432681	72.71661
437	GO:0045945~positive regulation of transcription from RNA polymerase III promoter	2	1.709402	0.073439	AR, ERBB2	26.0947941	1	0.432681	72.71661
438	GO:0030183~B cell differentiation	3	2.564103	0.076285	IL4, VCAM1, CD40LG	6.52369852	1	0.444192	74.10944
439	GO:0050796~regulation of insulin secretion	3	2.564103	0.078289	PRKCA, IL1B, NOS2	6.42632989	1	0.451733	75.04936
440	GO:0045086~positive regulation of interleukin-2 biosynthetic process	2	1.709402	0.079844	IL1B, IL1A	23.9202279	1	0.457188	75.75636
441	GO:0031000~response to caffeine	2	1.709402	0.079844	IL6, PPARG	23.9202279	1	0.457188	75.75636
442	GO:0035413~positive regulation of catenin import into nucleus	2	1.709402	0.079844	EGFR, EGF	23.9202279	1	0.457188	75.75636
443	GO:0001774~microglial cell activation	2	1.709402	0.079844	IL4, JUN	23.9202279	1	0.457188	75.75636
444	GO:0007171~activation of transmembrane receptor protein tyrosine kinase activity	2	1.709402	0.079844	ADRB2, EGF	23.9202279	1	0.457188	75.75636
445	GO:0019725~cellular homeostasis	2	1.709402	0.079844	CCL2, MCL1	23.9202279	1	0.457188	75.75636
446	GO:0043616~keratinocyte proliferation	2	1.709402	0.079844	PPARD, TP63	23.9202279	1	0.457188	75.75636
447	GO:0031669~cellular response to nutrient levels	2	1.709402	0.079844	ICAM1, IL6	23.9202279	1	0.457188	75.75636
448	GO:0015908~fatty acid transport	2	1.709402	0.079844	PPARA, PPARD	23.9202279	1	0.457188	75.75636
449	GO:0070989~oxidative demethylation	2	1.709402	0.079844	CYP3A4, CYP1A2	23.9202279	1	0.457188	75.75636
450	GO:0040015~negative regulation of multicellular organism growth	2	1.709402	0.079844	ADRB2, ADRB1	23.9202279	1	0.457188	75.75636
451	GO:0009268~response to pH	2	1.709402	0.079844	GJA1, CA2	23.9202279	1	0.457188	75.75636
452	GO:0009749~response to glucose	3	2.564103	0.080309	CASP3, PPARD, GJA1	6.33182504	1	0.457881	75.96443
453	GO:0007265~Ras protein signal transduction	3	2.564103	0.084398	CDKN1A, MAPK14, JUN	6.15091575	1	0.473922	77.72062
454	GO:0001878~response to yeast	2	1.709402	0.086205	IL6, MPO	22.0802104	1	0.480095	78.45758
455	GO:0045019~negative regulation of nitric oxide biosynthetic process	2	1.709402	0.086205	OPRM1, IL4	22.0802104	1	0.480095	78.45758
456	GO:0006693~prostaglandin metabolic process	2	1.709402	0.086205	AKR1C3, PTGS2	22.0802104	1	0.480095	78.45758
457	GO:0061029~eyelid development in camera-type eye	2	1.709402	0.086205	EGFR, JUN	22.0802104	1	0.480095	78.45758
458	GO:0046685~response to arsenic-containing substance	2	1.709402	0.086205	CDKN1A, CYP1A1	22.0802104	1	0.480095	78.45758
459	GO:0032026~response to magnesium ion	2	1.709402	0.086205	CCND1, MDM2	22.0802104	1	0.480095	78.45758
460	GO:0060334~regulation of interferon-gamma-mediated signaling pathway	2	1.709402	0.086205	STAT1, IFNGR1	22.0802104	1	0.480095	78.45758
461	GO:0050901~leukocyte tethering or rolling	2	1.709402	0.086205	VCAM1, SELE	22.0802104	1	0.480095	78.45758
462	GO:0045088~regulation of innate immune response	2	1.709402	0.086205	XIAP, IRF1	22.0802104	1	0.480095	78.45758
463	GO:0060425~lung morphogenesis	2	1.709402	0.086205	MAPK1, MAPK3	22.0802104	1	0.480095	78.45758
464	GO:0045651~positive regulation of macrophage differentiation	2	1.709402	0.086205	PRKCA, CASP8	22.0802104	1	0.480095	78.45758
465	GO:0043568~positive regulation of insulin-like growth factor receptor signaling pathway	2	1.709402	0.086205	AR, IGFBP3	22.0802104	1	0.480095	78.45758
466	GO:0019372~lipoxygenase pathway	2	1.709402	0.086205	PTGS2, ALOX5	22.0802104	1	0.480095	78.45758

**467**	GO:0071391~cellular response to estrogen stimulus	2	1.709402	0.086205	ESR1, MDM2	22.0802104	1	0.480095	78.45758
**468**	GO:0002526~acute inflammatory response	2	1.709402	0.086205	VCAM1, IL6	22.0802104	1	0.480095	78.45758
**469**	GO:0050729~positive regulation of inflammatory response	3	2.564103	0.090647	EGFR, CCL2, IL2	5.89813839	1	0.496797	80.17299
**470**	GO:0010875~positive regulation of cholesterol efflux	2	1.709402	0.092522	PON1, NFKBIA	20.5030525	1	0.50289	80.85797
**471**	GO:0001516~prostaglandin biosynthetic process	2	1.709402	0.092522	PTGS2, PTGS1	20.5030525	1	0.50289	80.85797
**472**	GO:0010243~response to organonitrogen compound	2	1.709402	0.092522	CDKN1A, CCND1	20.5030525	1	0.50289	80.85797
**473**	GO:0042176~regulation of protein catabolic process	2	1.709402	0.092522	ODC1, MDM2	20.5030525	1	0.50289	80.85797
**474**	GO:0070498~interleukin-1-mediated signaling pathway	2	1.709402	0.092522	MAPK3, IL1A	20.5030525	1	0.50289	80.85797
**475**	GO:0031103~axon regeneration	2	1.709402	0.092522	JUN, BCL2	20.5030525	1	0.50289	80.85797
**476**	GO:0071354~cellular response to interleukin-6	2	1.709402	0.092522	CCL2, RELA	20.5030525	1	0.50289	80.85797
**477**	GO:1902176~negative regulation of oxidative stress-induced intrinsic apoptotic signaling pathway	2	1.709402	0.092522	AKT1, HSPB1	20.5030525	1	0.50289	80.85797
**478**	GO:0014912~negative regulation of smooth muscle cell migration	2	1.709402	0.092522	PPARD, IGFBP3	20.5030525	1	0.50289	80.85797
**479**	GO:0030216~keratinocyte differentiation	3	2.564103	0.097027	AKR1C3, CASP3, TP63	5.66531714	1	0.519011	82.41335
**480**	GO:0060397~JAK-STAT cascade involved in growth hormone signaling pathway	2	1.709402	0.098796	MAPK1, MAPK3	19.1361823	1	0.524356	82.99102
**481**	GO:0006691~leukotriene metabolic process	2	1.709402	0.098796	LTA4H, ALOX5	19.1361823	1	0.524356	82.99102
**482**	GO:0045725~positive regulation of glycogen biosynthetic process	2	1.709402	0.098796	AKT1, IGF2	19.1361823	1	0.524356	82.99102
**483**	GO:0043122~regulation of I-kappaB kinase/NF-kappaB signaling	2	1.709402	0.098796	HSPB1, IL1B	19.1361823	1	0.524356	82.99102
**484**	GO:0032469~endoplasmic reticulum calcium ion homeostasis	2	1.709402	0.098796	BAX, BCL2	19.1361823	1	0.524356	82.99102
**485**	GO:0042953~lipoprotein transport	2	1.709402	0.098796	PPARG, PRKCB	19.1361823	1	0.524356	82.99102
**486**	GO:0008631~intrinsic apoptotic signaling pathway in response to oxidative stress	2	1.709402	0.098796	BCL2, PRKCD	19.1361823	1	0.524356	82.99102
**487**	GO:0032270~positive regulation of cellular protein metabolic process	2	1.709402	0.098796	AKT1, NFKBIA	19.1361823	1	0.524356	82.99102

AD: atopic dermatitis. OB: oral bioavailability; DL: drug-likeness

Table S5. The gene ontology enrichment analysis for intersection targets between compound (OB < 30% and DL ⩾ 0.18) and AD related targets

	Term	Count	%	PValue	Genes	Fold Enrichment	Bonferroni	Benjamini	FDR
1	GO:0042493~response to drug	24	22.42991	1.01E-18	IL4, ICAM1, IL6, HSP90AA1, PTGS2, CYP1A1, CREB1, RELA, PPARG, SLC6A4, SOD1, STAT1, POR, STAT3, FOS, CASP3, CD86, CCND1, CDKN1A, JUN, BCL2, TBXA2R, MDM2, CAT	12.3895721	1.83E-15	1.83E-15	1.72E-15
2	GO:0045471~response to ethanol	15	14.01869	3.74E-15	IL4, ICAM1, CCND1, CCL2, HMGCR, CASP8, TBXA2R, IL13, CAT, SOD1, HTR3A, NQO1, GSTP1, STAT3, IL2	22.4192256	6.84E-12	3.42E-12	6.42E-12
3	GO:0043066~negative regulation of apoptotic process	24	22.42991	7.36E-15	IL4, IL6, XIAP, MCL1, FLT4, MMP9, RELA, NFKBIA, TP63, BCL2L1, STAT3, AKT1, CASP3, CDKN1A, CD40LG, BCL2, GSK3B, VEGFA, MDM2, MAPK8, CAT, NQO1, GSTP1, IL2	8.27786793	1.33E-11	4.43E-12	1.25E-11
4	GO:0006954~inflammatory response	22	20.56075	2.11E-14	IL6, CCL3, CCL2, C5AR1, PTGER3, PTGS2, C3, RELA, C5, PTGS1, IL13, ITGB2, AKT1, CCL11, FOS, CD40LG, CCR3, IKBKG, TBXA2R, IL1B, MS4A2, SELE	9.10965896	3.82E-11	9.56E-12	3.58E-11
5	GO:0008284~positive regulation of cell proliferation	22	20.56075	1.17E-12	PTPN6, ODC1, CSF2, AR, IL6, FLT4, RELA, FASLG, BCL2L1, STAT3, AKR1C3, MAPK1, CD86, INS, BCL2, F2, VEGFA, MDM2, HAS2, EGF, DPP4, IL2	7.40892864	2.13E-09	4.26E-10	2.00E-09
6	GO:0007568~aging	15	14.01869	2.17E-12	IL6, CCL2, CYP1A1, HMGCR, CREB1, RELA, ITGB2, SOD1, STAT3, AKT1, FOS, CD86, JUN, CAT, NQO1	14.2667799	3.93E-09	6.55E-10	3.69E-09
7	GO:0001666~response to hypoxia	15	14.01869	3.83E-12	CCL2, CYP1A1, CREB1, SLC6A4, MMP2, PRKCB, CASP3, HIF1A, HMOX1, VEGFA, CAT, NOS2, CASP1, PLAU, DPP4	13.6861552	6.94E-09	9.92E-10	6.51E-09
8	GO:0045429~positive regulation of nitric oxide biosynthetic process	10	9.345794	5.31E-12	OPRM1, AKT1, ICAM1, IL6, HSP90AA1, PTGS2, INS, ESR1, IL1B, ITGB2	36.4964138	9.63E-09	1.20E-09	9.03E-09
9	GO:0045766~positive regulation of angiogenesis	13	12.14953	7.61E-12	C5AR1, C3, C5, ITGB2, PRKCB, CCL11, HIF1A, CCR3, HMOX1, VEGFA, TBXA2R, IL1B, NOS3	17.7404307	1.38E-08	1.53E-09	1.29E-08
10	GO:0097192~extrinsic apoptotic signaling pathway in absence of ligand	9	8.411215	3.06E-11	IL4, CASP3, MCL1, BAX, BCL2, GSK3B, IL1B, BCL2L1, IL2	41.5415063	5.54E-08	5.54E-09	5.19E-08
11	GO:0071407~cellular response to organic cyclic compound	10	9.345794	1.09E-10	AKT1, CASP3, CCL3, CCL2, CYP1A1, CASP8, NFKBIA, IL1B, STAT1, STAT3	26.5990813	1.97E-07	1.79E-08	1.85E-07
12	GO:0071222~cellular response to lipopolysaccharide	12	11.21495	1.27E-10	CSF2, ICAM1, IL6, CD86, CCL2, CD80, MAPK14, RELA, TBXA2R, MAPK8, NOS2, GSTP1	16.6656191	2.30E-07	1.92E-08	2.16E-07
13	GO:0032496~response to lipopolysaccharide	13	12.14953	5.00E-10	OPRM1, FOS, CASP3, C5AR1, CYP1A1, PTGS2, JUN, CASP8, IL13, FASLG, IDO1, CASP1, SELE	12.4399362	9.07E-07	6.98E-08	8.50E-07
14	GO:0051092~positive regulation of NF-kappaB transcription factor activity	12	11.21495	7.43E-10	ICAM1, AR, IL6, CD40LG, INS, RELA, IKBKG, NFKBIA, IL1B, ITGB2, CAT, PRKCB	14.1595109	1.35E-06	9.62E-08	1.26E-06
15	GO:0046677~response to antibiotic	8	7.476636	9.66E-10	CASP3, IL6, HSP90AA1, CCL2, CYP1A1, CASP8, MDM2, SOD1	39.2336449	1.75E-06	1.17E-07	1.64E-06
16	GO:0031663~lipopolysaccharide-mediated signaling pathway	8	7.476636	9.66E-10	AKT1, MAPK1, CCL3, CCL2, MAPK14, NFKBIA, IL1B, NOS3	39.2336449	1.75E-06	1.17E-07	1.64E-06
17	GO:0007165~signal transduction	28	26.16822	2.04E-09	CCL2, C3, PPARG, TNFSF15, IL13, FASLG, AKT1, IL1B, HTR3A, EGF, CASP1, IFNGR1, AR, HSP90AA1, C5AR1, CREB1, ESR1, PRKCD, STAT3, PRKCB, CCL11, MAPK1, HIF1A, NR1I2, MAPK14, PECAM1, IL5RA, PLAU	3.78481329	3.70E-06	2.31E-07	3.47E-06
18	GO:0009636~response to toxic substance	10	9.345794	3.12E-09	MAPK1, FOS, CDKN1A, CCL3, BAX, BCL2, SLC6A4, MDM2, NQO1, GSTP1	18.4628917	5.65E-06	3.32E-07	5.30E-06
19	GO:0045893~positive regulation of transcription, DNA-templated	19	17.75701	3.41E-09	IL4, AR, IL6, CREB1, RELA, PPARG, ESR1, TP63, STAT1, STAT3, MAPK1, FOS, CD86, NR1I2, HIF1A, CD80, JUN, IL1B, EGF	5.78981944	6.17E-06	3.43E-07	5.79E-06
20	GO:0032355~response to estradiol	10	9.345794	5.75E-09	CASP3, CCND1, PTGS2, SLC6A4, CASP8, ESR1, CAT, NQO1, GSTP1, STAT3	17.2455582	1.04E-05	5.48E-07	9.77E-06
21	GO:0006955~immune response	17	15.88785	8.60E-09	IL4, CSF2, IL6, CCL3, CCL2, C5AR1, C3, TNFSF15, FASLG, IL13, CCL11, CD86, CD40LG, IKBKG, IL1B, MS4A2, IL2	6.33702577	1.56E-05	7.79E-07	1.46E-05
22	GO:0010628~positive regulation of gene expression	14	13.08411	1.06E-08	CSF2, AR, CCL3, IL6, SLC6A4, STAT3, NR1I2, HIF1A, INS, MAPK14, VEGFA, IL1B, MDM2, MAPK8	8.38581722	1.92E-05	9.12E-07	1.80E-05
23	GO:0010575~positive regulation of vascular endothelial growth factor production	7	6.542056	1.46E-08	HIF1A, C5AR1, PTGS2, C3, FLT4, C5, IL1B	40.6867428	2.64E-05	1.20E-06	2.48E-05
24	GO:0006915~apoptotic process	19	17.75701	1.52E-08	PTPN6, C5AR1, XIAP, NFKBIA, TP63, FASLG, ITGB2, STAT1, PRKCD, PRKCB, MAPK1, CASP3, BCL2, BAX, MAPK14, CASP8, IKBKG, IL1B,	5.2588307	2.75E-05	1.20E-06	2.58E-05
25	GO:0007584~response to nutrient	9	8.411215	2.06E-08	AKR1C3, IL4, HMGCR, SLC6A4, PPARG, TBXA2R, STAT1, NQO1, POR	19.086638	3.73E-05	1.56E-06	3.50E-05
26	GO:0045944~positive regulation of transcription from RNA polymerase II promoter	24	22.42991	3.55E-08	IL4, AR, IL6, CREB1, RXRA, RELA, PPARG, ESR1, NFKBIA, TP63, STAT1, STAT3, AKT1, FOS, ADRB2, NR1I2, HIF1A, JUN, GSK3B, MAPK14, IKBKG, VEGFA, IL1B, IL2	3.83937809	6.44E-05	2.58E-06	6.04E-05
27	GO:0007267~cell-cell signaling	13	12.14953	6.92E-08	AR, CD86, ADRB2, CCL3, ADRB1, CD80, INS, ADRA1B, FASLG, IL13, IL1B, ITGB2, IL2	8.03208477	1.25E-04	4.82E-06	1.18E-04
28	GO:0006935~chemotaxis	10	9.345794	7.61E-08	CCL11, IL4, MAPK1, CCL3, CCL2, C5AR1, MAPK14, CCR3, C5, PLAU	12.8634901	1.38E-04	5.11E-06	1.29E-04
29	GO:0009409~response to cold	7	6.542056	9.16E-08	FOS, IL6, ADRB2, HSP90AA1, ADRB1, CASP8, PPARG	30.5150571	1.66E-04	5.93E-06	1.56E-04
30	GO:0048661~positive regulation of smooth muscle cell proliferation	8	7.476636	9.59E-08	AKT1, IL6, PTGS2, HMGCR, JUN, HMOX1, IL13, STAT1	20.9246106	1.74E-04	5.99E-06	1.63E-04
31	GO:0070374~positive regulation of ERK1 and ERK2 cascade	11	10.28037	1.57E-07	OPRM1, CCL11, ICAM1, IL6, CCL3, CCL2, C5AR1, HMGCR, JUN, FLT4, VEGFA	9.86445928	2.85E-04	9.49E-06	2.67E-04

**32**	GO:0001938~positive regulation of endothelial cell proliferation	8	7.476636	2.56E-07	CCL11, AKT1, HIF1A, CCL2, JUN, FLT4, CCR3, VEGFA	18.1953136	4.63E-04	1.50E-05	4.35E-04
**33**	GO:0071347~cellular response to interleukin-1	8	7.476636	3.12E-07	CCL11, ICAM1, IL6, CCL3, HIF1A, CCL2, RELA, HAS2	17.6827695	5.65E-04	1.77E-05	5.30E-04
**34**	GO:0051090~regulation of sequence-specific DNA binding transcription factor activity	6	5.607477	4.38E-07	MAPK1, FOS, MAPK14, JUN, HMOX1, MAPK8	37.6642991	7.93E-04	2.40E-05	7.44E-04
**35**	GO:0042542~response to hydrogen peroxide	7	6.542056	7.85E-07	CASP3, JUN, HMOX1, BCL2, CAT, SOD1, STAT1	21.5400403	0.0014206	4.18E-05	0.001333
**36**	GO:0034097~response to cytokine	7	6.542056	8.82E-07	IL4, FOS, MCL1, JUN, BCL2, BCL2L1, STAT1	21.1258088	0.0015976	4.57E-05	0.0015
**37**	GO:0050900~leukocyte migration	9	8.411215	1.03E-06	ICAM1, PTPN6, C5AR1, MMP9, PECAM1, F2, ITGB2, SELE, MMP1	11.5771411	0.0018567	5.16E-05	0.001743
**38**	GO:0043200~response to amino acid	6	5.607477	1.36E-06	ICAM1, CASP3, IL6, CCL2, RELA, GSTP1	30.3744347	0.0024596	6.66E-05	0.00231
**39**	GO:0001525~angiogenesis	11	10.28037	1.46E-06	HIF1A, CCL2, PTGS2, MAPK14, JUN, HMOX1, PECAM1, VEGFA, NOS3, EGF, MMP2	7.7411676	0.0026442	6.97E-05	0.002483
**40**	GO:0071456~cellular response to hypoxia	8	7.476636	2.46E-06	AKT1, ICAM1, HIF1A, PTGS2, HMOX1, BCL2, VEGFA, MDM2	13.0778816	0.0044447	1.14E-04	0.004178
**41**	GO:0035994~response to muscle stretch	5	4.672897	2.58E-06	FOS, MAPK14, RELA, JUN, NFKBIA	49.0420561	0.0046554	1.17E-04	0.004376
**42**	GO:0043627~response to estrogen	7	6.542056	3.35E-06	MAPK1, CCND1, HSP90AA1, HMOX1, PPARG, ESR1, CA2	16.900647	0.0060523	1.48E-04	0.005694
**43**	GO:0060749~mammary gland alveolus development	5	4.672897	3.35E-06	AR, CCND1, VEGFA, ESR1, EGF	46.1572292	0.006054	1.45E-04	0.005695
**44**	GO:2001240~negative regulation of extrinsic apoptotic signaling pathway in absence of ligand	6	5.607477	3.38E-06	AKT1, CSF2, MCL1, BCL2, IL1B, BCL2L1	25.4488507	0.0061119	1.43E-04	0.00575
**45**	GO:0018107~peptidyl-threonine phosphorylation	6	5.607477	3.88E-06	AKT1, MAPK1, BCL2, GSK3B, MAPK8, PRKCD	24.7791441	0.0069996	1.60E-04	0.006588
**46**	GO:0055114~oxidation-reduction process	16	14.95327	4.52E-06	CYP3A4, PTGS2, CYP1A1, HMGCR, MAOA, PTGS1, IDO1, SOD1, POR, AKR1C3, FASN, NOS3, ALOX5, NOS2, NQO1, CYP19A1	4.24147512	0.0081594	1.82E-04	0.007684
**47**	GO:1901215~negative regulation of neuron death	6	5.607477	5.03E-06	AKT1, IL6, CREB1, IKBKG, IL13, STAT3	23.5401869	0.009075	1.98E-04	0.00855
**48**	GO:0000187~activation of MAPK activity	8	7.476636	5.08E-06	MAPK1, C5AR1, MAPK14, C5, IKBKG, IL1B, SOD1, EGF	11.7334265	0.0091657	1.96E-04	0.008636
**49**	GO:0071260~cellular response to mechanical stimulus	7	6.542056	5.63E-06	AKT1, PTGS2, CASP8, IL1B, IL13, MAPK8, CASP1	15.4724233	0.0101568	2.13E-04	0.009574
**50**	GO:0007566~embryo implantation	6	5.607477	6.44E-06	PTGS2, RXRA, MMP9, IL1B, SOD1, MMP2	22.4192256	0.0116003	2.38E-04	0.010943
**51**	GO:0001541~ovarian follicle development	6	5.607477	6.44E-06	ICAM1, BAX, BCL2, VEGFA, BCL2L1, SOD1	22.4192256	0.0116003	2.38E-04	0.010943
**52**	GO:1902895~positive regulation of pri-miRNA transcription from RNA polymerase II promoter	5	4.672897	6.72E-06	FOS, HIF1A, RELA, JUN, STAT3	39.2336449	0.0121089	2.44E-04	0.011426
**53**	GO:0043525~positive regulation of neuron apoptotic process	6	5.607477	7.25E-06	CASP3, CCL3, JUN, BAX, FASLG, NQO1	21.8978483	0.0130515	2.58E-04	0.012321
**54**	GO:0030168~platelet activation	8	7.476636	8.19E-06	AKT1, MAPK1, PTPN6, IL6, CD40LG, F2, PRKCD, PRKCB	10.9171881	0.0147367	2.85E-04	0.013924
**55**	GO:0031295~T cell costimulation	7	6.542056	9.75E-06	AKT1, PTPN6, CD86, CD80, CD40LG, CD247, DPP4	14.0838725	0.0175115	3.33E-04	0.016568
**56**	GO:0051000~positive regulation of nitric-oxide synthase activity	5	4.672897	1.01E-05	AKT1, HIF1A, INS, FCER2, ESR1	35.6669499	0.0180508	3.37E-04	0.017083
**57**	GO:0009408~response to heat	6	5.607477	1.26E-05	AKT1, IL6, HSP90AA1, CCL2, NOS3, SOD1	19.6168224	0.0225339	4.14E-04	0.021374
**58**	GO:0002674~negative regulation of acute inflammatory response	4	3.738318	1.34E-05	IL4, INS, PPARG, GSTP1	78.4672897	0.0239544	4.33E-04	0.022738
**59**	GO:0006919~activation of cysteine-type endopeptidase activity involved in apoptotic process	7	6.542056	1.40E-05	CASP3, BAX, CASP8, PPARG, TNFSF15, FASLG, CASP1	13.2354465	0.0249906	4.44E-04	0.023734
**60**	GO:0018105~peptidyl-serine phosphorylation	8	7.476636	1.42E-05	AKT1, MAPK1, MAPK14, BCL2, GSK3B, MAPK8, PRKCD, PRKCB	10.0438131	0.0253365	4.42E-04	0.024067
**61**	GO:0001934~positive regulation of protein phosphorylation	8	7.476636	1.57E-05	AKT1, CCND1, C3, FLT4, MMP9, VEGFA, F2, IL1B	9.8856428	0.0280616	4.82E-04	0.026692
**62**	GO:0006508~proteolysis	14	13.08411	1.58E-05	C3, MMP9, MMP3, MMP2, MMP1, MMP10, CASP3, BACE1, CASP8, F2, LTA4H, CASP1, DPP4, PLAU	4.39416822	0.0281535	4.76E-04	0.026781
**63**	GO:0043524~negative regulation of neuron apoptotic process	8	7.476636	2.02E-05	CCL2, C5AR1, JUN, HMOX1, BAX, BCL2, BCL2L1, SOD1	9.51118663	0.0359234	6.00E-04	0.034307
**64**	GO:0007204~positive regulation of cytosolic calcium ion concentration	8	7.476636	2.23E-05	OPRM1, PTGER3, C5AR1, CCR3, ADRA1B, TBXA2R, ESR1, IL2	9.36922862	0.0395256	6.50E-04	0.037817
**65**	GO:0008360~regulation of cell shape	8	7.476636	2.95E-05	CCL11, ICAM1, IL6, CCL3, CCL2, VEGFA, F2, ITGB2	8.96769025	0.052095	8.49E-04	0.050167
**66**	GO:0043542~endothelial cell migration	5	4.672897	3.15E-05	PECAM1, NOS3, ITGB2, STAT1, DPP4	27.0576861	0.0555649	8.93E-04	0.053605
**67**	GO:0042593~glucose homeostasis	7	6.542056	4.28E-05	AKT1, IL6, HIF1A, INS, PPARG, ADRA1B, STAT3	10.876654	0.0746044	0.001192	0.072694
**68**	GO:0045907~positive regulation of vasoconstriction	5	4.672897	4.71E-05	AKT1, ICAM1, PTGS2, ADRA1B, TBXA2R	24.521028	0.0817678	0.001292	0.079977
**69**	GO:0050727~regulation of inflammatory response	6	5.607477	4.79E-05	XIAP, PTGS2, RELA, ESR1, CASP1, SELE	14.9461504	0.0831365	0.001295	0.081375
**70**	GO:1902042~negative regulation of extrinsic apoptotic signaling pathway via death domain receptors	5	4.672897	5.33E-05	ICAM1, HMOX1, CASP8, FASLG, NOS3	23.7779666	0.0920824	0.00142	0.090563
**71**	GO:0008217~regulation of blood pressure	6	5.607477	5.57E-05	PTGS2, HMOX1, PTGS1, PPARG, NOS3, SOD1	14.4862689	0.0960528	0.001462	0.09467
**72**	GO:0030593~neutrophil	6	5.607477	6.00E-05	CCL11, CCL3, CCL2, C5AR1, IL1B, ITGB2	14.2667799	0.1030161	0.001552	0.101916
**73**	GO:0006809~nitric oxide biosynthetic process	4	3.738318	6.68E-05	AKT1, NOS3, NOS2, NQO1	48.2875629	0.1139776	0.001703	0.113435
**74**	GO:0071356~cellular response to tumor necrosis factor	7	6.542056	6.91E-05	CCL11, ICAM1, IL6, CCL3, CCL2, RELA, HAS2	9.98674596	0.1176545	0.001737	0.117331

**75**	GO:0043154~negative regulation of cysteine-type endopeptidase activity involved in apoptotic process	6	5.607477	7.43E-05	AKT1, IL6, XIAP, VEGFA, MDM2, POR	13.6464852	0.1260074	0.001843	0.126242
**76**	GO:0043407~negative regulation of MAP kinase activity	5	4.672897	7.56E-05	PTPN6, HMGCR, IL1B, PRKCD, GSTP1	21.7964694	0.1280758	0.00185	0.128461
**77**	GO:0035094~response to nicotine	5	4.672897	8.44E-05	CASP3, HMOX1, CREB1, BCL2, IL13	21.2073756	0.141804	0.002037	0.143326
**78**	GO:0035729~cellular response to hepatocyte growth factor stimulus	4	3.738318	8.46E-05	IL6, RELA, CREB1, GSK3B	44.8384513	0.1421373	0.002015	0.143689
**79**	GO:0030890~positive regulation of B cell proliferation	5	4.672897	1.04E-04	IL4, CDKN1A, BCL2, IL13, IL2	20.1198179	0.1718918	0.002447	0.176745
**80**	GO:0043065~positive regulation of apoptotic process	10	9.345794	1.19E-04	AKT1, IL6, PTGS2, HMOX1, CREB1, BAX, FASLG, MAPK8, BCL2L1, SOD1	5.23115265	0.1934186	0.002752	0.201402
**81**	GO:0030949~positive regulation of vascular endothelial growth factor receptor signaling pathway	4	3.738318	1.29E-04	HIF1A, VEGFA, IL1B, PRKCB	39.2336449	0.2084083	0.002954	0.218959
**82**	GO:0006805~xenobiotic metabolic process	6	5.607477	1.33E-04	CYP3A4, NR1I2, PTGS1, NQO1, GSTP1, POR	12.0718907	0.2148561	0.003019	0.226613
**83**	GO:0038095~Fc-epsilon receptor signaling pathway	8	7.476636	1.35E-04	MAPK1, FOS, RELA, JUN, IKBKG, NFKBIA, MS4A2, MAPK8	7.05323953	0.2167012	0.003011	0.228815
**84**	GO:0030224~monocyte differentiation	4	3.738318	1.56E-04	CSF2, JUN, VEGFA, PPARG	36.9257834	0.2460978	0.003439	0.264603
**85**	GO:0042523~positive regulation of tyrosine phosphorylation of Stat5 protein	4	3.738318	1.56E-04	IL4, CSF2, PECAM1, IL2	36.9257834	0.2460978	0.003439	0.264603
**86**	GO:0043410~positive regulation of MAPK cascade	6	5.607477	1.60E-04	AR, IL6, ADRB2, INS, FLT4, ADRA1B	11.6247837	0.2511462	0.003478	0.270888
**87**	GO:0008630~intrinsic apoptotic signaling pathway in response to DNA damage	5	4.672897	2.17E-04	MCL1, HMOX1, BAX, BCL2, BCL2L1	16.695168	0.3253035	0.004673	0.368382
**88**	GO:0031100~organ regeneration	5	4.672897	2.17E-04	CDKN1A, CCL2, C5AR1, PPARG, GSTP1	16.695168	0.3253035	0.004673	0.368382
**89**	GO:0008637~apoptotic mitochondrial changes	4	3.738318	2.20E-04	AKT1, MCL1, BAX, BCL2L1	33.0388588	0.3289307	0.004682	0.373419
**90**	GO:0000165~MAPK cascade	9	8.411215	2.55E-04	CSF2, MAPK1, CCL3, CCL2, INS, IL1B, IL5RA, EGF, IL2	5.3908825	0.3697771	0.005354	0.432081
**91**	GO:0010165~response to X-ray	4	3.738318	3.45E-04	CDKN1A, CASP3, CCND1, TP63	28.5335599	0.4648986	0.007162	0.58476
**92**	GO:2001243~negative regulation of intrinsic apoptotic signaling pathway	4	3.738318	3.45E-04	MCL1, BCL2, MMP9, BCL2L1	28.5335599	0.4648986	0.007162	0.58476
**93**	GO:0006974~cellular response to DNA damage stimulus	8	7.476636	3.50E-04	AKT1, MAPK1, CDKN1A, CCND1, XIAP, BCL2, IKBKG, TP63	6.03594536	0.4694641	0.007177	0.592749
**94**	GO:0031622~positive regulation of fever generation	3	2.803738	3.90E-04	PTGER3, PTGS2, IL1B	94.1607477	0.5066787	0.007908	0.660533
**95**	GO:0035924~cellular response to vascular endothelial growth factor stimulus	4	3.738318	3.95E-04	AKT1, MAPK14, FLT4, VEGFA	27.2929703	0.5112087	0.007922	0.669128
**96**	GO:0001836~release of cytochrome c from mitochondria	4	3.738318	3.95E-04	JUN, BAX, BCL2, BCL2L1	27.2929703	0.5112087	0.007922	0.669128
**97**	GO:0042981~regulation of apoptotic process	8	7.476636	4.04E-04	MCL1, BAX, CASP8, ESR1, TP63, BCL2L1, STAT1, CASP1	5.8942565	0.5187447	0.008005	0.683603
**98**	GO:0018108~peptidyl-tyrosine phosphorylation	7	6.542056	4.18E-04	CSF2, PTPN6, HSP90AA1, FLT4, IL5RA, EGF, PRKCD	7.18001344	0.5313934	0.008205	0.70841
**99**	GO:0007159~leukocyte cell-cell adhesion	4	3.738318	5.08E-04	ICAM1, CD40LG, ITGB2, SELE	25.1095327	0.6019585	0.009856	0.860283
**100**	GO:0034612~response to tumor necrosis factor	4	3.738318	5.08E-04	CASP3, PTGS2, CASP8, SELE	25.1095327	0.6019585	0.009856	0.860283
**101**	GO:0008283~cell proliferation	10	9.345794	5.15E-04	AKT1, PTPN6, AR, ACHE, CYP1A1, BCL2, ADRA1B, BCL2L1, IL5RA, STAT3	4.28783004	0.6068386	0.009882	0.871753
**102**	GO:0051091~positive regulation of sequence-specific DNA binding transcription factor activity	6	5.607477	5.34E-04	IL4, AKT1, IL6, PPARG, ESR1, IL1B	8.96769025	0.6198946	0.010131	0.903147
**103**	GO:0043123~positive regulation of I-kappaB kinase/NF-kappaB signaling	7	6.542056	5.48E-04	RELA, HMOX1, CASP8, IKBKG, FASLG, CASP1, PRKCB	6.82324258	0.6296244	0.010293	0.927245
**104**	GO:0050999~regulation of nitric-oxide synthase activity	4	3.738318	5.72E-04	AKT1, HSP90AA1, IL1B, NOS3	24.1437815	0.6453504	0.01063	0.967553
**105**	GO:0090336~positive regulation of brown fat cell differentiation	3	2.803738	5.82E-04	PTGS2, INS, MAPK14	78.4672897	0.6520244	0.010714	0.985198
**106**	GO:0016032~viral process	9	8.411215	6.14E-04	MAPK1, CREB1, BAX, CCR3, CD247, NFKBIA, MDM2, MMP1, STAT3	4.72378333	0.6711041	0.01117	1.037553
**107**	GO:0030574~collagen catabolic process	5	4.672897	7.12E-04	MMP10, MMP9, MMP3, MMP2, MMP1	12.260514	0.7251357	0.012832	1.203988
**108**	GO:0051881~regulation of mitochondrial membrane potential	4	3.738318	7.14E-04	BAX, BCL2, BCL2L1, SOD1	22.4192256	0.7259163	0.012733	1.206623
**109**	GO:0009314~response to radiation	4	3.738318	7.14E-04	OPRM1, CCL11, JUN, BCL2	22.4192256	0.7259163	0.012733	1.206623
**110**	GO:0060326~cell chemotaxis	5	4.672897	7.56E-04	CCL3, CCL2, C5AR1, C5, PRKCD	12.0718907	0.7457838	0.013337	1.276323
**111**	GO:0046627~negative regulation of insulin receptor signaling pathway	4	3.738318	7.93E-04	RELA, IL1B, PRKCD, PRKCB	21.6461489	0.76239	0.013856	1.338855
**112**	GO:0042113~B cell activation	4	3.738318	7.93E-04	IL4, CD86, CASP8, PRKCB	21.6461489	0.76239	0.013856	1.338855
**113**	GO:0045909~positive regulation of vasodilation	4	3.738318	7.93E-04	INS, HMOX1, NOS3, NOS2	21.6461489	0.76239	0.013856	1.338855
**114**	GO:0007200~phospholipase C-activating G-protein coupled receptor signaling pathway	5	4.672897	8.00E-04	OPRM1, PTGER3, C5AR1, ADRA1B, ESR1	11.8889833	0.7656514	0.013855	1.351644
**115**	GO:0071312~cellular response to alkaloid	3	2.803738	8.12E-04	ICAM1, MDM2, BCL2L1	67.2576769	0.7705224	0.013921	1.371076
**116**	GO:2000379~positive regulation of reactive oxygen species metabolic process	4	3.738318	8.77E-04	AKR1C3, CDKN1A, MAPK14, F2	20.9246106	0.7959878	0.014884	1.479826

**117**	GO:1900740~positive regulation of protein insertion into mitochondrial membrane involved in apoptotic signaling pathway	4	3.738318	8.77E-04	BCL2, CASP8, TP63, MAPK8	20.9246106	0.7959878	0.014884	1.479826
**118**	GO:0043547~positive regulation of GTPase activity	12	11.21495	8.78E-04	CCL11, CSF2, ICAM1, CCL3, CCL2, ADRB1, JUN, GSK3B, TBXA2R, IL5RA, EGF, IL2	3.33312381	0.7963093	0.014761	1.481283
**119**	GO:0008285~negative regulation of cell proliferation	10	9.345794	9.02E-04	OPRM1, PTPN6, AR, CDKN1A, IL6, PTGS2, JUN, IL1B, NOS3, STAT3	3.96299443	0.8051984	0.015032	1.522505
**120**	GO:0010332~response to gamma radiation	4	3.738318	9.66E-04	CCL2, BAX, BCL2, TP63	20.2496232	0.8265604	0.015944	1.629729
**121**	GO:0033138~positive regulation of peptidyl-serine phosphorylation	5	4.672897	9.99E-04	AKT1, IL6, BCL2, GSK3B, VEGFA	11.2096128	0.8364766	0.016327	1.684032
**122**	GO:0042220~response to cocaine	4	3.738318	0.001061	OPRM1, HSP90AA1, MDM2, HTR3A	19.6168224	0.85404	0.017188	1.788751
**123**	GO:0030335~positive regulation of cell migration	7	6.542056	0.0011	CCL11, MAPK1, CCL3, INS, VEGFA, HAS2, PLAU	5.97033726	0.8638459	0.017646	1.85279
**124**	GO:0048010~vascular endothelial growth factor receptor signaling pathway	5	4.672897	0.00111	HSP90AA1, CCL2, MAPK14, FLT4, VEGFA	10.8982347	0.8663332	0.017651	1.869761
**125**	GO:0051402~neuron apoptotic process	4	3.738318	0.001162	BAX, BCL2, TP63, BCL2L1	19.0223733	0.878435	0.018315	1.957067
**126**	GO:0043491~protein kinase B signaling	4	3.738318	0.001162	AKT1, CCL3, CCL2, IL1B	19.0223733	0.878435	0.018315	1.957067
**127**	GO:0007623~circadian rhythm	5	4.672897	0.001293	JUN, CREB1, GSK3B, SLC6A4, NOS2	10.4623053	0.9040212	0.020173	2.174138
**128**	GO:0022617~extracellular matrix disassembly	5	4.672897	0.001358	MMP10, MMP9, MMP3, MMP2, MMP1	10.3246434	0.9147379	0.021001	2.282709
**129**	GO:0032930~positive regulation of superoxide anion generation	3	2.803738	0.001381	SOD1, PRKCD, GSTP1	52.3115265	0.9181864	0.021169	2.320542
**130**	GO:0034405~response to fluid shear stress	3	2.803738	0.001381	AKT1, CSF2, NOS3	52.3115265	0.9181864	0.021169	2.320542
**131**	GO:0032869~cellular response to insulin stimulus	5	4.672897	0.001425	AKT1, CCL2, PPARG, STAT1, GSTP1	10.1905571	0.9245695	0.021665	2.394934
**132**	GO:0014066~regulation of phosphatidylinositol 3-kinase signaling	5	4.672897	0.001495	AKT1, MAPK1, CD86, CD80, EGF	10.0599089	0.933547	0.022526	2.510867
**133**	GO:0010507~negative regulation of autophagy	4	3.738318	0.0015	AKT1, MCL1, BCL2, BCL2L1	17.4371755	0.9341653	0.022417	2.519415
**134**	GO:0042060~wound healing	5	4.672897	0.001642	OPRM1, CASP3, IL6, INS, PECAM1	9.80841121	0.9490846	0.024308	2.754086
**135**	GO:0046902~regulation of mitochondrial membrane permeability	3	2.803738	0.001719	BCL2, BCL2L1, STAT3	47.0803738	0.9557008	0.025224	2.880964
**136**	GO:0045080~positive regulation of chemokine biosynthetic process	3	2.803738	0.001719	IL4, HMOX1, IL1B	47.0803738	0.9557008	0.025224	2.880964
**137**	GO:0019371~cyclooxygenase pathway	3	2.803738	0.001719	AKR1C3, PTGS2, PTGS1	47.0803738	0.9557008	0.025224	2.880964
**138**	GO:2001237~negative regulation of extrinsic apoptotic signaling pathway	4	3.738318	0.001757	IL4, AR, RELA, GSTP1	16.5194294	0.9586462	0.025567	2.943616
**139**	GO:0010629~negative regulation of gene expression	6	5.607477	0.001761	AKT1, CDKN1A, CCL3, CREB1, ESR1, NOS2	6.87304727	0.9590072	0.025432	2.951597
**140**	GO:0050731~positive regulation of peptidyl-tyrosine phosphorylation	5	4.672897	0.001798	ICAM1, IL6, CD80, INS, VEGFA	9.56918167	0.9616738	0.025755	3.012801
**141**	GO:0006953~acute-phase response	4	3.738318	0.001894	IL6, INS, F2, STAT3	16.0958543	0.9677928	0.026898	3.170901
**142**	GO:0030307~positive regulation of cell growth	5	4.672897	0.001965	AKT1, INS, BCL2, F2, IL2	9.34134401	0.9716735	0.027673	3.287434
**143**	GO:0051262~protein tetramerization	4	3.738318	0.002038	ACHE, HMGCR, TP63, CAT	15.6934579	0.9752137	0.028473	3.408461
**144**	GO:0043124~negative regulation of I-kappaB kinase/NF-kappaB signaling	4	3.738318	0.002038	CASP8, ESR1, STAT1, GSTP1	15.6934579	0.9752137	0.028473	3.408461
**145**	GO:0002523~leukocyte migration involved in inflammatory response	3	2.803738	0.002092	CCL2, ITGB2, SELE	42.8003398	0.9775047	0.028986	3.496281
**146**	GO:0032495~response to muramyl dipeptide	3	2.803738	0.002092	MAPK14, RELA, NFKBIA	42.8003398	0.9775047	0.028986	3.496281
**147**	GO:0001822~kidney development	5	4.672897	0.002142	ODC1, BAX, VEGFA, HAS2, CA2	9.12410346	0.9794563	0.029444	3.578389
**148**	GO:0007595~lactation	4	3.738318	0.002347	CCND1, HIF1A, CREB1, VEGFA	14.9461504	0.9858474	0.03198	3.914809
**149**	GO:0002548~monocyte chemotaxis	4	3.738318	0.002347	CCL11, IL6, CCL3, CCL2	14.9461504	0.9858474	0.03198	3.914809
**150**	GO:0045740~positive regulation of DNA replication	4	3.738318	0.002347	CSF2, IL6, INS, JUN	14.9461504	0.9858474	0.03198	3.914809
**151**	GO:0006468~protein phosphorylation	10	9.345794	0.002381	CCL11, AKT1, MAPK1, CCND1, CCL2, CREB1, GSK3B, MAPK8, PRKCD, PRKCB	3.44154779	0.9866996	0.032197	3.970765
**152**	GO:0071498~cellular response to fluid shear stress	3	2.803738	0.0025	PTGS2, HAS2, CA2	39.2336449	0.9892789	0.033527	4.164735
**153**	GO:0001774~microglial cell activation	3	2.803738	0.0025	IL4, JUN, IL13	39.2336449	0.9892789	0.033527	4.164735
**154**	GO:0019430~removal of superoxide radicals	3	2.803738	0.0025	NOS3, SOD1, NQO1	39.2336449	0.9892789	0.033527	4.164735
**155**	GO:0045086~positive regulation of interleukin-2 biosynthetic process	3	2.803738	0.0025	CD86, CD80, IL1B	39.2336449	0.9892789	0.033527	4.164735
**156**	GO:0046889~positive regulation of lipid biosynthetic process	3	2.803738	0.0025	AKT1, INS, CREB1	39.2336449	0.9892789	0.033527	4.164735
**157**	GO:0008202~steroid metabolic process	4	3.738318	0.002512	AKR1C3, CYP3A4, NR1I2, CYP1A1	14.5985655	0.9895036	0.033434	4.183776
**158**	GO:0051926~negative regulation of calcium ion transport	3	2.803738	0.002942	ICAM1, PTGS2, NOS3	36.2156722	0.995201	0.038779	4.884514
**159**	GO:0071318~cellular response to ATP	3	2.803738	0.002942	CCL2, PTGS2, SOD1	36.2156722	0.995201	0.038779	4.884514

**160**	GO:0097296~activation of cysteine-type endopeptidase activity involved in apoptotic signaling pathway	3	2.803738	0.002942	BAX, CASP8, FASLG	36.2156722	0.995201	0.038779	4.884514
**161**	GO:0051591~response to cAMP	4	3.738318	0.003047	FOS, RELA, JUN, STAT1	13.6464852	0.9960352	0.039848	5.054717
**162**	GO:0042110~T cell activation	4	3.738318	0.00324	CD86, CD80, CASP8, DPP4	13.3561344	0.9972082	0.042019	5.366564
**163**	GO:2000353~positive regulation of endothelial cell apoptotic process	3	2.803738	0.003419	AKR1C3, CD40LG, FASLG	33.6288385	0.9979809	0.043969	5.653753
**164**	GO:0019395~fatty acid oxidation	3	2.803738	0.003419	MAPK14, PPARG, POR	33.6288385	0.9979809	0.043969	5.653753
**165**	GO:0032461~positive regulation of protein oligomerization	3	2.803738	0.003419	BAX, MMP3, MMP1	33.6288385	0.9979809	0.043969	5.653753
**166**	GO:1904707~positive regulation of vascular smooth muscle cell proliferation	3	2.803738	0.003928	MMP9, MDM2, MMP2	31.3869159	0.999201	0.050017	6.47054
**167**	GO:0032270~positive regulation of cellular protein metabolic process	3	2.803738	0.003928	AKT1, INS, NFKBIA	31.3869159	0.999201	0.050017	6.47054
**168**	GO:0035556~intracellular signal transduction	9	8.411215	0.004004	AKT1, PTPN6, CD80, MAPK14, HMOX1, GSK3B, ADRA1B, PRKCD, PRKCB	3.50474247	0.9993042	0.050606	6.591733
**169**	GO:0032880~regulation of protein localization	4	3.738318	0.004317	AKT1, INS, BCL2, MAPK8	12.0718907	0.999606	0.05408	7.088765
**170**	GO:0097194~execution phase of apoptosis	3	2.803738	0.004471	AKT1, CASP3, CASP8	29.4252336	0.9997024	0.055578	7.332926
**171**	GO:0008209~androgen metabolic process	3	2.803738	0.004471	CYP3A4, ESR1, CYP19A1	29.4252336	0.9997024	0.055578	7.332926
**172**	GO:0050853~B cell receptor signaling pathway	4	3.738318	0.004801	MAPK1, PTPN6, BCL2, PRKCB	11.6247837	0.9998369	0.059163	7.853825
**173**	GO:0055093~response to	3	2.803738	0.005046	CDKN1A, CYP1A1, CAT	27.6943375	0.9998956	0.061678	8.238927
**174**	GO:0042789~mRNA transcription from RNA polymerase II promoter	3	2.803738	0.005046	HIF1A, C5AR1, STAT3	27.6943375	0.9998956	0.061678	8.238927
**175**	GO:2000811~negative regulation of anoikis	3	2.803738	0.005046	MCL1, BCL2, BCL2L1	27.6943375	0.9998956	0.061678	8.238927
**176**	GO:0071276~cellular response to cadmium ion	3	2.803738	0.005046	AKR1C3, HMOX1, SOD1	27.6943375	0.9998956	0.061678	8.238927
**177**	GO:0071310~cellular response to organic substance	3	2.803738	0.005046	BCL2, IL1B, CASP1	27.6943375	0.9998956	0.061678	8.238927
**178**	GO:0009615~response to virus	5	4.672897	0.00519	CCL11, ODC1, CYP1A1, IKBKG, IFNGR1	7.13338997	0.9999196	0.062954	8.46325
**179**	GO:0050776~regulation of immune response	6	5.607477	0.005417	IL4, ICAM1, C3, CD40LG, CD247, ITGB2	5.28992964	0.9999468	0.065189	8.817755
**180**	GO:0032091~negative regulation of protein binding	4	3.738318	0.005587	BAX, GSK3B, MAPK8, PRKCD	11.0129529	0.999961	0.066732	9.082577
**181**	GO:0071346~cellular response to interferon-gamma	4	3.738318	0.005587	CCL11, CCL3, CCL2, NOS2	11.0129529	0.999961	0.066732	9.082577
**182**	GO:0043401~steroid hormone mediated signaling pathway	4	3.738318	0.005587	NR1I2, RXRA, PPARG, ESR1	11.0129529	0.999961	0.066732	9.082577
**183**	GO:0070301~cellular response to hydrogen peroxide	4	3.738318	0.005587	IL6, RELA, MDM2, PRKCD	11.0129529	0.999961	0.066732	9.082577
**184**	GO:0033189~response to vitamin A	3	2.803738	0.005654	CYP1A1, PPARG, CAT	26.1557632	0.9999655	0.067065	9.186533
**185**	GO:0036120~cellular response to platelet-derived growth factor stimulus	3	2.803738	0.005654	CCL2, CREB1, HAS2	26.1557632	0.9999655	0.067065	9.186533
**186**	GO:0051412~response to corticosterone	3	2.803738	0.005654	FOS, CDKN1A, CCND1	26.1557632	0.9999655	0.067065	9.186533
**187**	GO:0010039~response to iron ion	3	2.803738	0.005654	CCND1, BCL2, MDM2	26.1557632	0.9999655	0.067065	9.186533
**188**	GO:0030225~macrophage differentiation	3	2.803738	0.005654	MMP9, CASP8, VEGFA	26.1557632	0.9999655	0.067065	9.186533
**189**	GO:0009612~response to mechanical stimulus	4	3.738318	0.006151	CCL2, JUN, PPARG, STAT1	10.6396325	0.9999861	0.072291	9.955324
**190**	GO:0046716~muscle cell cellular homeostasis	3	2.803738	0.006293	IL6, HIF1A, SOD1	24.7791441	0.9999892	0.073428	10.17371
**191**	GO:0071880~adenylate cyclase-activating adrenergic receptor signaling pathway	3	2.803738	0.006293	ADRB2, ADRB1, ADRA1B	24.7791441	0.9999892	0.073428	10.17371
**192**	GO:0045672~positive regulation of osteoclast differentiation	3	2.803738	0.006293	FOS, CREB1, CA2	24.7791441	0.9999892	0.073428	10.17371
**193**	GO:0046827~positive regulation of protein export from nucleus	3	2.803738	0.006293	GSK3B, MDM2, IL1B	24.7791441	0.9999892	0.073428	10.17371
**194**	GO:0043011~myeloid dendritic cell differentiation	3	2.803738	0.006293	IL4, CSF2, CD86	24.7791441	0.9999892	0.073428	10.17371
**195**	GO:0043536~positive regulation of blood vessel endothelial cell migration	3	2.803738	0.006293	AKT1, MAPK14, VEGFA	24.7791441	0.9999892	0.073428	10.17371
**196**	GO:0042127~regulation of cell proliferation	6	5.607477	0.00636	XIAP, JUN, PTGS1, NFKBIA, NOS2, PLAU	5.08977014	0.9999905	0.073707	10.27622
**197**	GO:0042102~positive regulation of T cell proliferation	4	3.738318	0.006446	IL4, IL6, CD40LG, IL1B	10.4623053	0.9999918	0.074192	10.40758
**198**	GO:0044267~cellular protein metabolic process	5	4.672897	0.006639	INS, BACE1, F2, MMP2, MMP1	6.64977032	0.9999943	0.075862	10.70394
**199**	GO:0001701~in utero embryonic development	6	5.607477	0.006649	AR, RXRA, C5, VEGFA, NOS3, BCL2L1	5.0353341	0.9999944	0.075498	10.71931
**200**	GO:0006950~response to stress	4	3.738318	0.006748	MAPK1, HSP90AA1, C5, MAPK8	10.2907921	0.9999953	0.076106	10.87032
**201**	GO:0030101~natural killer cell activation	3	2.803738	0.006964	CASP8, ITGB2, IL2	23.5401869	0.9999968	0.077964	11.19841
**202**	GO:0071375~cellular response to peptide hormone stimulus	3	2.803738	0.006964	RELA, MDM2, POR	23.5401869	0.9999968	0.077964	11.19841
**203**	GO:0006968~cellular defense response	4	3.738318	0.007059	IL4, C5AR1, RELA, CCR3	10.1248116	0.9999973	0.078511	11.34347
**204**	GO:0009611~response to	4	3.738318	0.007379	ACHE, CCL2, CYP1A1, F2	9.96410028	0.9999985	0.08143	11.82691
**205**	GO:0032094~response to food	3	2.803738	0.007665	OPRM1, AKT1, CYP1A1	22.4192256	0.9999991	0.083959	12.25857

**206**	GO:0045861~negative regulation of proteolysis	3	2.803738	0.007665	AKT1, INS, F2	22.4192256	0.9999991	0.083959	12.25857
**207**	GO:1900182~positive regulation of protein localization to nucleus	3	2.803738	0.007665	AKT1, INS, F2	22.4192256	0.9999991	0.083959	12.25857
**208**	GO:0035902~response to immobilization stress	3	2.803738	0.007665	FOS, CYP1A1, PPARG	22.4192256	0.9999991	0.083959	12.25857
**209**	GO:0006921~cellular component disassembly involved in execution phase of apoptosis	3	2.803738	0.007665	CASP3, CASP8, PRKCD	22.4192256	0.9999991	0.083959	12.25857
**210**	GO:0045987~positive regulation of smooth muscle contraction	3	2.803738	0.007665	PTGS2, ADRA1B, TBXA2R	22.4192256	0.9999991	0.083959	12.25857
**211**	GO:0007166~cell surface receptor signaling pathway	7	6.542056	0.00783	ADRB2, CCL2, MAPK14, C5, CASP8, CD247, F2	4.00927758	0.9999993	0.08518	12.5061
**212**	GO:0014068~positive regulation of phosphatidylinositol 3-kinase signaling	4	3.738318	0.008043	PTPN6, INS, F2, CAT	9.65751258	0.9999996	0.08688	12.82426
**213**	GO:0051384~response to glucocorticoid	4	3.738318	0.008043	CASP3, IL6, PTGS2, BCL2	9.65751258	0.9999996	0.08688	12.82426
**214**	GO:0071345~cellular response to cytokine stimulus	3	2.803738	0.008397	CD86, NFKBIA, IL13	21.4001699	0.9999998	0.090012	13.35213
**215**	GO:0007155~cell adhesion	9	8.411215	0.008576	CCL11, ICAM1, ACHE, CCL2, CCR3, PECAM1, ITGB2, SELE, IL2	3.07714862	0.9999998	0.091308	13.61719
**216**	GO:0019221~cytokine-mediated signaling pathway	5	4.672897	0.009527	IL6, CCL2, RELA, IL1B, STAT3	5.98986944	1	0.100369	15.01486
**217**	GO:0098869~cellular oxidant detoxification	4	3.738318	0.009854	PTGS2, PTGS1, CAT, GSTP1	8.96769025	1	0.103045	15.4896
**218**	GO:0045742~positive regulation of epidermal growth factor receptor signaling pathway	3	2.803738	0.009951	AKT1, MMP9, FASLG	19.6168224	1	0.103423	15.63119
**219**	GO:0070371~ERK1 and ERK2 cascade	3	2.803738	0.009951	CCL11, MAPK1, EGF	19.6168224	1	0.103423	15.63119
**220**	GO:0097190~apoptotic signaling pathway	4	3.738318	0.010242	CASP3, BAX, CASP8, FASLG	8.84138476	1	0.105689	16.05102
**221**	GO:0060333~interferon-gamma-mediated signaling pathway	4	3.738318	0.010242	ICAM1, STAT1, PRKCD, IFNGR1	8.84138476	1	0.105689	16.05102
**222**	GO:0070098~chemokine-mediated signaling pathway	4	3.738318	0.010242	CCL11, CCL3, CCL2, CCR3	8.84138476	1	0.105689	16.05102
**223**	GO:0007569~cell aging	3	2.803738	0.010773	ICAM1, BCL2, SOD1	18.8321495	1	0.110255	16.81257
**224**	GO:0050729~positive regulation of inflammatory response	4	3.738318	0.011045	CCL11, CCL3, CCL2, IL2	8.59915504	1	0.112265	17.20093
**225**	GO:0007186~G-protein coupled receptor signaling pathway	13	12.14953	0.011105	PTPN6, CCL3, CCL2, PTGER3, C3, C5, PPARG, AKT1, AKR1C3, CCL11, INS, TBXA2R, ADRA1B	2.26935432	1	0.112218	17.28635
**226**	GO:0045840~positive regulation of mitotic nuclear division	3	2.803738	0.011623	INS, IL1B, EGF	18.1078361	1	0.116515	18.01913
**227**	GO:0002053~positive regulation of mesenchymal cell proliferation	3	2.803738	0.011623	VEGFA, TP63, STAT1	18.1078361	1	0.116515	18.01913
**228**	GO:0007202~activation of phospholipase C activity	3	2.803738	0.011623	C5AR1, PRKCD, SELE	18.1078361	1	0.116515	18.01913
**229**	GO:0051281~positive regulation of release of sequestered calcium ion into cytosol	3	2.803738	0.012501	BAX, F2, IL13	17.4371755	1	0.124123	19.24885
**230**	GO:0035987~endodermal cell differentiation	3	2.803738	0.012501	MMP9, ITGB2, MMP2	17.4371755	1	0.124123	19.24885
**231**	GO:0010800~positive regulation of peptidyl-threonine phosphorylation	3	2.803738	0.012501	MAPK1, GSK3B, EGF	17.4371755	1	0.124123	19.24885
**232**	GO:0042104~positive regulation of activated T cell proliferation	3	2.803738	0.012501	IL4, CD86, IL2	17.4371755	1	0.124123	19.24885
**233**	GO:0006808~regulation of nitrogen utilization	2	1.869159	0.012586	BAX, BCL2	156.934579	1	0.124235	19.36596
**234**	GO:0001912~positive regulation of leukocyte mediated cytotoxicity	2	1.869159	0.012586	CCL2, NOS2	156.934579	1	0.124235	19.36596
**235**	GO:0014806~smooth muscle hyperplasia	2	1.869159	0.012586	HMOX1, NOS3	156.934579	1	0.124235	19.36596
**236**	GO:0046666~retinal cell programmed cell death	2	1.869159	0.012586	BAX, FASLG	156.934579	1	0.124235	19.36596
**237**	GO:0022898~regulation of transmembrane transporter activity	2	1.869159	0.012586	INS, BCL2	156.934579	1	0.124235	19.36596
**238**	GO:0032227~negative regulation of synaptic transmission, dopaminergic	2	1.869159	0.012586	PTGS2, SLC6A4	156.934579	1	0.124235	19.36596
**239**	GO:0048247~lymphocyte chemotaxis	3	2.803738	0.013408	CCL11, CCL3, CCL2	16.8144192	1	0.131139	20.49973
**240**	GO:0000060~protein import into nucleus, translocation	3	2.803738	0.013408	AKR1C3, AKT1, NFKBIA	16.8144192	1	0.131139	20.49973
**241**	GO:0010033~response to organic substance	3	2.803738	0.013408	RELA, CREB1, SOD1	16.8144192	1	0.131139	20.49973
**242**	GO:2000352~negative regulation of endothelial cell apoptotic process	3	2.803738	0.013408	IL4, ICAM1, IL13	16.8144192	1	0.131139	20.49973
**243**	GO:0043392~negative regulation of DNA binding	3	2.803738	0.013408	JUN, HMOX1, NFKBIA	16.8144192	1	0.131139	20.49973
**244**	GO:0046718~viral entry into host cell	4	3.738318	0.014136	ICAM1, CD86, CD80, DPP4	7.84672897	1	0.137061	21.49075
**245**	GO:0097421~liver regeneration	3	2.803738	0.014342	CCND1, HMOX1, NFKBIA	16.2346117	1	0.138199	21.76979
**246**	GO:0050852~T cell receptor signaling pathway	5	4.672897	0.014392	MAPK1, RELA, CD247, IKBKG, NFKBIA	5.3018439	1	0.137919	21.83669
**247**	GO:0046326~positive regulation of glucose import	3	2.803738	0.015304	AKT1, INS, MAPK14	15.6934579	1	0.145294	23.05709
**248**	GO:0007281~germ cell development	3	2.803738	0.015304	AKT1, BAX, BCL2L1	15.6934579	1	0.145294	23.05709

249	GO:0030449~regulation of complement activation	3	2.803738	0.015304	C5AR1, C3, C5	15.6934579	1	0.145294	23.05709
250	GO:0000122~negative regulation of transcription from RNA polymerase II promoter	11	10.28037	0.015557	CCND1, RELA, RXRA, VEGFA, PPARG, ESR1, TP63, MDM2, FASLG, STAT1, STAT3	2.39761163	1	0.146761	23.39143
251	GO:0006367~transcription initiation from RNA polymerase II	5	4.672897	0.015728	AR, NR1I2, RXRA, PPARG, ESR1	5.16232169	1	0.147503	23.61773
252	GO:0051897~positive regulation of protein kinase B signaling	4	3.738318	0.016101	AKR1C3, IL6, CCL3, INS	7.47307521	1	0.149981	24.10796
253	GO:0006928~movement of cell or subcellular component	4	3.738318	0.017138	PTGS2, MAPK14, IL13, STAT3	7.29928276	1	0.158108	25.45607
254	GO:0007259~JAK-STAT cascade	3	2.803738	0.017309	CCL2, STAT1, STAT3	14.7126168	1	0.158764	25.67579
255	GO:0001974~blood vessel remodeling	3	2.803738	0.017309	BAX, MDM2, NOS3	14.7126168	1	0.158764	25.67579
256	GO:0050873~brown fat cell differentiation	3	2.803738	0.017309	ADRB2, ADRB1, PTGS2	14.7126168	1	0.158764	25.67579
257	GO:0042100~B cell proliferation	3	2.803738	0.017309	CD40LG, BCL2, PRKCD	14.7126168	1	0.158764	25.67579
258	GO:0042177~negative regulation of protein catabolic process	3	2.803738	0.017309	INS, RELA, NOS2	14.7126168	1	0.158764	25.67579
259	GO:0070059~intrinsic apoptotic signaling pathway in response to endoplasmic reticulum stress	3	2.803738	0.01835	BAX, BCL2, GSK3B	14.2667799	1	0.166722	27.00348
260	GO:2001244~positive regulation of intrinsic apoptotic signaling pathway	3	2.803738	0.01835	BAX, BCL2, BCL2L1	14.2667799	1	0.166722	27.00348
261	GO:0007565~female pregnancy	4	3.738318	0.018762	IL4, FOS, BCL2, IDO1	7.05323953	1	0.169322	27.5223
262	GO:0033861~negative regulation of NAD(P)H oxidase activity	2	1.869159	0.018819	INS, IL13	104.623053	1	0.168966	27.59419
263	GO:0010829~negative regulation of glucose transport	2	1.869159	0.018819	IL1B, PRKCB	104.623053	1	0.168966	27.59419
264	GO:0051712~positive regulation of killing of cells of other organism	2	1.869159	0.018819	FCER2, NOS2	104.623053	1	0.168966	27.59419
265	GO:0002693~positive regulation of cellular extravasation	2	1.869159	0.018819	ICAM1, CCL2	104.623053	1	0.168966	27.59419
266	GO:1903660~negative regulation of complement-dependent cytotoxicity	2	1.869159	0.018819	IL4, IL13	104.623053	1	0.168966	27.59419
267	GO:0031281~positive regulation of cyclase activity	2	1.869159	0.018819	MAPK14, MAPK8	104.623053	1	0.168966	27.59419
268	GO:0070141~response to UV-A	2	1.869159	0.018819	AKT1, CCND1	104.623053	1	0.168966	27.59419
269	GO:0002025~vasodilation by norepinephrine-epinephrine involved in regulation of systemic arterial blood pressure	2	1.869159	0.018819	ADRB2, ADRB1	104.623053	1	0.168966	27.59419
270	GO:0031649~heat generation	2	1.869159	0.018819	ADRB2, ADRB1	104.623053	1	0.168966	27.59419
271	GO:0007179~transforming growth factor beta receptor signaling pathway	4	3.738318	0.020469	FOS, CCL2, JUN, CREB1	6.82324258	1	0.181597	29.63551
272	GO:0001890~placenta development	3	2.803738	0.020511	MAPK14, PPARG, SOD1	13.4515354	1	0.181061	29.68653
273	GO:0051607~defense response to virus	5	4.672897	0.020598	IL6, CD86, RELA, BCL2, STAT1	4.75559332	1	0.180898	29.79334
274	GO:0006874~cellular calcium ion homeostasis	4	3.738318	0.021056	CCL11, CCL3, CCL2, PRKCB	6.74987438	1	0.183686	30.34918
275	GO:0008584~male gonad development	4	3.738318	0.021653	AKR1C3, BCL2, ESR1, BCL2L1	6.67806721	1	0.187531	31.06706
276	GO:0045597~positive regulation of cell differentiation	3	2.803738	0.022772	AR, INS, JUN	12.7244254	1	0.19539	32.39502
277	GO:0007188~adenylate cyclase-modulating G-protein coupled receptor signaling pathway	3	2.803738	0.022772	ADRB2, CCR3, ADRA1B	12.7244254	1	0.19539	32.39502
278	GO:0008625~extrinsic apoptotic signaling pathway via death domain receptors	3	2.803738	0.02394	BAX, BCL2, FASLG	12.3895721	1	0.203474	33.75468
279	GO:0030522~intracellular receptor signaling pathway	3	2.803738	0.02394	AR, NR1I2, STAT3	12.3895721	1	0.203474	33.75468
280	GO:0042517~positive regulation of tyrosine phosphorylation of Stat3 protein	3	2.803738	0.02394	IL6, VEGFA, STAT3	12.3895721	1	0.203474	33.75468
281	GO:0038128~ERBB2 signaling pathway	3	2.803738	0.02394	AKT1, HSP90AA1, EGF	12.3895721	1	0.203474	33.75468
282	GO:0032079~positive regulation of endodeoxyribonuclease activity	2	1.869159	0.025014	AKT1, PRKCD	78.4672897	1	0.210701	34.98319
283	GO:0010574~regulation of vascular endothelial growth factor	2	1.869159	0.025014	IL6, CCL2	78.4672897	1	0.210701	34.98319
284	GO:0090400~stress-induced premature senescence	2	1.869159	0.025014	CDKN1A, MAPK14	78.4672897	1	0.210701	34.98319
285	GO:0010760~negative regulation of macrophage chemotaxis	2	1.869159	0.025014	C5, CYP19A1	78.4672897	1	0.210701	34.98319
286	GO:0070988~demethylation	2	1.869159	0.025014	CYP1A1, POR	78.4672897	1	0.210701	34.98319
287	GO:0060574~intestinal epithelial cell maturation	2	1.869159	0.025014	CDKN1A, HIF1A	78.4672897	1	0.210701	34.98319
288	GO:0010155~regulation of proton transport	2	1.869159	0.025014	IL4, IL13	78.4672897	1	0.210701	34.98319
289	GO:1900015~regulation of cytokine production involved in inflammatory response	2	1.869159	0.025014	MAPK14, NOS2	78.4672897	1	0.210701	34.98319
290	GO:0002677~negative regulation of chronic inflammatory response	2	1.869159	0.025014	IL4, CYP19A1	78.4672897	1	0.210701	34.98319

**291**	GO:0061419~positive regulation of transcription from RNA polymerase II promoter in response to hypoxia	2	1.869159	0.025014	HIF1A, VEGFA	78.4672897	1	0.210701	34.98319
**292**	GO:0000320~re-entry into mitotic cell cycle	2	1.869159	0.025014	CCND1, GSK3B	78.4672897	1	0.210701	34.98319
**293**	GO:1900127~positive regulation of hyaluronan biosynthetic process	2	1.869159	0.025014	HAS2, EGF	78.4672897	1	0.210701	34.98319
**294**	GO:0045986~negative regulation of smooth muscle contraction	2	1.869159	0.025014	ADRB2, PTGS2	78.4672897	1	0.210701	34.98319
**295**	GO:0032570~response to progesterone	3	2.803738	0.025131	FOS, CCL2, RELA	12.0718907	1	0.210625	35.11585
**296**	GO:0000302~response to reactive oxygen species	3	2.803738	0.025131	CAT, SOD1, GSTP1	12.0718907	1	0.210625	35.11585
**297**	GO:0002576~platelet degranulation	4	3.738318	0.027437	PECAM1, VEGFA, SOD1, EGF	6.09454677	1	0.226782	37.67469
**298**	GO:0001649~osteoblast differentiation	4	3.738318	0.028125	AKT1, CCL3, FASN, CAT	6.03594536	1	0.230799	38.42041
**299**	GO:0002223~stimulatory C-type lectin receptor signaling pathway	4	3.738318	0.028823	RELA, IKBKG, NFKBIA, PRKCD	5.97846017	1	0.234825	39.16749
**300**	GO:0097191~extrinsic apoptotic signaling pathway	3	2.803738	0.028848	BAX, CASP8, FASLG	11.2096128	1	0.233972	39.19362
**301**	GO:0048015~phosphatidylinositol-mediated signaling	4	3.738318	0.02953	AKT1, CD86, CD80, EGF	5.9220596	1	0.237819	39.91567
**302**	GO:0048538~thymus development	3	2.803738	0.030132	MAPK1, BCL2, SOD1	10.9489241	1	0.241048	40.54629
**303**	GO:0014823~response to activity	3	2.803738	0.030132	CCL2, CREB1, CAT	10.9489241	1	0.241048	40.54629
**304**	GO:0033591~response to L-ascorbic acid	2	1.869159	0.03117	CAT, GSTP1	62.7738318	1	0.247277	41.61852
**305**	GO:0032611~interleukin-1 beta production	2	1.869159	0.03117	IL1B, CASP1	62.7738318	1	0.247277	41.61852
**306**	GO:0099565~chemical synaptic transmission, postsynaptic	2	1.869159	0.03117	AKT1, GSK3B	62.7738318	1	0.247277	41.61852
**307**	GO:0070431~nucleotide-binding oligomerization domain containing 2 signaling pathway	2	1.869159	0.03117	RELA, NFKBIA	62.7738318	1	0.247277	41.61852
**308**	GO:0006879~cellular iron ion homeostasis	3	2.803738	0.031439	HIF1A, HMOX1, SOD1	10.700085	1	0.248089	41.89346
**309**	GO:0034644~cellular response to UV	3	2.803738	0.031439	PTGS2, BAX, TP63	10.700085	1	0.248089	41.89346
**310**	GO:0006979~response to oxidative stress	4	3.738318	0.032449	AKT1, PTGS2, HMOX1, PTGS1	5.70671198	1	0.253981	42.9143
**311**	GO:0071363~cellular response to growth factor stimulus	3	2.803738	0.032769	MDM2, CAT, HTR3A	10.4623053	1	0.255092	43.2339
**312**	GO:0032870~cellular response to hormone stimulus	3	2.803738	0.032769	FOS, JUN, STAT3	10.4623053	1	0.255092	43.2339
**313**	GO:0008542~visual learning	3	2.803738	0.032769	HIF1A, HMGCR, CREB1	10.4623053	1	0.255092	43.2339
**314**	GO:0003151~outflow tract morphogenesis	3	2.803738	0.03412	HIF1A, JUN, VEGFA	10.2348639	1	0.263141	44.56644
**315**	GO:0051781~positive regulation of cell division	3	2.803738	0.035492	VEGFA, IL1B, CAT	10.0171008	1	0.271182	45.88995
**316**	GO:0045600~positive regulation of fat cell differentiation	3	2.803738	0.035492	AKT1, CREB1, PPARG	10.0171008	1	0.271182	45.88995
**317**	GO:0032494~response to peptidoglycan	2	1.869159	0.037288	IL6, C5AR1	52.3115265	1	0.281828	47.57702
**318**	GO:0038084~vascular endothelial growth factor signaling pathway	2	1.869159	0.037288	FLT4, VEGFA	52.3115265	1	0.281828	47.57702
**319**	GO:0010742~macrophage derived foam cell differentiation	2	1.869159	0.037288	PPARG, STAT1	52.3115265	1	0.281828	47.57702
**320**	GO:0043619~regulation of transcription from RNA polymerase II promoter in response to oxidative stress	2	1.869159	0.037288	HIF1A, HMOX1	52.3115265	1	0.281828	47.57702
**321**	GO:0032025~response to cobalt ion	2	1.869159	0.037288	CASP3, CASP8	52.3115265	1	0.281828	47.57702
**322**	GO:0043615~astrocyte cell migration	2	1.869159	0.037288	CCL3, CCL2	52.3115265	1	0.281828	47.57702
**323**	GO:0060137~maternal process involved in parturition	2	1.869159	0.037288	CCL2, CYP1A1	52.3115265	1	0.281828	47.57702
**324**	GO:0045908~negative regulation of vasodilation	2	1.869159	0.037288	HMGCR, INS	52.3115265	1	0.281828	47.57702
**325**	GO:0002933~lipid hydroxylation	2	1.869159	0.037288	CYP3A4, CYP1A1	52.3115265	1	0.281828	47.57702
**326**	GO:0042752~regulation of circadian rhythm	3	2.803738	0.0383	CREB1, PPARG, MAPK8	9.60823956	1	0.287218	48.50566
**327**	GO:0014070~response to organic cyclic compound	3	2.803738	0.0383	IL4, ICAM1, CYP1A1	9.60823956	1	0.287218	48.50566
**328**	GO:0033209~tumor necrosis factor-mediated signaling pathway	4	3.738318	0.038722	CD40LG, TNFSF15, FASLG, STAT1	5.31981625	1	0.28877	48.88877
**329**	GO:0007189~adenylate cyclase-activating G-protein coupled receptor signaling pathway	3	2.803738	0.039735	ADRB2, PTGER3, TBXA2R	9.41607477	1	0.294038	49.7959
**330**	GO:0006521~regulation of cellular amino acid metabolic process	3	2.803738	0.04119	ODC1, INS, NQO1	9.23144585	1	0.301983	51.07318
**331**	GO:0071277~cellular response to calcium ion	3	2.803738	0.04119	AKR1C3, FOS, JUN	9.23144585	1	0.301983	51.07318
**332**	GO:0019233~sensory perception of pain	3	2.803738	0.042665	OPRM1, MAPK1, PTGS2	9.05391804	1	0.309901	52.33665
**333**	GO:0032287~peripheral nervous system myelin maintenance	2	1.869159	0.043367	AKT1, SOD1	44.8384513	1	0.312988	52.92769
**334**	GO:0090050~positive regulation of cell migration involved in sprouting angiogenesis	2	1.869159	0.043367	PTGS2, VEGFA	44.8384513	1	0.312988	52.92769

**335**	GO:0030213~hyaluronan biosynthetic process	2	1.869159	0.043367	IL1B, HAS2	44.8384513	1	0.312988	52.92769
**336**	GO:0051549~positive regulation of keratinocyte migration	2	1.869159	0.043367	MMP9, HAS2	44.8384513	1	0.312988	52.92769
**337**	GO:0034351~negative regulation of glial cell apoptotic process	2	1.869159	0.043367	CCL2, PRKCD	44.8384513	1	0.312988	52.92769
**338**	GO:0060687~regulation of branching involved in prostate gland morphogenesis	2	1.869159	0.043367	RXRA, ESR1	44.8384513	1	0.312988	52.92769
**339**	GO:2000427~positive regulation of apoptotic cell clearance	2	1.869159	0.043367	CCL2, C3	44.8384513	1	0.312988	52.92769
**340**	GO:0043496~regulation of protein homodimerization activity	2	1.869159	0.043367	BAX, BCL2	44.8384513	1	0.312988	52.92769
**341**	GO:0035357~peroxisome proliferator activated receptor signaling pathway	2	1.869159	0.043367	RXRA, PPARG	44.8384513	1	0.312988	52.92769
**342**	GO:0044321~response to leptin	2	1.869159	0.043367	CCND1, STAT3	44.8384513	1	0.312988	52.92769
**343**	GO:1901796~regulation of signal transduction by p53 class mediator	4	3.738318	0.043803	AKT1, MAPK14, TP63, MDM2	5.06240579	1	0.314426	53.29093
**344**	GO:0048146~positive regulation of fibroblast proliferation	3	2.803738	0.045673	CDKN1A, JUN, ESR1	8.71858775	1	0.324411	54.81908
**345**	GO:0038096~Fc-gamma receptor signaling pathway involved in phagocytosis	4	3.738318	0.046463	MAPK1, HSP90AA1, CD247, PRKCD	4.9428214	1	0.327851	55.45029
**346**	GO:0042475~odontogenesis of dentin-containing tooth	3	2.803738	0.047206	BAX, TP63, CA2	8.56006797	1	0.330974	56.0366
**347**	GO:0016049~cell growth	3	2.803738	0.048757	AR, IL6, BCL2	8.40720961	1	0.338722	57.23748
**348**	GO:0097011~cellular response to granulocyte macrophage colony-stimulating factor stimulus	2	1.869159	0.049409	AKT1, MAPK1	39.2336449	1	0.341206	57.7325
**349**	GO:0032872~regulation of stress-activated MAPK cascade	2	1.869159	0.049409	MAPK1, GSTP1	39.2336449	1	0.341206	57.7325
**350**	GO:0045630~positive regulation of T-helper 2 cell differentiation	2	1.869159	0.049409	IL6, CD86	39.2336449	1	0.341206	57.7325
**351**	GO:0035234~ectopic germ cell programmed cell death	2	1.869159	0.049409	BAX, IL1B	39.2336449	1	0.341206	57.7325
**352**	GO:0010888~negative regulation of lipid storage	2	1.869159	0.049409	IL6, NFKBIA	39.2336449	1	0.341206	57.7325
**353**	GO:0043497~regulation of protein heterodimerization activity	2	1.869159	0.049409	BAX, BCL2	39.2336449	1	0.341206	57.7325
**354**	GO:1904706~negative regulation of vascular smooth muscle cell proliferation	2	1.869159	0.049409	HMOX1, GSTP1	39.2336449	1	0.341206	57.7325
**355**	GO:0010544~negative regulation of platelet activation	2	1.869159	0.049409	F2, NOS3	39.2336449	1	0.341206	57.7325
**356**	GO:0009812~flavonoid metabolic process	2	1.869159	0.049409	CYP1A1, POR	39.2336449	1	0.341206	57.7325
**357**	GO:0002438~acute inflammatory response to antigenic stimulus	2	1.869159	0.049409	OPRM1, ICAM1	39.2336449	1	0.341206	57.7325
**358**	GO:0048304~positive regulation of isotype switching to IgG isotypes	2	1.869159	0.049409	IL4, IL2	39.2336449	1	0.341206	57.7325
**359**	GO:0002262~myeloid cell homeostasis	2	1.869159	0.049409	BAX, SOD1	39.2336449	1	0.341206	57.7325
**360**	GO:0006527~arginine catabolic process	2	1.869159	0.049409	NOS3, NOS2	39.2336449	1	0.341206	57.7325
**361**	GO:0033598~mammary gland epithelial cell proliferation	2	1.869159	0.049409	MAPK1, CCND1	39.2336449	1	0.341206	57.7325
**362**	GO:0032966~negative regulation of collagen biosynthetic process	2	1.869159	0.049409	IL6, PPARG	39.2336449	1	0.341206	57.7325
**363**	GO:0090037~positive regulation of protein kinase C signaling	2	1.869159	0.049409	FLT4, VEGFA	39.2336449	1	0.341206	57.7325
**364**	GO:0060736~prostate gland growth	2	1.869159	0.049409	AR, CYP19A1	39.2336449	1	0.341206	57.7325
**365**	GO:0006959~humoral immune response	3	2.803738	0.050327	IL6, CCL2, BCL2	8.25971471	1	0.34517	58.42112
**366**	GO:0060041~retina development in camera-type eye	3	2.803738	0.051915	IL4, ACHE, BAX	8.11730583	1	0.352821	59.58699
**367**	GO:0051259~protein oligomerization	3	2.803738	0.051915	AR, BAX, SLC6A4	8.11730583	1	0.352821	59.58699
**368**	GO:0050821~protein stabilization	4	3.738318	0.054907	CDKN1A, HSP90AA1, CREB1, PRKCD	4.61572292	1	0.368002	61.70101
**369**	GO:0050679~positive regulation of epithelial cell proliferation	3	2.803738	0.055144	IL6, C5AR1, VEGFA	7.84672897	1	0.367987	61.86353
**370**	GO:0051289~protein homotetramerization	3	2.803738	0.055144	RXRA, TP63, CAT	7.84672897	1	0.367987	61.86353
**371**	GO:0008635~activation of cysteine-type endopeptidase activity involved in apoptotic process by cytochrome c	2	1.869159	0.055412	CASP3, BAX	34.874351	1	0.368143	62.04711
**372**	GO:0010838~positive regulation of keratinocyte proliferation	2	1.869159	0.055412	TP63, HAS2	34.874351	1	0.368143	62.04711
**373**	GO:0009651~response to salt stress	2	1.869159	0.055412	HSP90AA1, BAX	34.874351	1	0.368143	62.04711
**374**	GO:0050665~hydrogen peroxide biosynthetic process	2	1.869159	0.055412	CYP1A1, SOD1	34.874351	1	0.368143	62.04711
**375**	GO:0060068~vagina development	2	1.869159	0.055412	BAX, ESR1	34.874351	1	0.368143	62.04711
**376**	GO:0010469~regulation of receptor activity	2	1.869159	0.055412	PRKCD, PLAU	34.874351	1	0.368143	62.04711
**377**	GO:0048245~eosinophil chemotaxis	2	1.869159	0.055412	CCL11, CCL3	34.874351	1	0.368143	62.04711
**378**	GO:0010224~response to UV-B	2	1.869159	0.055412	RELA, BCL2	34.874351	1	0.368143	62.04711

**379**	GO:0070102~interleukin-6-mediated signaling pathway	2	1.869159	0.055412	IL6, STAT3	34.874351	1	0.368143	62.04711
**380**	GO:0070234~positive regulation of T cell apoptotic process	2	1.869159	0.055412	ICAM1, IDO1	34.874351	1	0.368143	62.04711
**381**	GO:0002024~diet induced thermogenesis	2	1.869159	0.055412	ADRB2, ADRB1	34.874351	1	0.368143	62.04711
**382**	GO:0006977~DNA damage response, signal transduction by p53 class mediator resulting in cell cycle arrest	3	2.803738	0.058442	CDKN1A, BAX, MDM2	7.59360868	1	0.382958	64.06375
**383**	GO:0007613~memory	3	2.803738	0.058442	PTGS2, CREB1, SLC6A4	7.59360868	1	0.382958	64.06375
**384**	GO:0016525~negative regulation of angiogenesis	3	2.803738	0.058442	CCL2, FASLG, STAT1	7.59360868	1	0.382958	64.06375
**385**	GO:0001893~maternal placenta development	2	1.869159	0.061378	AKT1, RXRA	31.3869159	1	0.39687	65.92151
**386**	GO:1901030~positive regulation of mitochondrial outer membrane permeabilization involved in apoptotic signaling pathway	2	1.869159	0.061378	BAX, GSK3B	31.3869159	1	0.39687	65.92151
**387**	GO:0033327~Leydig cell differentiation	2	1.869159	0.061378	AR, CCND1	31.3869159	1	0.39687	65.92151
**388**	GO:0033160~positive regulation of protein import into nucleus, translocation	2	1.869159	0.061378	IL6, HSP90AA1	31.3869159	1	0.39687	65.92151
**389**	GO:0042359~vitamin D metabolic process	2	1.869159	0.061378	CYP3A4, CYP1A1	31.3869159	1	0.39687	65.92151
**390**	GO:0051146~striated muscle cell differentiation	2	1.869159	0.061378	AKT1, MAPK14	31.3869159	1	0.39687	65.92151
**391**	GO:0032436~positive regulation of proteasomal ubiquitin-dependent protein catabolic process	3	2.803738	0.061807	AKT1, GSK3B, MDM2	7.35630841	1	0.397722	66.18505
**392**	GO:0006006~glucose metabolic process	3	2.803738	0.066974	AKT1, INS, MAPK14	7.02692147	1	0.422199	69.21484
**393**	GO:0032868~response to insulin	3	2.803738	0.066974	IL6, RELA, CAT	7.02692147	1	0.422199	69.21484
**394**	GO:0002064~epithelial cell development	2	1.869159	0.067307	ESR1, TP63	28.5335599	1	0.422442	69.4006
**395**	GO:0043306~positive regulation of mast cell degranulation	2	1.869159	0.067307	IL4, IL13	28.5335599	1	0.422442	69.4006
**396**	GO:0050732~negative regulation of peptidyl-tyrosine phosphorylation	2	1.869159	0.067307	PTPN6, PRKCD	28.5335599	1	0.422442	69.4006
**397**	GO:0031284~positive regulation of guanylate cyclase activity	2	1.869159	0.067307	NOS3, NOS2	28.5335599	1	0.422442	69.4006
**398**	GO:0031571~mitotic G1 DNA damage checkpoint	2	1.869159	0.067307	CCND1, TP63	28.5335599	1	0.422442	69.4006
**399**	GO:0010759~positive regulation of macrophage chemotaxis	2	1.869159	0.067307	CCL2, C5AR1	28.5335599	1	0.422442	69.4006
**400**	GO:0051024~positive regulation of immunoglobulin secretion	2	1.869159	0.067307	IL6, IL2	28.5335599	1	0.422442	69.4006
**401**	GO:0033197~response to vitamin E	2	1.869159	0.067307	CCND1, CAT	28.5335599	1	0.422442	69.4006
**402**	GO:0009743~response to carbohydrate	2	1.869159	0.067307	MDM2, IL1B	28.5335599	1	0.422442	69.4006
**403**	GO:0030278~regulation of ossification	2	1.869159	0.067307	MAPK1, MAPK14	28.5335599	1	0.422442	69.4006
**404**	GO:0008203~cholesterol metabolic process	3	2.803738	0.068728	IL4, RXRA, CAT	6.92358439	1	0.427953	70.18357
**405**	GO:0035690~cellular response to drug	3	2.803738	0.070497	CCL2, IL1B, NOS2	6.82324258	1	0.435026	71.13152
**406**	GO:0071300~cellular response to retinoic acid	3	2.803738	0.07228	CCL2, SLC6A4, PPARG	6.72576769	1	0.442037	72.05866
**407**	GO:0007265~Ras protein signal transduction	3	2.803738	0.07228	CDKN1A, MAPK14, JUN	6.72576769	1	0.442037	72.05866
**408**	GO:0043950~positive regulation of cAMP-mediated signaling	2	1.869159	0.073198	OPRM1, ADRB1	26.1557632	1	0.444914	72.52468
**409**	GO:0031669~cellular response to nutrient levels	2	1.869159	0.073198	ICAM1, IL6	26.1557632	1	0.444914	72.52468
**410**	GO:0040015~negative regulation of multicellular organism growth	2	1.869159	0.073198	ADRB2, ADRB1	26.1557632	1	0.444914	72.52468
**411**	GO:0097028~dendritic cell differentiation	2	1.869159	0.073198	IL4, CSF2	26.1557632	1	0.444914	72.52468
**412**	GO:0031000~response to caffeine	2	1.869159	0.073198	IL6, PPARG	26.1557632	1	0.444914	72.52468
**413**	GO:0007171~activation of transmembrane receptor protein tyrosine kinase activity	2	1.869159	0.073198	ADRB2, EGF	26.1557632	1	0.444914	72.52468
**414**	GO:0019725~cellular homeostasis	2	1.869159	0.073198	CCL2, MCL1	26.1557632	1	0.444914	72.52468
**415**	GO:0042738~exogenous drug catabolic process	2	1.869159	0.073198	CYP3A4, NR1I2	26.1557632	1	0.444914	72.52468
**416**	GO:0071391~cellular response to estrogen stimulus	2	1.869159	0.079053	ESR1, MDM2	24.1437815	1	0.470059	75.32996
**417**	GO:0060334~regulation of interferon-gamma-mediated signaling pathway	2	1.869159	0.079053	STAT1, IFNGR1	24.1437815	1	0.470059	75.32996
**418**	GO:0032026~response to magnesium ion	2	1.869159	0.079053	CCND1, MDM2	24.1437815	1	0.470059	75.32996
**419**	GO:0048246~macrophage chemotaxis	2	1.869159	0.079053	CCL3, CCL2	24.1437815	1	0.470059	75.32996
**420**	GO:0010745~negative regulation of macrophage derived foam cell differentiation	2	1.869159	0.079053	PPARG, NFKBIA	24.1437815	1	0.470059	75.32996

**421**	GO:0019372~lipoxygenase pathway	2	1.869159	0.079053	PTGS2, ALOX5	24.1437815	1	0.470059	75.32996
**422**	GO:0051770~positive regulation of nitric-oxide synthase biosynthetic process	2	1.869159	0.079053	CCL2, FCER2	24.1437815	1	0.470059	75.32996
**423**	GO:0001878~response to yeast	2	1.869159	0.079053	IL6, CD86	24.1437815	1	0.470059	75.32996
**424**	GO:0045019~negative regulation of nitric oxide biosynthetic process	2	1.869159	0.079053	OPRM1, IL4	24.1437815	1	0.470059	75.32996
**425**	GO:0043922~negative regulation by host of viral transcription	2	1.869159	0.079053	CCL3, JUN	24.1437815	1	0.470059	75.32996
**426**	GO:0046685~response to arsenic-containing substance	2	1.869159	0.079053	CDKN1A, CYP1A1	24.1437815	1	0.470059	75.32996
**427**	GO:0048546~digestive tract morphogenesis	2	1.869159	0.079053	HIF1A, BCL2	24.1437815	1	0.470059	75.32996
**428**	GO:0006693~prostaglandin metabolic process	2	1.869159	0.079053	AKR1C3, PTGS2	24.1437815	1	0.470059	75.32996
**429**	GO:0006957~complement activation, alternative pathway	2	1.869159	0.079053	C3, C5	24.1437815	1	0.470059	75.32996
**430**	GO:0030334~regulation of cell migration	3	2.803738	0.079558	AKT1, MMP10, PECAM1	6.36221268	1	0.470867	75.55914
**431**	GO:1900034~regulation of cellular response to heat	3	2.803738	0.081412	MAPK1, HSP90AA1, GSK3B	6.27738318	1	0.477561	76.3825
**432**	GO:0030216~keratinocyte differentiation	3	2.803738	0.08328	AKR1C3, CASP3, TP63	6.19478603	1	0.484188	77.18537
**433**	GO:0031103~axon regeneration	2	1.869159	0.084871	JUN, BCL2	22.4192256	1	0.489524	77.84896
**434**	GO:0001516~prostaglandin biosynthetic process	2	1.869159	0.084871	PTGS2, PTGS1	22.4192256	1	0.489524	77.84896
**435**	GO:0045721~negative regulation of gluconeogenesis	2	1.869159	0.084871	IL6, INS	22.4192256	1	0.489524	77.84896
**436**	GO:0010243~response to organonitrogen compound	2	1.869159	0.084871	CDKN1A, CCND1	22.4192256	1	0.489524	77.84896
**437**	GO:0071354~cellular response to interleukin-6	2	1.869159	0.084871	CCL2, RELA	22.4192256	1	0.489524	77.84896
**438**	GO:1902176~negative regulation of oxidative stress-induced intrinsic apoptotic signaling pathway	2	1.869159	0.084871	AKT1, INS	22.4192256	1	0.489524	77.84896
**439**	GO:0045821~positive regulation of glycolytic process	2	1.869159	0.084871	HIF1A, INS	22.4192256	1	0.489524	77.84896
**440**	GO:0042176~regulation of protein catabolic process	2	1.869159	0.084871	ODC1, MDM2	22.4192256	1	0.489524	77.84896
**441**	GO:0030194~positive regulation of blood coagulation	2	1.869159	0.084871	F2, TBXA2R	22.4192256	1	0.489524	77.84896
**442**	GO:0045454~cell redox homeostasis	3	2.803738	0.085161	IL6, NOS3, NOS2	6.11433426	1	0.489314	77.96787
**443**	GO:0046688~response to copper ion	2	1.869159	0.090652	ICAM1, SOD1	20.9246106	1	0.510555	80.11088
**444**	GO:0030502~negative regulation of bone mineralization	2	1.869159	0.090652	CCL3, HIF1A	20.9246106	1	0.510555	80.11088
**445**	GO:0032469~endoplasmic reticulum calcium ion homeostasis	2	1.869159	0.090652	BAX, BCL2	20.9246106	1	0.510555	80.11088
**446**	GO:0060397~JAK-STAT cascade involved in growth hormone signaling pathway	2	1.869159	0.090652	MAPK1, STAT3	20.9246106	1	0.510555	80.11088
**447**	GO:0045725~positive regulation of glycogen biosynthetic process	2	1.869159	0.090652	AKT1, INS	20.9246106	1	0.510555	80.11088
**448**	GO:0006691~leukotriene metabolic process	2	1.869159	0.090652	LTA4H, ALOX5	20.9246106	1	0.510555	80.11088
**449**	GO:0008631~intrinsic apoptotic signaling pathway in response to oxidative stress	2	1.869159	0.090652	BCL2, PRKCD	20.9246106	1	0.510555	80.11088
**450**	GO:0042953~lipoprotein transport	2	1.869159	0.090652	PPARG, PRKCB	20.9246106	1	0.510555	80.11088
**451**	GO:0045892~negative regulation of transcription, DNA-templated	7	6.542056	0.096026	IL4, NR1I2, RELA, JUN, PPARG, TP63, MDM2	2.20148709	1	0.530415	82.01658
**452**	GO:0090280~positive regulation of calcium ion import	2	1.869159	0.096398	CCL3, CCL2	19.6168224	1	0.530396	82.14194
**453**	GO:0050995~negative regulation of lipid catabolic process	2	1.869159	0.096398	INS, IL1B	19.6168224	1	0.530396	82.14194
**454**	GO:0006978~DNA damage response, signal transduction by p53 class mediator resulting in transcription of p21 class mediator	2	1.869159	0.096398	CDKN1A, TP63	19.6168224	1	0.530396	82.14194
**455**	GO:2000045~regulation of G1/S transition of mitotic cell cycle	2	1.869159	0.096398	PTPN6, CCND1	19.6168224	1	0.530396	82.14194
**456**	GO:0042307~positive regulation of protein import into nucleus	2	1.869159	0.096398	MAPK14, PRKCD	19.6168224	1	0.530396	82.14194
**457**	GO:2001234~negative regulation of apoptotic signaling pathway	2	1.869159	0.096398	BAX, BCL2	19.6168224	1	0.530396	82.14194
**458**	GO:0070848~response to growth factor	2	1.869159	0.096398	OPRM1, FASLG	19.6168224	1	0.530396	82.14194
**459**	GO:0060070~canonical Wnt signaling pathway	3	2.803738	0.096707	CCND1, GSK3B, EGF	5.6723342	1	0.530134	82.24543

AD: atopic dermatitis. OB: oral bioavailability; DL: drug-likeness

Table S6. The enriched KEGG pathways for intersection targets between compound (OB ⩾ 30% and DL ⩾ 0.18) and AD related targets

	ID	Description	GeneRati	BgRatio	pvalue	p.adjust	qvalue	geneID	Count
**1**	hsa04933	AGE-RAGE signaling pathway in diabetic complications	27/109	100/7229	1.74E-27	3.07E-25	1.12E-25	RELA/BCL2/BAX/CASP3/AKT1/MAPK3/MAPK1/PRKCA/IL1B/PRKCD/VEGFA/CCND1/MMP2/JUN/IL6/ICAM1/MAPK14/CCL2/MAPK8/STAT1/SELE/VCAM1/PRKCB/NOS3/THBD/COL1A1/IL1A	27
**2**	hsa04668	TNF signaling pathway	24/109	112/7229	8.04E-22	7.12E-20	2.58E-20	PTGS2/RELA/CASP3/CASP8/AKT1/MAPK3/MAPK1/IL1B/MMP9/JUN/IL6/NFKBIA/CASP7/ICAM1/MAPK14/CCL2/MAPK8/SELE/VCAM1/MMP3/FOS/CXCL2/CXCL10/IRF1	24
**3**	hsa04657	IL-17 signaling pathway	22/109	94/7229	6.08E-21	3.59E-19	1.30E-19	PTGS2/HSP90AA1/RELA/CASP3/CASP8/MAPK3/MAPK1/IL1B/MMP9/JUN/IL6/NFKBIA/MMP1/IL4/MAPK14/GSK3B/CCL2/MAPK8/MMP3/FOS/CXCL2/CXCL10	22
**4**	hsa01524	Platinum drug resistance	16/109	73/7229	5.99E-15	2.65E-13	9.61E-14	BCL2/CDKN1A/BAX/CASP3/CASP8/FASLG/AKT1/MAPK3/MAPK1/GSTP1/BCL2L1/MDM2/ERBB2/TOP2A/XIAP/GSTM1	16
**5**	hsa04659	Th17 cell differentiation	18/109	107/7229	1.49E-14	5.26E-13	1.91E-13	HSP90AA1/RELA/MAPK3/MAPK1/IL1B/RXRA/JUN/IL6/NFKBIA/IL2/IFNGR1/IL4/MAPK14/MAPK8/STAT1/AHR/FOS/HIF1A	18
**6**	hsa04066	HIF-1 signaling pathway	18/109	109/7229	2.09E-14	6.15E-13	2.23E-13	NOS2/RELA/BCL2/CDKN1A/AKT1/MAPK3/MAPK1/PRKCA/HMOX1/EGFR/VEGFA/IL6/ERBB2/IFNGR1/EGF/HIF1A/PRKCB/NOS3	18
**7**	hsa01522	Endocrine resistance	17/109	98/7229	5.02E-14	1.27E-12	4.60E-13	BCL2/CDKN1A/BAX/ESR1/AKT1/MAPK3/MAPK1/EGFR/CCND1/MMP2/MMP9/JUN/MDM2/ERBB2/MAPK14/MAPK8/FOS	17
**8**	hsa04625	C-type lectin receptor signaling pathway	17/109	104/7229	1.40E-13	3.10E-12	1.13E-12	PTGS2/RELA/CASP8/AKT1/MAPK3/MAPK1/IL1B/PRKCD/JUN/IL6/NFKBIA/MDM2/IL2/MAPK14/MAPK8/STAT1/IRF1	17
**9**	hsa04926	Relaxin signaling pathway	18/109	129/7229	4.32E-13	8.50E-12	3.08E-12	NOS2/RELA/AKT1/MAPK3/MAPK1/PRKCA/EGFR/VEGFA/MMP2/MMP9/JUN/NFKBIA/MMP1/MAPK14/MAPK8/FOS/NOS3/COL1A1	18
**10**	hsa04210	Apoptosis	18/109	136/7229	1.10E-12	1.94E-11	7.05E-12	RELA/BCL2/BAX/CASP3/CASP8/FASLG/AKT1/MAPK3/MAPK1/BCL2L1/JUN/NFKBIA/CASP7/MCL1/XIAP/MAPK8/FOS/CTSD	18
**11**	hsa04620	Toll-like receptor signaling pathway	16/109	104/7229	2.06E-12	3.31E-11	1.20E-11	RELA/CASP8/AKT1/MAPK3/MAPK1/IL1B/JUN/IL6/NFKBIA/MAPK14/MAPK8/STAT1/FOS/CXCL11/CXCL10/SPP1	16
**12**	hsa05133	Pertussis	14/109	76/7229	4.24E-12	6.25E-11	2.27E-11	NOS2/RELA/CASP3/MAPK3/MAPK1/IL1B/JUN/IL6/CASP7/MAPK14/MAPK8/FOS/IL1A/IRF1	14
**13**	hsa01521	EGFR tyrosine kinase inhibitor resistance	14/109	79/7229	7.39E-12	9.72E-11	3.53E-11	BCL2/BAX/AKT1/MAPK3/MAPK1/PRKCA/EGFR/VEGFA/BCL2L1/IL6/ERBB2/GSK3B/EGF/PRKCB	14
**14**	hsa04151	PI3K-Akt signaling pathway	26/109	354/7229	7.69E-12	9.72E-11	3.53E-11	HSP90AA1/RELA/BCL2/CDKN1A/FASLG/AKT1/MAPK3/MAPK1/PRKCA/RXRA/EGFR/VEGFA/CCND1/BCL2L1/IL6/MDM2/ERBB2/MCL1/IL2/IL4/GSK3B/EGF/NOS3/COL1A1/SPP1/IGF2	26
**15**	hsa05132	Salmonella infection	13/109	80/7229	1.37E-10	1.61E-09	5.85E-10	NOS2/RELA/MAPK3/MAPK1/IL1B/JUN/IL6/IFNGR1/MAPK14/MAPK8/FOS/IL1A/CXCL2	13
**16**	hsa04621	NOD-like receptor signaling pathway	18/109	181/7229	1.47E-10	1.63E-09	5.92E-10	HSP90AA1/RELA/BCL2/CASP8/MAPK3/MAPK1/IL1B/PRKCD/BCL2L1/JUN/IL6/NFKBIA/XIAP/MAPK14/CCL2/MAPK8/STAT1/CXCL2	18
**17**	hsa04917	Prolactin signaling pathway	12/109	70/7229	3.83E-10	3.99E-09	1.45E-09	RELA/ESR1/AKT1/MAPK3/MAPK1/CCND1/MAPK14/GSK3B/MAPK8/STAT1/FOS/IRF1	12
**18**	hsa04915	Estrogen signaling pathway	15/109	138/7229	1.67E-09	1.64E-08	5.96E-09	HSP90AA1/BCL2/ESR1/AKT1/MAPK3/MAPK1/PRKCD/EGFR/MMP2/MMP9/JUN/OPRM1/FOS/NOS3/CTSD	15
**19**	hsa04010	MAPK signaling pathway	21/109	295/7229	1.95E-09	1.81E-08	6.58E-09	RELA/CASP3/FASLG/AKT1/MAPK3/MAPK1/PRKCA/IL1B/EGFR/VEGFA/JUN/ERBB2/MAPK14/MAPK8/FOS/EGF/PRKCB/HSPB1/IL1A/IGF2/RASA1	21
**20**	hsa04064	NF-kappa B signaling pathway	13/109	102/7229	3.08E-09	2.73E-08	9.90E-09	PTGS2/RELA/BCL2/IL1B/BCL2L1/NFKBIA/ICAM1/XIAP/CD40LG/VCAM1/PLAU/PRKCB/CXCL2	13
**21**	hsa04012	ErbB signaling pathway	12/109	85/7229	3.93E-09	3.16E-08	1.15E-08	CDKN1A/AKT1/MAPK3/MAPK1/PRKCA/EGFR/JUN/ERBB2/GSK3B/MAPK8/EGF/PRKCB	12
**22**	hsa04660	T cell receptor signaling pathway	13/109	104/7229	3.93E-09	3.16E-08	1.15E-08	RELA/AKT1/MAPK3/MAPK1/JUN/NFKBIA/IL2/IL4/CD40LG/MAPK14/GSK3B/MAPK8/FOS	13
**23**	hsa04932	Non-alcoholic fatty liver disease (NAFLD)	15/109	149/7229	4.89E-09	3.76E-08	1.36E-08	RELA/BAX/CASP3/CASP8/FASLG/AKT1/PPARA/IL1B/RXRA/JUN/IL6/CASP7/GSK3B/MAPK8/IL1A	15
**24**	hsa04510	Focal adhesion	17/109	199/7229	5.33E-09	3.93E-08	1.43E-08	BCL2/AKT1/MAPK3/MAPK1/PRKCA/EGFR/VEGFA/CCND1/JUN/ERBB2/XIAP/GSK3B/MAPK8/EGF/PRKCB/COL1A1/SPP1	17
**25**	hsa04380	Osteoclast differentiation	14/109	128/7229	5.62E-09	3.98E-08	1.44E-08	RELA/AKT1/MAPK3/MAPK1/PPARG/IL1B/JUN/NFKBIA/IFNGR1/MAPK14/MAPK8/STAT1/FOS/IL1A	14
**26**	hsa04658	Th1 and Th2 cell differentiation	12/109	92/7229	9.92E-09	6.75E-08	2.45E-08	RELA/MAPK3/MAPK1/JUN/NFKBIA/IL2/IFNGR1/IL4/MAPK14/MAPK8/STAT1/FOS	12
**27**	hsa04370	VEGF signaling pathway	10/109	59/7229	1.36E-08	8.89E-08	3.23E-08	PTGS2/AKT1/MAPK3/MAPK1/PRKCA/VEGFA/MAPK14/PRKCB/NOS3/HSPB1	10
**28**	hsa04215	Apoptosis - multiple species	8/109	32/7229	1.61E-08	1.02E-07	3.68E-08	BCL2/BAX/CASP3/CASP8/BCL2L1/CASP7/XIAP/MAPK8	8
**29**	hsa04722	Neurotrophin signaling pathway	13/109	119/7229	2.08E-08	1.27E-07	4.61E-08	RELA/BCL2/BAX/FASLG/AKT1/MAPK3/MAPK1/PRKCD/JUN/NFKBIA/MAPK14/GSK3B/MAPK8	13

**30**	hsa05130	Pathogenic Escherichia coli infection	16/109	202/7229	4.62E-08	2.72E-07	9.88E-08	RELA/BAX/CASP3/CASP8/FASLG/MAPK3/MAPK1/IL1B/JUN/IL6/NFKBIA/CASP7/MAPK14/MAPK8/FOS/CLDN4	16
**31**	hsa04068	FoxO signaling pathway	13/109	131/7229	6.66E-08	3.80E-07	1.38E-07	CDKN1A/FASLG/AKT1/MAPK3/MAPK1/CAT/EGFR/CCND1/IL6/MDM2/MAPK14/MAPK8/EGF	13
**32**	hsa05131	Shigellosis	17/109	236/7229	6.86E-08	3.80E-07	1.38E-07	RELA/BCL2/BAX/AKT1/MAPK3/MAPK1/IL1B/PRKCD/EGFR/BCL2L1/JUN/NFKBIA/MDM2/MAPK14/GSK3B/MAPK8/GJA1	17
**33**	hsa04630	JAK-STAT signaling pathway	14/109	162/7229	1.17E-07	6.25E-07	2.27E-07	BCL2/CDKN1A/AKT1/EGFR/CCND1/BCL2L1/IL10RB/IL6/MCL1/IL2/IFNGR1/IL4/STAT1/EGF	14
**34**	hsa04071	Sphingolipid signaling pathway	12/109	119/7229	1.85E-07	9.35E-07	3.39E-07	RELA/BCL2/BAX/AKT1/MAPK3/MAPK1/PRKCA/MAPK14/MAPK8/PRKCB/NOS3/CTSD	12
**35**	hsa04919	Thyroid hormone signaling pathway	12/109	119/7229	1.85E-07	9.35E-07	3.39E-07	ESR1/AKT1/MAPK3/MAPK1/PRKCA/RXRA/CCND1/MDM2/GSK3B/STAT1/HIF1A/PRKCB	12
**36**	hsa05135	Yersinia infection	9/109	62/7229	3.01E-07	1.48E-06	5.37E-07	AKT1/IL1B/JUN/IL6/NFKBIA/IL2/MAPK14/GSK3B/FOS	9
**37**	hsa05020	Prion diseases	7/109	35/7229	6.93E-07	3.32E-06	1.20E-06	BAX/MAPK3/MAPK1/SOD1/IL1B/IL6/IL1A	7
**38**	hsa04726	Serotonergic synapse	11/109	115/7229	1.06E-06	4.92E-06	1.79E-06	PTGS1/PTGS2/CASP3/MAPK3/MAPK1/PRKCA/SLC6A4/ALOX5/PRKCB/HTR3A/MAOA	11
**39**	hsa04115	p53 signaling pathway	9/109	72/7229	1.11E-06	5.04E-06	1.83E-06	BCL2/CDKN1A/BAX/CASP3/CASP8/CCND1/BCL2L1/MDM2/IGFBP3	9
**40**	hsa04912	GnRH signaling pathway	10/109	93/7229	1.14E-06	5.04E-06	1.83E-06	MAPK3/MAPK1/PRKCA/PRKCD/EGFR/MMP2/JUN/MAPK14/MAPK8/PRKCB	10
**41**	hsa04935	Growth hormone synthesis, secretion and action	11/109	119/7229	1.49E-06	6.43E-06	2.33E-06	AKT1/MAPK3/MAPK1/PRKCA/MAPK14/GSK3B/MAPK8/STAT1/FOS/PRKCB/IGFBP3	11
**42**	hsa05134	Legionellosis	8/109	57/7229	1.85E-06	7.78E-06	2.82E-06	RELA/CASP3/CASP8/IL1B/IL6/NFKBIA/CASP7/CXCL2	8
**43**	hsa04662	B cell receptor signaling pathway	9/109	82/7229	3.38E-06	1.39E-05	5.05E-06	RELA/AKT1/MAPK3/MAPK1/JUN/NFKBIA/GSK3B/FOS/PRKCB	9
**44**	hsa04931	Insulin resistance	10/109	108/7229	4.52E-06	1.80E-05	6.54E-06	RELA/AKT1/PPARA/PRKCD/IL6/NFKBIA/GSK3B/MAPK8/PRKCB/NOS3	10
**45**	hsa04062	Chemokine signaling pathway	13/109	189/7229	4.58E-06	1.80E-05	6.54E-06	RELA/AKT1/MAPK3/MAPK1/PRKCD/NFKBIA/GSK3B/CCL2/STAT1/PRKCB/CXCL11/CXCL2/CXCL10	13
**46**	hsa04664	Fc epsilon RI signaling pathway	8/109	68/7229	7.20E-06	2.77E-05	1.01E-05	AKT1/MAPK3/MAPK1/PRKCA/IL4/MAPK14/MAPK8/ALOX5	8
**47**	hsa04014	Ras signaling pathway	14/109	232/7229	8.85E-06	3.31E-05	1.20E-05	RELA/FASLG/AKT1/MAPK3/MAPK1/PRKCA/EGFR/VEGFA/BCL2L1/MAPK8/EGF/PRKCB/IGF2/RASA1	14
**48**	hsa05120	Epithelial cell signaling in Helicobacter pylori infection	8/109	70/7229	8.97E-06	3.31E-05	1.20E-05	RELA/CASP3/EGFR/JUN/NFKBIA/MAPK14/MAPK8/CXCL2	8
**49**	hsa05014	Amyotrophic lateral sclerosis (ALS)	7/109	51/7229	9.79E-06	3.54E-05	1.28E-05	BCL2/BAX/CASP3/SOD1/CAT/BCL2L1/MAPK14	7
**50**	hsa04921	Oxytocin signaling pathway	11/109	153/7229	1.72E-05	6.08E-05	2.21E-05	PTGS2/CDKN1A/MAPK3/MAPK1/PRKCA/EGFR/CCND1/JUN/FOS/PRKCB/NOS3	11
**51**	hsa04218	Cellular senescence	11/109	160/7229	2.62E-05	9.08E-05	3.30E-05	RELA/CDKN1A/AKT1/MAPK3/MAPK1/CCND1/IL6/MDM2/MAPK14/IL1A/IGFBP3	11
**52**	hsa04928	Parathyroid hormone synthesis, secretion and action	9/109	106/7229	2.81E-05	9.55E-05	3.47E-05	BCL2/CDKN1A/MAPK3/MAPK1/PRKCA/RXRA/EGFR/FOS/PRKCB	9
**53**	hsa04217	Necroptosis	11/109	162/7229	2.94E-05	9.82E-05	3.56E-05	HSP90AA1/BCL2/BAX/CASP8/FASLG/IL1B/IFNGR1/XIAP/MAPK8/STAT1/IL1A	11
**54**	hsa04540	Gap junction	8/109	88/7229	4.88E-05	0.00016	5.80E-05	MAPK3/MAPK1/PRKCA/EGFR/EGF/GJA1/PRKCB/ADRB1	8
**55**	hsa04024	cAMP signaling pathway	12/109	216/7229	9.09E-05	0.000292	0.000106	ADRB2/RELA/AKT1/MAPK3/MAPK1/PPARA/JUN/NFKBIA/PTGER3/MAPK8/FOS/ADRB1	12
**56**	hsa04060	Cytokine-cytokine receptor interaction	14/109	294/7229	0.000122	0.000386	0.00014	FASLG/TNFSF15/IL1B/IL10RB/IL6/IL2/IFNGR1/IL4/CD40LG/CCL2/IL1A/CXCL11/CXCL2/CXCL10	14
**57**	hsa04923	Regulation of lipolysis in adipocytes	6/109	55/7229	0.000163	0.000506	0.000183	PTGS1/PTGS2/ADRB2/AKT1/PTGER3/ADRB1	6
**58**	hsa04140	Autophagy - animal	9/109	137/7229	0.000208	0.000634	0.00023	BCL2/AKT1/MAPK3/MAPK1/PRKCD/BCL2L1/MAPK8/HIF1A/CTSD	9
**59**	hsa04670	Leukocyte transendothelial migration	8/109	112/7229	0.000268	0.00079	0.000287	PRKCA/MMP2/MMP9/ICAM1/MAPK14/VCAM1/PRKCB/CLDN4	8
**60**	hsa04725	Cholinergic synapse	8/109	112/7229	0.000268	0.00079	0.000287	BCL2/AKT1/MAPK3/MAPK1/PRKCA/ACHE/FOS/PRKCB	8
**61**	hsa04929	GnRH secretion	6/109	64/7229	0.000377	0.001095	0.000397	AKT1/MAPK3/MAPK1/PRKCA/PRKCB/SPP1	6
**62**	hsa04261	Adrenergic signaling in cardiomyocytes	9/109	149/7229	0.000389	0.001111	0.000403	ADRB2/BCL2/AKT1/MAPK3/MAPK1/PRKCA/MAPK14/ADRA1B/ADRB1	9
**63**	hsa04920	Adipocytokine signaling pathway	6/109	69/7229	0.000568	0.001595	0.000579	RELA/AKT1/PPARA/RXRA/NFKBIA/MAPK8	6
**64**	hsa04622	RIG-I-like receptor signaling pathway	6/109	70/7229	0.000613	0.001696	0.000615	RELA/CASP8/NFKBIA/MAPK14/MAPK8/CXCL10	6
**65**	hsa04020	Calcium signaling pathway	10/109	193/7229	0.000626	0.001705	0.000619	NOS2/ADRB2/PRKCA/EGFR/ERBB2/PTGER3/ADRA1B/PRKCB/NOS3/ADRB1	10
**66**	hsa00982	Drug metabolism - cytochrome P450	6/109	72/7229	0.000713	0.001913	0.000694	GSTP1/CYP3A4/CYP1A2/GSTM1/ADH1C/MAOA	6
**67**	hsa04061	Viral protein interaction with cytokine and cytokine receptor	7/109	100/7229	0.000739	0.001952	0.000708	IL10RB/IL6/IL2/CCL2/CXCL11/CXCL2/CXCL10	7
**68**	hsa04650	Natural killer cell mediated cytotoxicity	8/109	131/7229	0.000769	0.001973	0.000716	CASP3/FASLG/MAPK3/MAPK1/PRKCA/ICAM1/IFNGR1/PRKCB	8

**69**	hsa04728	Dopaminergic synapse	8/109	131/7229	0.000769	0.001973	0.000716	AKT1/PRKCA/MAPK14/GSK3B/MAPK8/FOS/PRKCB/MAOA	8
**70**	hsa04913	Ovarian steroidogenesis	5/109	49/7229	0.0008	0.002022	0.000733	PTGS2/CYP19A1/CYP1A1/ALOX5/AKR1C3	5
**71**	hsa00980	Metabolism of xenobiotics by cytochrome P450	6/109	76/7229	0.000951	0.00237	0.00086	GSTP1/CYP3A4/CYP1A2/CYP1A1/GSTM1/ADH1C	6
**72**	hsa00140	Steroid hormone biosynthesis	5/109	60/7229	0.002009	0.004938	0.001791	CYP19A1/CYP3A4/CYP1A2/CYP1A1/AKR1C3	5
**73**	hsa04960	Aldosterone-regulated sodium reabsorption	4/109	37/7229	0.002202	0.00534	0.001937	MAPK3/MAPK1/PRKCA/PRKCB	4
**74**	hsa00590	Arachidonic acid metabolism	5/109	63/7229	0.002494	0.005887	0.002136	PTGS1/PTGS2/ALOX5/AKR1C3/LTA4H	5
**75**	hsa04623	Cytosolic DNA-sensing pathway	5/109	63/7229	0.002494	0.005887	0.002136	RELA/IL1B/IL6/NFKBIA/CXCL10	5
**76**	hsa04611	Platelet activation	7/109	124/7229	0.002587	0.006024	0.002185	PTGS1/AKT1/MAPK3/MAPK1/MAPK14/NOS3/COL1A1	7
**77**	hsa04666	Fc gamma R-mediated phagocytosis	6/109	93/7229	0.002697	0.006199	0.002249	AKT1/MAPK3/MAPK1/PRKCA/PRKCD/PRKCB	6
**78**	hsa04137	Mitophagy - animal	5/109	65/7229	0.002862	0.006495	0.002356	RELA/BCL2L1/JUN/MAPK8/HIF1A	5
**79**	hsa00380	Tryptophan metabolism	4/109	42/7229	0.003523	0.007894	0.002864	CAT/CYP1A2/CYP1A1/MAOA	4
**80**	hsa04914	Progesterone-mediated oocyte maturation	6/109	99/7229	0.003686	0.008116	0.002944	HSP90AA1/AKT1/MAPK3/MAPK1/MAPK14/MAPK8	6
**81**	hsa05031	Amphetamine addiction	5/109	69/7229	0.003714	0.008116	0.002944	PRKCA/JUN/FOS/PRKCB/MAOA	5
**82**	hsa04940	Type I diabetes mellitus	4/109	43/7229	0.00384	0.008262	0.002997	FASLG/IL1B/IL2/IL1A	4
**83**	hsa04750	Inflammatory mediator regulation of TRP channels	6/109	100/7229	0.003874	0.008262	0.002997	PRKCA/IL1B/PRKCD/MAPK14/MAPK8/PRKCB	6
**84**	hsa05010	Alzheimer disease	8/109	171/7229	0.004159	0.008763	0.003179	CASP3/CASP8/MAPK3/MAPK1/IL1B/CASP7/GSK3B/BACE1	8
**85**	hsa04015	Rap1 signaling pathway	9/109	210/7229	0.00429	0.008933	0.003241	AKT1/MAPK3/MAPK1/PRKCA/EGFR/VEGFA/MAPK14/EGF/PRKCB	9
**86**	hsa04371	Apelin signaling pathway	7/109	137/7229	0.004506	0.009274	0.003364	NOS2/AKT1/MAPK3/MAPK1/CCND1/NOS3/SPP1	7
**87**	hsa04930	Type II diabetes	4/109	46/7229	0.004906	0.00998	0.003621	MAPK3/MAPK1/PRKCD/MAPK8	4
**88**	hsa03320	PPAR signaling	5/109	76/7229	0.005619	0.011302	0.0041	PPARG/PPARA/RXRA/MMP1/PPARD	5
**89**	hsa04672	Intestinal immune network for IgA production	4/109	49/7229	0.006154	0.012238	0.00444	IL6/IL2/IL4/CD40LG	4
**90**	hsa00330	Arginine and proline metabolism	4/109	50/7229	0.006612	0.013004	0.004718	NOS2/ODC1/NOS3/MAOA	4
**91**	hsa04723	Retrograde endocannabinoid signaling	7/109	148/7229	0.006843	0.013309	0.004828	PTGS2/MAPK3/MAPK1/PRKCA/MAPK14/MAPK8/PRKCB	7
**92**	hsa04934	Cushing syndrome	7/109	155/7229	0.008738	0.016811	0.006099	CDKN1A/MAPK3/MAPK1/EGFR/CCND1/GSK3B/AHR	7
**93**	hsa00480	Glutathione metabolism	4/109	56/7229	0.009845	0.018737	0.006797	GSTP1/GSR/GSTM1/ODC1	4
**94**	hsa04310	Wnt signaling pathway	7/109	160/7229	0.010311	0.019416	0.007044	PRKCA/CCND1/JUN/GSK3B/MAPK8/PRKCB/PPARD	7
**95**	hsa04211	Longevity regulating pathway	5/109	89/7229	0.010827	0.020172	0.007318	RELA/BAX/AKT1/CAT/PPARG	5
**96**	hsa01523	Antifolate resistance	3/109	31/7229	0.01103	0.020336	0.007377	RELA/IL1B/IL6	3
**97**	hsa04970	Salivary secretion	5/109	90/7229	0.011329	0.020673	0.0075	ADRB2/PRKCA/ADRA1B/PRKCB/ADRB1	5
**98**	hsa04730	Long-term depression	4/109	60/7229	0.012486	0.022552	0.008181	MAPK3/MAPK1/PRKCA/PRKCB	4
**99**	hsa04022	cGMP-PKG signaling pathway	7/109	167/7229	0.012849	0.022973	0.008334	ADRB2/AKT1/MAPK3/MAPK1/ADRA1B/NOS3/ADRB1	7
**100**	hsa04270	Vascular smooth muscle contraction	6/109	132/7229	0.01447	0.025612	0.009291	MAPK3/MAPK1/PRKCA/PRKCD/ADRA1B/PRKCB	6
**101**	hsa04713	Circadian entrainment	5/109	97/7229	0.015299	0.026811	0.009726	MAPK3/MAPK1/PRKCA/FOS/PRKCB	5
**102**	hsa04910	Insulin signaling	6/109	137/7229	0.017106	0.029683	0.010768	FASN/AKT1/MAPK3/MAPK1/GSK3B/MAPK8	6
**103**	hsa04916	Melanogenesis	5/109	101/7229	0.01794	0.030547	0.011081	MAPK3/MAPK1/PRKCA/GSK3B/PRKCB	5
**104**	hsa00830	Retinol metabolism	4/109	67/7229	0.018121	0.030547	0.011081	CYP3A4/CYP1A2/CYP1A1/ADH1C	4
**105**	hsa04720	Long-term potentiation	4/109	67/7229	0.018121	0.030547	0.011081	MAPK3/MAPK1/PRKCA/PRKCB	4
**106**	hsa04520	Adherens junction	4/109	71/7229	0.021949	0.036652	0.013296	MAPK3/MAPK1/EGFR/ERBB2	4
**107**	hsa04976	Bile secretion	4/109	72/7229	0.022978	0.038011	0.013789	HMGCR/RXRA/CYP3A4/CA2	4
**108**	hsa04072	Phospholipase D signaling pathway	6/109	148/7229	0.024033	0.039388	0.014289	AKT1/MAPK3/MAPK1/PRKCA/EGFR/EGF	6
**109**	hsa04918	Thyroid hormone synthesis	4/109	74/7229	0.025123	0.040795	0.014799	GSR/PRKCA/PRKCB/ALB	4
**110**	hsa04150	mTOR signaling pathway	6/109	153/7229	0.027728	0.044617	0.016186	AKT1/MAPK3/MAPK1/PRKCA/GSK3B/PRKCB	6
**111**	hsa00983	Drug metabolism - other enzymes	4/109	79/7229	0.030999	0.04943	0.017932	GSTP1/CYP3A4/GSTM1/MPO	4

AD: atopic dermatitis. OB: oral bioavailability; DL: drug-likeness

Table S7. The enriched KEGG pathways for intersection targets between compound (OB < 30% and DL ⩾ 0.18) and AD related targets

	ID	Description	GeneRati	BgRatio	pvalue	p.adjust	qvalue	geneID	Count
1	hsa04657	IL-17 signaling pathway	23/103	94/7946	6.99E-24	1.62E-21	5.23E-22	PTGS2/RELA/MAPK1/CASP3/CCL2/IL4/CCL11/HSP90AA1/MMP9/MMP1/IL1B/CSF2/FOS/JUN/IL6/MAPK8/NFKBIA/CASP8/MMP3/IKBKG/IL13/MAPK14/GSK3B	23
2	hsa05167	Kaposi sarcoma-associated herpesvirus infection	28/103	186/7946	8.21E-23	9.52E-21	3.07E-21	PTGS2/RELA/CDKN1A/MAPK1/CASP3/STAT1/CD86/CCR3/C3/ICAM1/CSF2/STAT3/VEGFA/CCND1/FOS/BAX/JUN/IL6/MAPK8/NFKBIA/CASP8/CREB1/AKT1/HIF1A/IFNGR1/IKBKG/MAPK14/GSK3B	28
3	hsa04933	AGE-RAGE signaling pathway in diabetic complications	22/103	100/7946	9.40E-22	7.27E-20	2.34E-20	RELA/MAPK1/CASP3/STAT1/CCL2/ICAM1/IL1B/PRKCD/STAT3/VEGFA/CCND1/BCL2/BAX/MMP2/JUN/IL6/MAPK8/SELE/NOS3/AKT1/MAPK14/PRKC	22
4	hsa04668	TNF signaling pathway	21/103	112/7946	3.08E-19	1.79E-17	5.76E-18	PTGS2/RELA/MAPK1/CASP3/CCL2/ICAM1/MMP9/IL1B/CSF2/FOS/JUN/IL6/MAPK8/NFKBIA/CASP8/MMP3/CREB1/SELE/AKT1/IKBKG/MAPK14	21
5	hsa05142	Chagas disease (American trypanosomiasis)	20/103	102/7946	9.30E-19	4.32E-17	1.39E-17	NOS2/RELA/MAPK1/CCL2/IL2/CCL3/CD247/C3/IL1B/FOS/JUN/IL6/MAPK8/NFKBIA/CASP8/FASLG/AKT1/IFNGR1/IKBKG/MAPK14	20
6	hsa05215	Prostate cancer	19/103	97/7946	7.62E-18	2.95E-16	9.49E-17	AR/RELA/CDKN1A/MAPK1/HSP90AA1/MMP9/EGF/PLAU/CCND1/BCL2/NFKBIA/MMP3/CREB1/AKT1/MDM2/IKBKG/INS/GSK3B/GSTP1	19
7	hsa05418	Fluid shear stress and atherosclerosis	21/103	139/7946	3.31E-17	1.10E-15	3.53E-16	RELA/CCL2/HSP90AA1/HMOX1/ICAM1/NQO1/MMP9/IL1B/VEGFA/BCL2/FOS/MMP2/JUN/MAPK8/SELE/PECAM1/NOS3/AKT1/IKBKG/MAPK14/GSTP1	21
8	hsa05163	Human cytomegalovirus infection	25/103	225/7946	4.28E-17	1.24E-15	4.00E-16	PTGS2/RELA/CDKN1A/MAPK1/CASP3/CCL2/CCL3/CCR3/IL1B/STAT3/VEGFA/CCND1/BAX/IL6/NFKBIA/CASP8/CREB1/PTGER3/FASLG/AKT1/MDM2/IKBKG/MAPK14/GSK3B/PRKCB	25
9	hsa04659	Th17 cell differentiation	19/103	107/7946	5.36E-17	1.38E-15	4.45E-16	RELA/MAPK1/STAT1/IL2/IL4/CD247/HSP90AA1/RXRA/IL1B/STAT3/FOS/JUN/IL6/MAPK8/NFKBIA/HIF1A/IFNGR1/IKBKG/MAPK14	19
10	hsa05162	Measles	20/103	138/7946	4.72E-16	1.09E-14	3.53E-15	RELA/CASP3/STAT1/IL2/IL1B/STAT3/CCND1/BCL2/BCL2L1/FOS/BAX/JUN/IL6/MAPK8/NFKBIA/CASP8/FASLG/AKT1/IKBKG/GSK3B	20
11	hsa05161	Hepatitis B	21/103	162/7946	8.28E-16	1.75E-14	5.63E-15	RELA/CDKN1A/MAPK1/CASP3/STAT1/MMP9/STAT3/BCL2/FOS/BAX/JUN/IL6/MAPK8/NFKBIA/CASP8/CREB1/FASLG/AKT1/IKBKG/MAPK14/PRKCB	21
12	hsa05140	Leishmaniasis	16/103	77/7946	1.37E-15	2.65E-14	8.52E-15	NOS2/PTGS2/RELA/MAPK1/STAT1/IL4/C3/IL1B/FOS/JUN/NFKBIA/PTPN6/IFNGR1/MAPK14/PRKCB/ITGB2	16
13	hsa05145	Toxoplasmosis	18/103	112/7946	2.43E-15	4.34E-14	1.40E-14	NOS2/RELA/MAPK1/CASP3/STAT1/STAT3/BCL2/BCL2L1/MAPK8/NFKBIA/CASP8/AKT1/IFNGR1/IKBKG/XIAP/CD40LG/MAPK14/ALOX5	18
14	hsa04625	C-type lectin receptor signaling pathway	17/103	104/7946	1.16E-14	1.92E-13	6.18E-14	PTGS2/RELA/MAPK1/STAT1/IL2/IL1B/PRKCD/JUN/IL6/MAPK8/NFKBIA/CASP8/CASP1/AKT1/MDM2/IKBKG/MAPK14	17
15	hsa04620	Toll-like receptor signaling pathway	16/103	104/7946	2.00E-13	2.91E-12	9.36E-13	RELA/MAPK1/STAT1/CCL3/CD80/CD86/IL1B/FOS/JUN/IL6/MAPK8/NFKBIA/CASP8/AKT1/IKBKG/MAPK14	16
16	hsa04660	T cell receptor signaling pathway	16/103	104/7946	2.00E-13	2.91E-12	9.36E-13	RELA/MAPK1/IL2/IL4/CD247/CSF2/FOS/JUN/MAPK8/NFKBIA/PTPN6/AKT1/IKBKG/CD40LG/MAPK14/GSK3B	16
17	hsa05235	PD-L1 expression and PD-1 checkpoint pathway in cancer	15/103	89/7946	2.99E-13	4.08E-12	1.31E-12	RELA/MAPK1/STAT1/CD247/EGF/STAT3/FOS/JUN/NFKBIA/PTPN6/AKT1/HIF1A/IFNGR1/IKBKG/MAPK14	15
18	hsa04066	HIF-1 signaling pathway	16/103	109/7946	4.28E-13	5.51E-12	1.78E-12	NOS2/RELA/CDKN1A/MAPK1/HMOX1/EGF/STAT3/VEGFA/BCL2/IL6/NOS3/AKT1/HIF1A/IFNGR1/INS/PRKCB	16
19	hsa05133	Pertussis	14/103	76/7946	5.42E-13	6.62E-12	2.13E-12	NOS2/RELA/MAPK1/CASP3/C3/C5/IL1B/FOS/JUN/IL6/MAPK8/CASP1/MAPK14/ITGB2	14
20	hsa05169	Epstein-Barr virus infection	20/103	201/7946	7.34E-13	8.51E-12	2.74E-12	RELA/CDKN1A/CASP3/STAT1/CD247/ICAM1/STAT3/CCND1/BCL2/BAX/JUN/IL6/MAPK8/NFKBIA/CASP8/AKT1/MDM2/IKBKG/FCER2/MAPK14	20
21	hsa04658	Th1 and Th2 cell differentiation	14/103	92/7946	8.39E-12	8.85E-11	2.85E-11	RELA/MAPK1/STAT1/IL2/IL4/CD247/FOS/JUN/MAPK8/NFKBIA/IFNGR1/IKBKG/IL13/MAPK14	14
22	hsa05222	Small cell lung cancer	14/103	92/7946	8.39E-12	8.85E-11	2.85E-11	NOS2/PTGS2/RELA/CDKN1A/CASP3/RXRA/CCND1/BCL2/BCL2L1/BAX/NFKBIA/AKT1/IKBKG/XIAP	14
23	hsa05323	Rheumatoid arthritis	14/103	93/7946	9.77E-12	9.86E-11	3.18E-11	CCL2/CCL3/CD80/CD86/ICAM1/MMP1/IL1B/CSF2/VEGFA/FOS/JUN/IL6/MMP3/ITGB2	14
24	hsa05212	Pancreatic cancer	13/103	76/7946	1.05E-11	1.01E-10	3.26E-11	RELA/CDKN1A/MAPK1/STAT1/EGF/STAT3/VEGFA/CCND1/BCL2L1/BAX/MAPK8/AKT1/IKBKG	13
25	hsa04621	NOD-like receptor signaling pathway	18/103	181/7946	1.19E-11	1.11E-10	3.56E-11	RELA/MAPK1/STAT1/CCL2/HSP90AA1/IL1B/PRKCD/BCL2/BCL2L1/JUN/IL6/MAPK8/NFKBIA/CASP8/CASP1/IKBKG/XIAP/MAPK14	18
26	hsa04210	Apoptosis	16/103	136/7946	1.41E-11	1.26E-10	4.06E-11	RELA/MAPK1/CASP3/BCL2/BCL2L1/FOS/BAX/JUN/MAPK8/NFKBIA/CASP8/MCL1/FASLG/AKT1/IKBKG/XIAP	16
27	hsa04630	JAK-STAT signaling pathway	17/103	162/7946	1.98E-11	1.66E-10	5.33E-11	CDKN1A/STAT1/IL2/IL4/IL5RA/EGF/CSF2/STAT3/CCND1/BCL2/BCL2L1/IL6/MCL1/PTPN6/AKT1/IFNGR1/IL13	17

**28**	hsa01522	Endocrine resistance	14/103	98/7946	2.04E-11	1.66E-10	5.33E-11	ESR1/CDKN1A/MAPK1/MMP9/CCND1/BCL2/FOS/BAX/MMP2/JUN/MAPK8/AKT1/MDM2/MAPK14	14
**29**	hsa05132	Salmonella infection	13/103	80/7946	2.07E-11	1.66E-10	5.33E-11	NOS2/RELA/MAPK1/CCL3/IL1B/CSF2/FOS/JUN/IL6/MAPK8/CASP1/IFNGR1/MAPK14	13
**30**	hsa05135	Yersinia infection	15/103	120/7946	2.67E-11	2.07E-10	6.66E-11	RELA/MAPK1/CCL2/IL2/IL1B/FOS/JUN/IL6/MAPK8/NFKBIA/CASP1/AKT1/IKBKG/MAPK14/GSK3B	15
**31**	hsa05166	Human T-cell leukemia virus 1 infection	19/103	219/7946	3.38E-11	2.53E-10	8.15E-11	RELA/CDKN1A/MAPK1/IL2/ICAM1/CSF2/CCND1/BCL2L1/FOS/BAX/JUN/IL6/MAPK8/NFKBIA/CREB1/AKT1/IKBKG/XIAP/ITGB2	19
**32**	hsa05164	Influenza A	17/103	170/7946	4.32E-11	3.13E-10	1.01E-10	RELA/MAPK1/CASP3/STAT1/CCL2/ICAM1/IL1B/BAX/IL6/NFKBIA/CASP8/FASLG/CASP1/AKT1/IFNGR1/IKBKG/PRKCB	17
**33**	hsa04917	Prolactin signaling pathway	12/103	70/7946	6.68E-11	4.70E-10	1.51E-10	ESR1/RELA/MAPK1/STAT1/STAT3/CCND1/FOS/MAPK8/AKT1/INS/MAPK14/GSK3B	12
**34**	hsa04926	Relaxin signaling pathway	15/103	129/7946	7.69E-11	5.25E-10	1.69E-10	NOS2/RELA/MAPK1/MMP9/MMP1/VEGFA/FOS/MMP2/JUN/MAPK8/NFKBIA/CREB1/NOS3/AKT1/MAPK14	15
**35**	hsa04151	PI3K-Akt signaling pathway	23/103	354/7946	8.45E-11	5.60E-10	1.80E-10	RELA/CDKN1A/MAPK1/IL2/IL4/HSP90AA1/RXRA/EGF/FLT4/VEGFA/CCND1/BCL2/BCL2L1/IL6/CREB1/MCL1/NOS3/FASLG/AKT1/MDM2/IKBKG/INS/GSK3B	23
**36**	hsa05160	Hepatitis C	16/103	155/7946	1.05E-10	6.75E-10	2.18E-10	RELA/CDKN1A/MAPK1/CASP3/STAT1/RXRA/EGF/STAT3/CCND1/BAX/NFKBIA/CASP8/FASLG/AKT1/IKBKG/GSK3B	16
**37**	hsa05152	Tuberculosis	17/103	180/7946	1.08E-10	6.75E-10	2.18E-10	NOS2/RELA/MAPK1/CASP3/STAT1/C3/IL1B/BCL2/BAX/IL6/MAPK8/CASP8/CREB1/AKT1/IFNGR1/MAPK14/ITGB2	17
**38**	hsa01524	Platinum drug resistance	12/103	73/7946	1.12E-10	6.83E-10	2.20E-10	CDKN1A/MAPK1/CASP3/BCL2/BCL2L1/BAX/CASP8/FASLG/AKT1/MDM2/XIAP/GSTP1	12
**39**	hsa05130	Pathogenic Escherichia coli infection	17/103	202/7946	6.63E-10	3.95E-09	1.27E-09	RELA/MAPK1/CASP3/F2/IL1B/FOS/BAX/JUN/IL6/MAPK8/NFKBIA/CASP8/PTPN6/FASLG/CASP1/IKBKG/MAPK14	17
**40**	hsa05205	Proteoglycans in cancer	17/103	204/7946	7.73E-10	4.47E-09	1.44E-09	ESR1/CDKN1A/MAPK1/CASP3/MMP9/PLAU/STAT3/VEGFA/CCND1/MMP2/PTPN6/FASLG/AKT1/MDM2/HIF1A/MAPK14/PRKCB	17
**41**	hsa04380	Osteoclast differentiation	14/103	128/7946	7.91E-10	4.47E-09	1.44E-09	PPARG/RELA/MAPK1/STAT1/IL1B/FOS/JUN/MAPK8/NFKBIA/CREB1/AKT1/IFNGR1/IKBKG/MAPK14	14
**42**	hsa05210	Colorectal cancer	12/103	86/7946	8.10E-10	4.47E-09	1.44E-09	CDKN1A/MAPK1/CASP3/EGF/CCND1/BCL2/FOS/BAX/JUN/MAPK8/AKT1/GSK3B	12
**43**	hsa05131	Shigellosis	18/103	236/7946	9.84E-10	5.31E-09	1.71E-09	RELA/MAPK1/C3/IL1B/PRKCD/CSF2/BCL2/BCL2L1/BAX/JUN/MAPK8/NFKBIA/CASP1/AKT1/MDM2/IKBKG/MAPK14/GSK3B	18
**44**	hsa05170	Human immunodeficiency virus 1 infection	17/103	212/7946	1.40E-09	7.41E-09	2.39E-09	RELA/MAPK1/CASP3/CD247/BCL2/BCL2L1/FOS/BAX/JUN/MAPK8/NFKBIA/CASP8/FASLG/AKT1/IKBKG/MAPK14/PRKCB	17
**45**	hsa05219	Bladder cancer	9/103	41/7946	1.80E-09	9.29E-09	2.99E-09	CDKN1A/MAPK1/MMP9/EGF/MMP1/VEGFA/CCND1/MMP2/MDM2	9
**46**	hsa01521	EGFR tyrosine kinase inhibitor resistance	11/103	79/7946	4.39E-09	2.21E-08	7.13E-09	MAPK1/EGF/STAT3/VEGFA/BCL2/BCL2L1/BAX/IL6/AKT1/GSK3B/PRKCB	11
**47**	hsa04064	NF-kappa B signaling pathway	12/103	102/7946	6.03E-09	2.98E-08	9.59E-09	PTGS2/RELA/ICAM1/IL1B/PLAU/BCL2/BCL2L1/NFKBIA/IKBKG/XIAP/CD40LG/PRKCB	12
**48**	hsa05321	Inflammatory bowel disease (IBD)	10/103	65/7946	8.52E-09	4.12E-08	1.33E-08	RELA/STAT1/IL2/IL4/IL1B/STAT3/JUN/IL6/IFNGR1/IL13	10
**49**	hsa04068	FoxO signaling pathway	13/103	131/7946	1.11E-08	5.25E-08	1.69E-08	CDKN1A/MAPK1/EGF/STAT3/CCND1/IL6/MAPK8/FASLG/AKT1/MDM2/INS/MAPK14/CAT	13
**50**	hsa04931	Insulin resistance	12/103	108/7946	1.17E-08	5.42E-08	1.74E-08	RELA/PRKCD/STAT3/IL6/MAPK8/NFKBIA/CREB1/NOS3/AKT1/INS/GSK3B/PRKCB	12
**51**	hsa04915	Estrogen signaling pathway	13/103	138/7946	2.09E-08	9.50E-08	3.06E-08	ESR1/MAPK1/HSP90AA1/MMP9/PRKCD/BCL2/FOS/MMP2/JUN/CREB1/NOS3/AKT1/OPRM1	13
**52**	hsa04722	Neurotrophin signaling pathway	12/103	119/7946	3.53E-08	1.58E-07	5.07E-08	RELA/MAPK1/PRKCD/BCL2/BAX/JUN/MAPK8/NFKBIA/FASLG/AKT1/MAPK14/GSK3B	12
**53**	hsa05203	Viral carcinogenesis	15/103	201/7946	3.80E-08	1.66E-07	5.35E-08	RELA/CDKN1A/MAPK1/CASP3/CCR3/C3/STAT3/CCND1/BAX/JUN/NFKBIA/CASP8/CREB1/MDM2/IKBKG	15
**54**	hsa05134	Legionellosis	9/103	57/7946	3.90E-08	1.67E-07	5.39E-08	RELA/CASP3/C3/IL1B/IL6/NFKBIA/CASP8/CASP1/ITGB2	9
**55**	hsa05220	Chronic myeloid leukemia	10/103	76/7946	4.04E-08	1.70E-07	5.49E-08	RELA/CDKN1A/MAPK1/CCND1/BCL2L1/BAX/NFKBIA/AKT1/MDM2/IKBKG	10
**56**	hsa04932	Non-alcoholic fatty liver disease (NAFLD)	13/103	149/7946	5.26E-08	2.18E-07	7.02E-08	RELA/CASP3/RXRA/IL1B/BAX/JUN/IL6/MAPK8/CASP8/FASLG/AKT1/INS/GSK3B	13
**57**	hsa04662	B cell receptor signaling pathway	10/103	82/7946	8.48E-08	3.45E-07	1.11E-07	RELA/MAPK1/FOS/JUN/NFKBIA/PTPN6/AKT1/IKBKG/GSK3B/PRKCB	10
**58**	hsa04062	Chemokine signaling pathway	14/103	189/7946	1.24E-07	4.94E-07	1.59E-07	RELA/MAPK1/STAT1/CCL2/CCL3/CCL11/CCR3/PRKCD/STAT3/NFKBIA/AKT1/IKBKG/GSK3B/PRKCB	14
**59**	hsa04215	Apoptosis - multiple species	7/103	32/7946	1.29E-07	5.08E-07	1.64E-07	CASP3/BCL2/BCL2L1/BAX/MAPK8/CASP8/XIAP	7
**60**	hsa05223	Non-small cell lung cancer	9/103	66/7946	1.46E-07	5.63E-07	1.81E-07	CDKN1A/MAPK1/RXRA/EGF/STAT3/CCND1/BAX/AKT1/PRKCB	9
**61**	hsa04664	Fc epsilon RI signaling pathway	9/103	68/7946	1.90E-07	7.22E-07	2.32E-07	MAPK1/IL4/CSF2/MAPK8/AKT1/IL13/MS4A2/MAPK14/ALOX5	9
**62**	hsa05144	Malaria	8/103	50/7946	2.07E-07	7.76E-07	2.50E-07	CCL2/ICAM1/IL1B/IL6/SELE/PECAM1/CD40LG/ITGB2	8

**63**	hsa05014	Amyotrophic lateral sclerosis (ALS)	8/103	51/7946	2.43E-07	8.96E-07	2.89E-07	CASP3/BCL2/BCL2L1/BAX/CASP1/MAPK14/SOD1/CAT	8
**64**	hsa05143	African trypanosomiasis	7/103	37/7946	3.75E-07	1.36E-06	4.38E-07	IDO1/ICAM1/IL1B/IL6/SELE/FASLG/PRKCB	7
**65**	hsa05202	Transcriptional misregulation in cancer	13/103	186/7946	7.07E-07	2.52E-06	8.13E-07	PPARG/RELA/CDKN1A/CD86/RXRA/MMP9/CSF2/PLAU/BCL2L1/BAX/IL6/MMP3/MDM2	13
**66**	hsa05416	Viral myocarditis	8/103	60/7946	8.88E-07	3.12E-06	1.01E-06	CASP3/CD80/CD86/ICAM1/CCND1/CASP8/CD40LG/ITGB2	8
**67**	hsa05165	Human papillomavirus infection	17/103	330/7946	9.55E-07	3.31E-06	1.07E-06	PTGS2/RELA/CDKN1A/MAPK1/CASP3/STAT1/EGF/VEGFA/CCND1/BAX/CASP8/CREB1/FASLG/AKT1/MDM2/IKBKG/GSK3B	17
**68**	hsa04010	MAPK signaling pathway	16/103	295/7946	1.05E-06	3.55E-06	1.14E-06	RELA/MAPK1/CASP3/EGF/IL1B/FLT4/VEGFA/FOS/JUN/MAPK8/FASLG/AKT1/IKBKG/INS/MAPK14/PRKCB	16
**69**	hsa04217	Necroptosis	12/103	162/7946	1.06E-06	3.55E-06	1.14E-06	STAT1/HSP90AA1/IL1B/STAT3/BCL2/BAX/MAPK8/CASP8/FASLG/CASP1/IFNGR1/XIAP	12
**70**	hsa05224	Breast cancer	11/103	147/7946	2.77E-06	9.02E-06	2.90E-06	ESR1/CDKN1A/MAPK1/EGF/FLT4/CCND1/FOS/BAX/JUN/AKT1/GSK3B	11
**71**	hsa04071	Sphingolipid signaling pathway	10/103	119/7946	2.84E-06	9.02E-06	2.90E-06	RELA/MAPK1/BCL2/BAX/MAPK8/NOS3/AKT1/MS4A2/MAPK14/PRKCB	10
**72**	hsa04919	Thyroid hormone signaling pathway	10/103	119/7946	2.84E-06	9.02E-06	2.90E-06	ESR1/MAPK1/STAT1/RXRA/CCND1/AKT1/MDM2/HIF1A/GSK3B/PRKCB	10
**73**	hsa04935	Growth hormone synthesis, secretion and action	10/103	119/7946	2.84E-06	9.02E-06	2.90E-06	MAPK1/STAT1/STAT3/FOS/MAPK8/CREB1/AKT1/MAPK14/GSK3B/PRKCB	10
**74**	hsa04115	p53 signaling pathway	8/103	72/7946	3.66E-06	1.15E-05	3.69E-06	CDKN1A/CASP3/CCND1/BCL2/BCL2L1/BAX/CASP8/MDM2	8
**75**	hsa05020	Prion diseases	6/103	35/7946	4.90E-06	1.52E-05	4.89E-06	MAPK1/C5/IL1B/BAX/IL6/SOD1	6
**76**	hsa05214	Glioma	8/103	75/7946	4.99E-06	1.52E-05	4.89E-06	CDKN1A/MAPK1/EGF/CCND1/BAX/AKT1/MDM2/PRKCB	8
**77**	hsa04060	Cytokine-cytokine receptor interaction	15/103	294/7946	5.04E-06	1.52E-05	4.89E-06	CCL2/IL2/IL4/IL5RA/CCL3/CCL11/CCR3/TNFSF15/IL1B/CSF2/IL6/FASLG/IFNGR1/CD40LG/IL13	15
**78**	hsa04923	Regulation of lipolysis in adipocytes	7/103	55/7946	6.10E-06	1.81E-05	5.85E-06	PTGS2/PTGS1/ADRB2/PTGER3/AKT1/INS/ADRB1	7
**79**	hsa04650	Natural killer cell mediated cytotoxicity	10/103	131/7946	6.76E-06	1.99E-05	6.40E-06	MAPK1/CASP3/CD247/ICAM1/CSF2/PTPN6/FASLG/IFNGR1/PRKCB/ITGB2	10
**80**	hsa05216	Thyroid cancer	6/103	37/7946	6.88E-06	1.99E-05	6.43E-06	PPARG/CDKN1A/MAPK1/RXRA/CCND1/BAX	6
**81**	hsa05330	Allograft rejection	6/103	38/7946	8.08E-06	2.31E-05	7.46E-06	IL2/IL4/CD80/CD86/FASLG/CD40LG	6
**82**	hsa05213	Endometrial cancer	7/103	58/7946	8.76E-06	2.48E-05	7.98E-06	CDKN1A/MAPK1/EGF/CCND1/BAX/AKT1/GSK3B	7
**83**	hsa04510	Focal adhesion	12/103	199/7946	9.07E-06	2.53E-05	8.16E-06	MAPK1/EGF/FLT4/VEGFA/CCND1/BCL2/JUN/MAPK8/AKT1/XIAP/GSK3B/PRKCB	12
**84**	hsa05206	MicroRNAs in cancer	15/103	310/7946	9.58E-06	2.65E-05	8.53E-06	PTGS2/CDKN1A/MAPK1/CASP3/HMOX1/MMP9/TP63/PLAU/STAT3/VEGFA/CCND1/BCL2/MCL1/MDM2/PRKCB	15
**85**	hsa04370	VEGF signaling pathway	7/103	59/7946	9.83E-06	2.68E-05	8.64E-06	PTGS2/MAPK1/VEGFA/NOS3/AKT1/MAPK14/PRKCB	7
**86**	hsa05225	Hepatocellular carcinoma	11/103	168/7946	1.01E-05	2.72E-05	8.77E-06	CDKN1A/MAPK1/HMOX1/NQO1/CCND1/BCL2L1/BAX/AKT1/GSK3B/PRKCB/GSTP1	11
**87**	hsa05332	Graft-versus-host disease	6/103	41/7946	1.28E-05	3.37E-05	1.09E-05	IL2/CD80/CD86/IL1B/IL6/FASLG	6
**88**	hsa04012	ErbB signaling pathway	8/103	85/7946	1.28E-05	3.37E-05	1.09E-05	CDKN1A/MAPK1/EGF/JUN/MAPK8/AKT1/GSK3B/PRKCB	8
**89**	hsa04726	Serotonergic synapse	9/103	115/7946	1.64E-05	4.27E-05	1.37E-05	PTGS2/MAPK1/CASP3/PTGS1/HTR3A/MAOA/SLC6A4/PRKCB/ALOX5	9
**90**	hsa04940	Type I diabetes mellitus	6/103	43/7946	1.69E-05	4.37E-05	1.41E-05	IL2/CD80/CD86/IL1B/FASLG/INS	6
**91**	hsa05221	Acute myeloid leukemia	7/103	67/7946	2.30E-05	5.87E-05	1.89E-05	RELA/MAPK1/CSF2/STAT3/CCND1/AKT1/IKBKG	7
**92**	hsa04920	Adipocytokine signaling pathway	7/103	69/7946	2.80E-05	7.05E-05	2.27E-05	RELA/RXRA/STAT3/MAPK8/NFKBIA/AKT1/IKBKG	7
**93**	hsa05120	Epithelial cell signaling in Helicobacter pylori infection	7/103	70/7946	3.07E-05	7.67E-05	2.47E-05	RELA/CASP3/JUN/MAPK8/NFKBIA/IKBKG/MAPK14	7
**94**	hsa05231	Choline metabolism in cancer	8/103	98/7946	3.64E-05	8.82E-05	2.84E-05	MAPK1/EGF/FOS/JUN/MAPK8/AKT1/HIF1A/PRKCB	8
**95**	hsa04672	Intestinal immune network for IgA production	6/103	49/7946	3.65E-05	8.82E-05	2.84E-05	IL2/IL4/CD80/CD86/IL6/CD40LG	6
**96**	hsa04913	Ovarian steroidogenesis	6/103	49/7946	3.65E-05	8.82E-05	2.84E-05	PTGS2/CYP1A1/CYP19A1/INS/AKR1C3/ALOX5	6
**97**	hsa05218	Melanoma	7/103	72/7946	3.70E-05	8.85E-05	2.85E-05	CDKN1A/MAPK1/EGF/CCND1/BAX/AKT1/MDM2	7
**98**	hsa04014	Ras signaling pathway	12/103	232/7946	4.21E-05	9.97E-05	3.21E-05	RELA/MAPK1/EGF/FLT4/VEGFA/BCL2L1/MAPK8/FASLG/AKT1/IKBKG/INS/PRKCB	12
**99**	hsa05310	Asthma	5/103	31/7946	4.31E-05	0.000101	3.26E-05	IL4/CCL11/CD40LG/IL13/MS4A2	5
**100**	hsa05146	Amoebiasis	8/103	102/7946	4.86E-05	0.000113	3.64E-05	NOS2/RELA/CASP3/IL1B/CSF2/IL6/PRKCB/ITGB2	8
**101**	hsa05320	Autoimmune thyroid disease	6/103	53/7946	5.74E-05	0.000132	4.25E-05	IL2/IL4/CD80/CD86/FASLG/CD40LG	6
**102**	hsa04024	cAMP signaling pathway	11/103	216/7946	0.000103	0.000235	7.56E-05	RELA/MAPK1/ADRB2/FOS/JUN/MAPK8/NFKBIA/CREB1/PTGER3/AKT1/ADRB1	11
**103**	hsa05226	Gastric cancer	9/103	149/7946	0.000126	0.000284	9.15E-05	CDKN1A/MAPK1/RXRA/EGF/CCND1/BCL2/BAX/AKT1/GSK3B	9
**104**	hsa04211	Longevity regulating pathway	7/103	89/7946	0.000145	0.000322	0.000104	PPARG/RELA/BAX/CREB1/AKT1/INS/CAT	7
**105**	hsa04623	Cytosolic DNA-sensing pathway	6/103	63/7946	0.000153	0.000338	0.000109	RELA/IL1B/IL6/NFKBIA/CASP1/IKBKG	6

**106**	hsa05168	Herpes simplex virus 1 infection	17/103	491/7946	0.00017	0.000373	0.00012	RELA/CASP3/STAT1/CCL2/C3/C5/IL1B/BCL2/BCL2L1/BAX/IL6/NFKBIA/CASP8/FASLG/AKT1/IFNGR1/IKBKG	17
**107**	hsa04912	GnRH signaling pathway	7/103	93/7946	0.000191	0.000413	0.000133	MAPK1/PRKCD/MMP2/JUN/MAPK8/MAPK14/PRKCB	7
**108**	hsa05211	Renal cell carcinoma	6/103	69/7946	0.000254	0.000545	0.000176	CDKN1A/MAPK1/VEGFA/JUN/AKT1/HIF1A	6
**109**	hsa04622	RIG-I-like receptor signaling pathway	6/103	70/7946	0.000275	0.000585	0.000188	RELA/MAPK8/NFKBIA/CASP8/IKBKG/MAPK14	6
**110**	hsa04728	Dopaminergic synapse	8/103	131/7946	0.000282	0.000594	0.000191	FOS/MAPK8/CREB1/AKT1/MAOA/MAPK14/GSK3B/PRKCB	8
**111**	hsa04140	Autophagy - animal	8/103	137/7946	0.000382	0.000798	0.000257	MAPK1/PRKCD/BCL2/BCL2L1/MAPK8/AKT1/HIF1A/INS	8
**112**	hsa04928	Parathyroid hormone synthesis, secretion and action	7/103	106/7946	0.000427	0.000885	0.000285	CDKN1A/MAPK1/RXRA/BCL2/FOS/CREB1/PRKCB	7
**113**	hsa04670	Leukocyte transendothelial migration	7/103	112/7946	0.000596	0.001213	0.000391	ICAM1/MMP9/MMP2/PECAM1/MAPK14/PRKCB/ITGB2	7
**114**	hsa04725	Cholinergic synapse	7/103	112/7946	0.000596	0.001213	0.000391	MAPK1/BCL2/FOS/CREB1/AKT1/ACHE/PRKCB	7
**115**	hsa01523	Antifolate resistance	4/103	31/7946	0.00064	0.001291	0.000416	RELA/IL1B/IL6/IKBKG	4
**116**	hsa04261	Adrenergic signaling in cardiomyocytes	8/103	149/7946	0.000669	0.001339	0.000431	MAPK1/ADRA1B/ADRB2/BCL2/CREB1/AKT1/MAPK14/ADRB1	8
**117**	hsa04610	Complement and coagulation cascades	6/103	85/7946	0.000783	0.001553	0.0005	C3/F2/C5/PLAU/C5AR1/ITGB2	6
**118**	hsa04921	Oxytocin signaling pathway	8/103	153/7946	0.000797	0.001567	0.000505	PTGS2/CDKN1A/MAPK1/CCND1/FOS/JUN/NOS3/PRKCB	8
**119**	hsa04152	AMPK signaling pathway	7/103	120/7946	0.0009	0.001754	0.000565	PPARG/CCND1/FASN/CREB1/AKT1/INS/HMGCR	7
**120**	hsa04218	Cellular senescence	8/103	160/7946	0.001067	0.002063	0.000665	RELA/CDKN1A/MAPK1/CCND1/IL6/AKT1/MDM2/MAPK14	8
**121**	hsa04611	Platelet activation	7/103	124/7946	0.001091	0.002093	0.000674	MAPK1/F2/PTGS1/NOS3/AKT1/MAPK14/TBXA2R	7
**122**	hsa00590	Arachidonic acid metabolism	5/103	63/7946	0.001286	0.002445	0.000788	PTGS2/PTGS1/AKR1C3/LTA4H/ALOX5	5
**123**	hsa04022	cGMP-PKG signaling pathway	8/103	167/7946	0.001406	0.002652	0.000854	MAPK1/ADRA1B/ADRB2/CREB1/NOS3/AKT1/INS/ADRB1	8
**124**	hsa04137	Mitophagy - animal	5/103	65/7946	0.001481	0.00277	0.000892	RELA/BCL2L1/JUN/MAPK8/HIF1A	5
**125**	hsa04015	Rap1 signaling pathway	9/103	210/7946	0.00155	0.002877	0.000927	MAPK1/EGF/FLT4/VEGFA/AKT1/INS/MAPK14/PRKCB/ITGB2	9
**126**	hsa04640	Hematopoietic cell lineage	6/103	99/7946	0.001735	0.00317	0.001021	IL4/IL5RA/IL1B/CSF2/IL6/FCER2	6
**127**	hsa04914	Progesterone-mediated oocyte maturation	6/103	99/7946	0.001735	0.00317	0.001021	MAPK1/HSP90AA1/MAPK8/AKT1/INS/MAPK14	6
**128**	hsa04061	Viral protein interaction with cytokine and cytokine receptor	6/103	100/7946	0.001827	0.003311	0.001067	CCL2/IL2/CCL3/CCL11/CCR3/IL6	6
**129**	hsa05031	Amphetamine addiction	5/103	69/7946	0.001934	0.003478	0.00112	FOS/JUN/CREB1/MAOA/PRKCB	5
**130**	hsa00380	Tryptophan metabolism	4/103	42/7946	0.002042	0.003644	0.001174	IDO1/CYP1A1/MAOA/CAT	4
**131**	hsa04930	Type II diabetes	4/103	46/7946	0.002862	0.005068	0.001633	MAPK1/PRKCD/MAPK8/INS	4
**132**	hsa04514	Cell adhesion molecules (CAMs)	7/103	147/7946	0.002898	0.005093	0.001641	CD80/CD86/ICAM1/SELE/PECAM1/CD40LG/ITGB2	7
**133**	hsa04020	Calcium signaling pathway	8/103	193/7946	0.003476	0.006063	0.001953	NOS2/ADRA1B/ADRB2/PTGER3/NOS3/ADRB1/PRKCB/TBXA2R	8
**134**	hsa05030	Cocaine addiction	4/103	49/7946	0.003607	0.006245	0.002012	RELA/JUN/CREB1/MAOA	4
**135**	hsa00330	Arginine and proline metabolism	4/103	50/7946	0.003882	0.006671	0.002149	NOS2/NOS3/ODC1/MAOA	4
**136**	hsa00140	Steroid hormone biosynthesis	4/103	60/7946	0.007449	0.012707	0.004093	CYP1A1/CYP19A1/AKR1C3/CYP3A4	4
**137**	hsa05150	Staphylococcus aureus infection	5/103	96/7946	0.007995	0.013538	0.004361	C3/C5/ICAM1/C5AR1/ITGB2	5
**138**	hsa04213	Longevity regulating pathway - multiple species	4/103	62/7946	0.008356	0.014047	0.004525	AKT1/INS/SOD1/CAT	4
**139**	hsa04910	Insulin signaling	6/103	137/7946	0.008585	0.014329	0.004616	MAPK1/MAPK8/FASN/AKT1/INS/GSK3B	6
**140**	hsa04750	Inflammatory mediator regulation of TRP channels	5/103	100/7946	0.009457	0.015671	0.005048	IL1B/PRKCD/MAPK8/MAPK14/PRKCB	5
**141**	hsa04960	Aldosterone-regulated sodium reabsorption	3/103	37/7946	0.011942	0.01965	0.00633	MAPK1/INS/PRKCB	3
**142**	hsa04072	Phospholipase D signaling pathway	6/103	148/7946	0.012285	0.020071	0.006466	MAPK1/F2/EGF/AKT1/INS/MS4A2	6
**143**	hsa04080	Neuroactive ligand-receptor interaction	10/103	340/7946	0.012436	0.020175	0.006499	C3/F2/C5/ADRA1B/ADRB2/PTGER3/OPRM1/ADRB1/C5AR1/TBXA2R	10
**144**	hsa04976	Bile secretion	4/103	72/7946	0.013971	0.022509	0.007251	RXRA/CA2/CYP3A4/HMGCR	4
**145**	hsa05204	Chemical	4/103	82/7946	0.021543	0.034469	0.011104	PTGS2/CYP1A1/CYP3A4/GSTP1	4
**146**	hsa05010	Alzheimer disease	6/103	171/7946	0.023401	0.037185	0.011979	MAPK1/CASP3/IL1B/CASP8/GSK3B/BACE1	6
**147**	hsa04340	Hedgehog signaling pathway	3/103	50/7946	0.026705	0.042146	0.013577	CCND1/BCL2/GSK3B	3
**148**	hsa04540	Gap junction	4/103	88/7946	0.027093	0.04247	0.013681	MAPK1/EGF/ADRB1/PRKCB	4
**149**	hsa05322	Systemic lupus erythematosus	5/103	133/7946	0.028959	0.045033	0.014507	CD80/CD86/C3/C5/CD40LG	5
**150**	hsa04970	Salivary secretion	4/103	90/7946	0.029116	0.045033	0.014507	ADRA1B/ADRB2/ADRB1/PRKCB	4
**151**	hsa00220	Arginine biosynthesis	2/103	21/7946	0.029772	0.045742	0.014735	NOS2/NOS3	2

**152**	hsa04666	Fc gamma R-mediated phagocytosis	4/103	93/7946	0.032316	0.049036	0.015796	MAPK1/PRKCD/AKT1/PRKCB	4
**153**	hsa04371	Apelin signaling pathway	5/103	137/7946	0.032338	0.049036	0.015796	NOS2/MAPK1/CCND1/NOS3/AKT1	5

AD: atopic dermatitis. OB: oral bioavailability; DL: drug-likeness
